# Molecular and Physiological Responses of *Citrus sinensis* Leaves to Long-Term Low pH Revealed by RNA-Seq Integrated with Targeted Metabolomics

**DOI:** 10.3390/ijms23105844

**Published:** 2022-05-23

**Authors:** Ning-Wei Lai, Zhi-Chao Zheng, Dan Hua, Jiang Zhang, Huan-Huan Chen, Xin Ye, Zeng-Rong Huang, Jiuxin Guo, Lin-Tong Yang, Li-Song Chen

**Affiliations:** College of Resources and Environment, Fujian Agriculture and Forestry University, Fuzhou 350002, China; lainingwei1109@fafu.edu.cn (N.-W.L.); 1210807031@fafu.edu.cn (Z.-C.Z.); 1210807008@fafu.edu.cn (D.H.); 2190807006@fafu.edu.cn (J.Z.); 2200807011@fafu.edu.cn (H.-H.C.); yexin1000@fafu.edu.cn (X.Y.); huangzengrong@fafu.edu.cn (Z.-R.H.); jxguo@fafu.edu.cn (J.G.); talstoy@fafu.edu.cn (L.-T.Y.)

**Keywords:** aldehydes, *Citrus sinensis*, free amino acids, leaves, low pH, organic acids, reactive oxygen species, RNA-Seq, secondary metabolites, targeted metabolomics

## Abstract

Low pH-induced alterations in gene expression profiles and organic acids (OA) and free amino acid (FAA) abundances were investigated in sweet orange [*Citrus sinensis* (L.) Osbeck cv. Xuegan] leaves. We identified 503 downregulated and 349 upregulated genes in low pH-treated leaves. Further analysis indicated that low pH impaired light reaction and carbon fixation in photosynthetic organisms, thereby lowering photosynthesis in leaves. Low pH reduced carbon and carbohydrate metabolisms, OA biosynthesis and ATP production in leaves. Low pH downregulated the biosynthesis of nitrogen compounds, proteins, and FAAs in leaves, which might be conducive to maintaining energy homeostasis during ATP deprivation. Low pH-treated leaves displayed some adaptive responses to phosphate starvation, including phosphate recycling, lipid remodeling, and phosphate transport, thus enhancing leaf acid-tolerance. Low pH upregulated the expression of some reactive oxygen species (ROS) and aldehyde detoxifying enzyme (peroxidase and superoxidase) genes and the concentrations of some antioxidants (L-tryptophan, L-proline, nicotinic acid, pantothenic acid, and pyroglutamic acid), but it impaired the pentose phosphate pathway and V_E_ and secondary metabolite biosynthesis and downregulated the expression of some ROS and aldehyde detoxifying enzyme (ascorbate peroxidase, aldo-keto reductase, and 2-alkenal reductase) genes and the concentrations of some antioxidants (pyridoxine and γ-aminobutyric acid), thus disturbing the balance between production and detoxification of ROS and aldehydes and causing oxidative damage to leaves.

## 1. Introduction

Acid soils occupy 22.7% of China’s arable land area and occur in 14 provinces [1]. Due to intensive agriculture and monoculture, the acidification effect of nitrogen (N) fertilizer, poor nutrient cycling, acid rain, and soil leaching, topsoil pH in major Chinese croplands has rapidly declined in the past few decades [2,3]. Soil acidification is becoming an increasingly major problem, limiting growth and productivity of crops including *Citrus* [4,5].

There is evidence that low pH (H^+^-toxicity) can directly impair plant root growth and functions, thus damaging root water and nutrient uptake [4,6]. In addition, low pH has an adverse influence on leaf physiological processes, including water and nutrient uptake [4,6,7], photosynthetic pigment biosynthesis, gas exchange [8,9,10], photosynthetic electron transport [4,7], production and detoxification of reactive oxygen species (ROS) and aldehydes [10,11,12], organic acid (OA), carbohydrate and energy metabolisms [13,14], N, protein and amino acid (AA) metabolisms [3,10,14], secondary metabolism, and cell wall biosynthesis [14]. Low pH-induced alterations of these physiological processes should be reflected in gene expression profiles.

RNA-Seq has been widely applied to investigate abiotic stress-induced alterations of gene expression in plants, including aluminum (Al) and heavy metal toxicities, as well as nutrient stresses [14,15,16,17,18]. Although the physiological responses of leaves to low pH have been investigated in some detail, less information was available about low pH-responsive genes. In a study, Zhang et al. [14] used RNA-Seq and metabolomics to investigate low pH effects on gene expression profiles and metabolite abundances in leaves of sweet orange (*Citrus sinensis*) seedlings treated with pH 3.0 and pH 4.8 for 17 weeks and identified 13 upregulated and 87 downregulated genes and nine upregulated and 18 downregulated metabolites in pH 3.0-treated leaves. A total of 44 differentially expressed genes (DEGs) and 14 differentially abundant metabolites (DAMs) were assigned to 29 Kyoto Encyclopedia of Genes and Genomes (KEGG) pathways, including one significantly enriched KEGG pathway [protein processing in endoplasmic reticulum (ko04141)] with a corrected *p* < 0.05 and eight KEGG pathways, respectively. In addition, two reports investigated low pH-responsive genes and metabolites in sweet orange roots [19], and low pH-responsive genes in soybean roots [6], respectively. Although global metabolomic analysis has received great attention in recent years, the application of targeted metabolomics also has advantages in solving biological problems in a more hypothesis-driven way. To accurately quantify metabolites identified by liquid chromatography-mass spectrometry (LC-MS), one must use targeted assays with isotopically labeled standards [20]. Unfortunately, all of these previous studies did not integrate RNA-Seq (transcriptomics) and targeted metabolomics.

*Citrus* can be cultivated in soils covering a wide range of pH and are tolerant to acid soils [4]. Previously, we investigated low pH effects on growth, nutrient and water uptake, carbohydrates, gas exchange, photosynthetic electron transport, and protein expression profiles in leaves; production and detoxification of ROS and methylglyoxal in roots and leaves; and pH-mediated mitigation of copper (Cu) and Al toxicities in sweet orange seedlings [3,4,7,11,13,14,19,21,22]. Based on our findings, we used RNA-Seq and targeted metabolomics to investigate low pH-induced alterations in the expression levels of genes and the abundances of OAs and free AAs (FAAs) in sweet orange leaves. The objectives were (a) to test the hypothesis that extensive reprogramming of gene expression and metabolites occurred in response to low pH in leaves, and (b) to identify the potential metabolic pathways, genes, and/or metabolites responsible for acid (low pH) toxicity and tolerance in leaves.

## 2. Results

### 2.1. RNA-Seq, Mapping and Transcript Assembly in Leaves

Five RNA-Seq libraries from pH 6.0-treated (pH 6.0–1, pH 6.0–2, and pH 6.0–3) and pH 2.5-treated (pH 2.5–1 and pH 2.5–2) leaves were sequenced, generating 46,323,150–61,080,186 raw reads and 6.86–9.00 G clean bases. The higher percentage of clean reads (99.41%–99.73%) and Q3 (94.71%–94.88%) suggested that RNA-Seq data were of high quality and suitable for further analysis. In this study, 72.37%–75.01% (69.69%–72.06% uniquely mapped and 2.67%–3.24% multiply mapped) of the clean reads were mapped to the sweet orange reference genome (Table S1). As shown in Table S2, 15489 known and 361 novel genes were identified in leaves.

### 2.2. Low pH-Responsive Genes in Leaves

**Figure 1:**
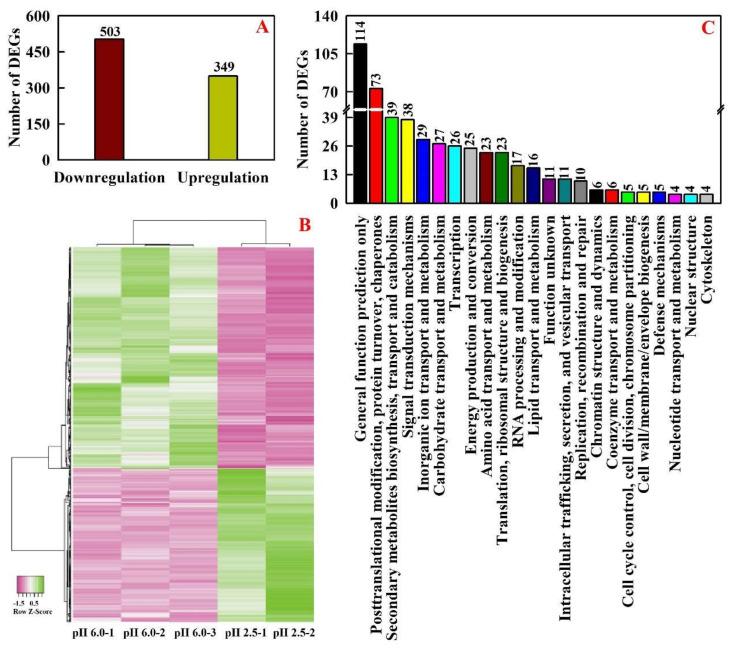
Differentially expressed genes (DEGs) (**A**), cluster analysis (**B**), and euKaryotic Orthologous Groups (KOG) classification (**C**) of DEGs identified in low pH-treated leaves.

We identified 349 upregulated 503 downregulated genes in pH 2.5-treated leaves (Figure 1A and Table S3). Cluster analysis indicated that the general expression profiles of DEGs were highly separated in pH 6.0- and pH 2.5-treated leaves but were highly clustered together in biological replicates per treatment (Figure 1B). All 852 DEGs were submitted to euKaryotic Orthologous Groups (KOG) classification for functional prediction (Figure 1C and Table S4). A total of 478 DEGs were assigned to 23 KOG classifications. General function prediction only (114) contained the most genes, followed by posttranslational modification (PTM), protein turnover, chaperones (73), secondary metabolites (SMs) biosynthesis, transport and catabolism (39), signal transduction mechanisms (38), inorganic ion transport and metabolism (29), carbohydrate transport and metabolism (27), transcription (26), energy production and conversion (25), AA transport and metabolism (23), and translation, ribosomal structure and biogenesis (23).

**Figure 2:**
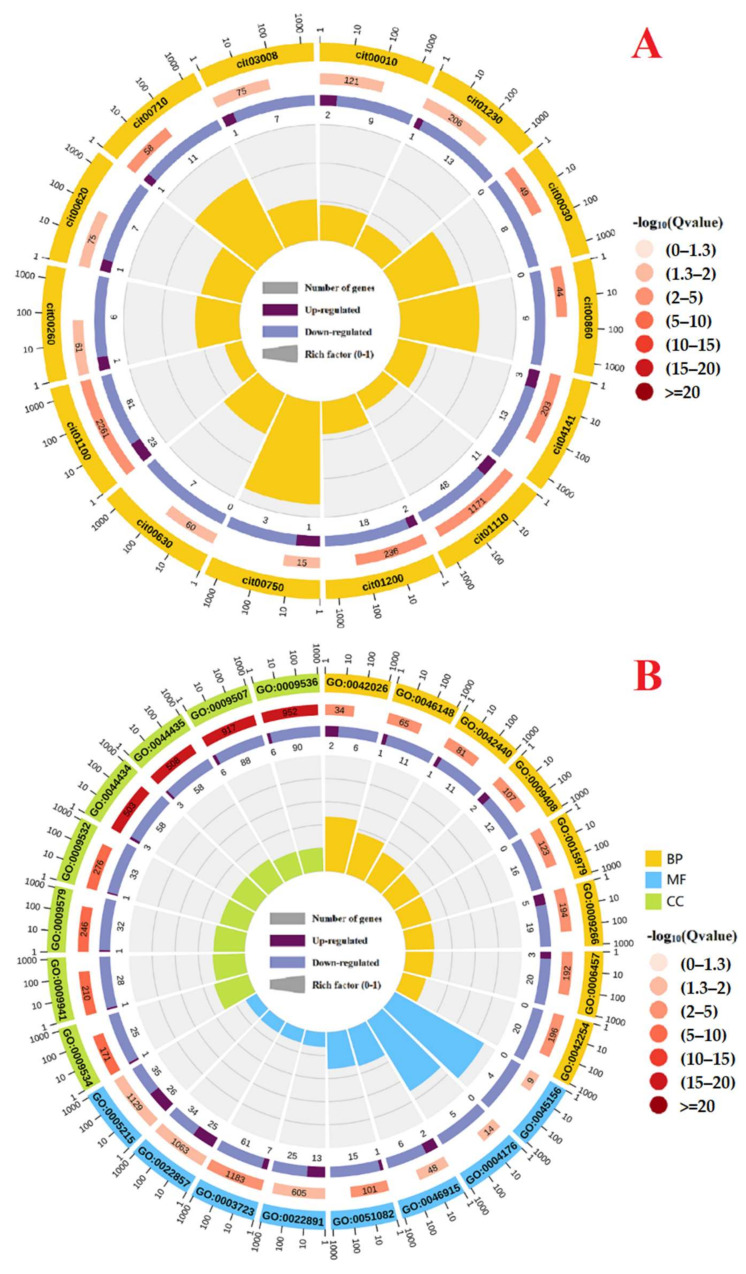
All the significantly enriched 14 KEGG pathways at a corrected *p* < 0.05 (**A**), and the eight most significantly enriched Gene Ontology (GO) terms in biological process (BP), molecular function (MF), and cellular component (CC), respectively (**B**). The circle from outside to inside is presented as follows: the first circle is the enrichment pathway, and the outside of the circle is the coordinate ruler of the number of genes; the second circle is the number of background genes and the -log10(Qvalue); the third circle is the number of upregulated and downregulated genes; the fourth circle is the rich factor value of each pathway (the number of DEGs in the pathway divided by the number of background genes), and each cell of the background auxiliary line represents 0.1.

As shown in Figure 2A and Table S5, 195 DEGs were annotated to 97 KEGG pathways with 14 significantly enriched KEGG pathways with a corrected *p* < 0.05. The top five significantly enriched KEGG pathways were carbon fixation in photosynthetic organisms (cit00710), metabolic pathways (cit01100), porphyrin and chlorophyll metabolism (cit00860), carbon metabolism (cit01200), and biosynthesis of SMs (cit01110).

As shown in Figure 2B and Table S6, 421, 469, and 601 DEGs were annotated to 1630 Gene Ontology (GO) terms in biological process (BP) with 25 significantly enriched GO terms at an adjusted *p* < 0.05, 296 GO terms in cellular component (CC) with 49 significantly enriched GO terms and 661 GO terms in molecular function (MF) with 19 significantly enriched GO terms, respectively. The top five significantly enriched GO terms were response to temperature stimulus GO:0009266), protein folding (GO:0006457), pigment biosynthetic process (GO:0046148), photosynthesis (GO:0015979) and protein refolding (GO:0042026) in BP, chloroplast (GO:0009507), plastid (GO:0009536), chloroplast part (GO:0044434), plastid part (GO:0044435) and thylakoid (GO:0009579) in CC, and unfolded protein binding (GO:0051082), RNA binding (GO:0003723), ATP-dependent peptidase activity (GO:0004176), transmembrane transporter activity (GO:0022857), and transporter activity (GO:0005215) in MF, respectively.

### 2.3. qRT-PCR Validation of DEGs

**Figure 3:**
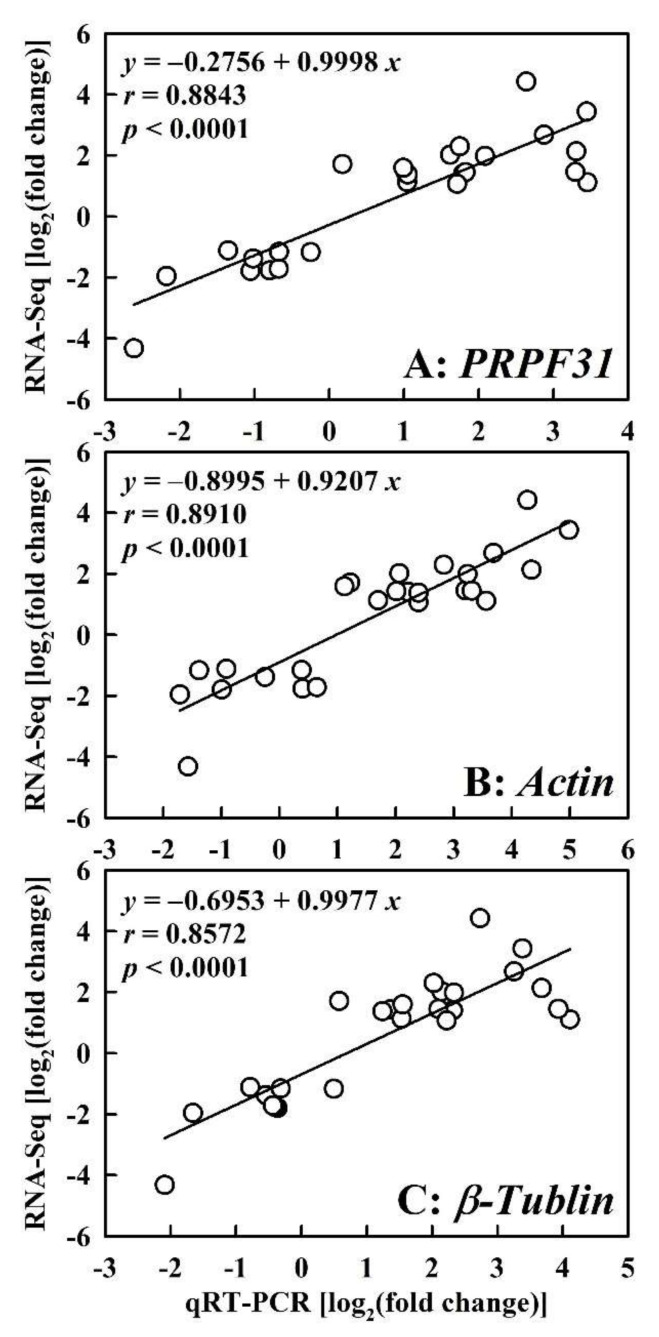
RNA-Seq data in relation to qRT-PCR results using *U4*/*U6 small nuclear ribonucleoprotein PRP31* (*PRPF31* (**A**), *actin* (**B**), and *β*-*tubulin* (**C**) as internal standards. Bars represent means of three biological and two technique replicates. RNA-Seq data from Table S3.

Twenty-six DEGs were randomly selected for qRT-PCR validation. Three house-keeping genes, *U4*/*U6 small nuclear ribonucleoprotein PRP31* (*PRPF31*; Cs7g08440), *actin* (Cs1g05000), and *β*-*tubulin* (Cs6g07480), were chosen as the internal standards. Regressive analysis indicated that there was a significant and positive relationship between qRT-PCR results and RNA-Seq data (Figure 3), meaning that our RNA-Seq data were reliable.

### 2.4. Effects of Low pH on the Concentrations of FAAs and OAs in Leaves

**Figure 4:**
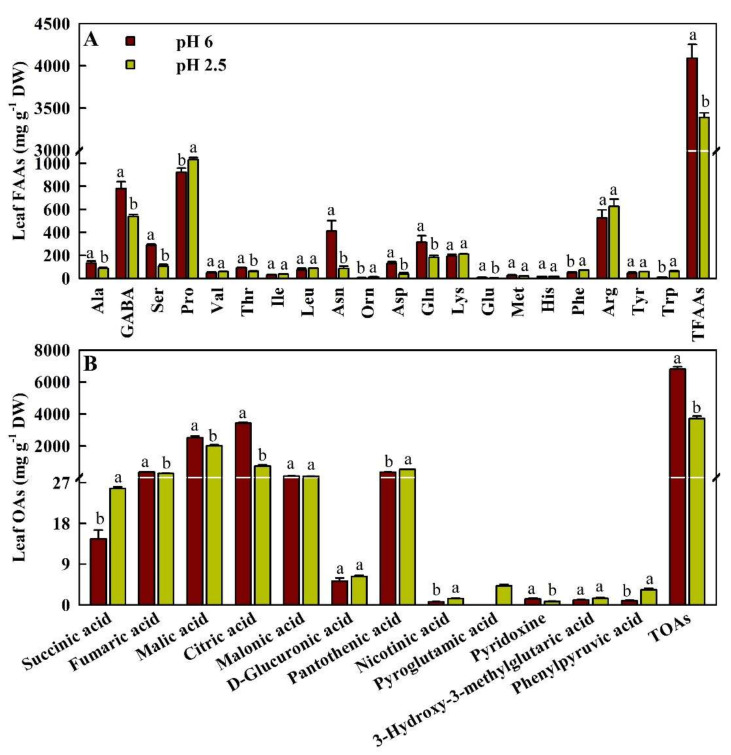
Mean (± SE, *n* = 3) concentrations of 20 free amino acids (FAAs) and total FAAs (TFAAs, the summation of 20 FAAs) (**A**), and 12 organic acids (OAs) and total OAs (TOAs, the summation of 12 OAs) (**B**) detected in pH 2.5 and/or pH 6.0-treated leaves. Pyroglutamic acid (PG) was detected only pH 2.5-treated leaves. Different letters above the bars for the same FAA or OA indicate a significant difference at *p* < 0.05.

As shown in Figure 4A, we used LC-MS to detect 20 FAAs in leaves. Compared with pH 6.0, low pH decreased the concentrations of l-asparagine (Asn), l-aspartic acid (Asp), l-serine (Ser), l-glutamine (Gln), l-alanine (Ala), l-threonine (Thr), γ-aminobutyric acid (GABA), l-glutamic acid (Glu), and total FAAs (TFAAs, the summation of the 20 FAAs) by 78.9%, 71.1%, 63.1%, 42.3%, 35.4%, 33.3%, 31.2%, 19.0%, and 17.2%, respectively, and increased the concentrations of l-tryptophan (Trp), l-ornithine (Orn), l-phenylalanine (Phe), and l-proline (Pro) by 594.3%, 80.0%, 45.2%, and 11.9%, respectively. However, low pH had no significant influence on the concentrations of l-valine (Val), l-isoleucine (Ile), l-leucine (Leu), l-lysine (Lys), l-methionine (Met), histidine (His), l-arginine (Arg), and l-tyrosine (Tyr).

In addition, we used LC-MS to detect 12 OAs in pH 2.5- and/or pH 6.0-treated leaves (Figure 4B). Low pH increased the concentrations of phenylpyruvic acid (PPA), nicotinic acid (NA), succinic acid, and pantothenic acid (PA) by 269.4%, 111.3%, 76.1%, and 48.1%, respectively, and reduced the concentrations of citric acid, pyridoxine (PN), fumaric acid, malic acid, and TOAs by 78.3%, 40.7%, 24.5%, 19.6%, and 45.4%, respectively. Pyroglutamic acid (PG) was detected only in pH 2.5-treated leaves. Low pH did not significantly affect the concentrations of malonic acid, d-glucuronic acid, and 3-hydroxy-3-methylglutaric acid.

## 3. Discussion

### 3.1. Low pH Altered the Expression of Genes Related to Photosynthesis, Carbon, Carbohydrate, and Energy Metabolisms in Leaves

As shown in Table S7, we obtained 38 downregulated and two upregulated [*phosphoenolpyruvate* (*PEP*) *carboxykinase* (*ATP*) *1* (*PCK1*; Cs1g20920) and *zeta-carotene desaturase, chloroplastic*/*chromoplastic* (*ZDS1*; orange1.1t06069)] genes involved in porphyrin and chlorophyll (Chl) metabolism (cit00860; nine downregulated genes), carotenoid (Car) biosynthesis (cit00906; four downregulated genes and one upregulated *ZDS1*), photosynthesis (GO:0015979; 16 downregulated genes), photosynthesis antenna proteins (cit00196; two downregulated genes), photosynthetic electron transport chain (PETC, GO:0009767; six downregulated genes), photosynthesis, light reaction (GO:0019684; 10 downregulated genes), and carbon fixation in photosynthetic organisms (cit00710; 11 downregulated genes and one upregulated *PCK1*) in low pH-treated leaves. All these KEGG pathways and GO terms were significantly enriched at a corrected or an adjusted *p* < 0.05, except for photosynthesis antenna proteins and Car biosynthesis. These results suggested that low pH impaired Chl and Car biosynthesis, light reaction (light harvesting and PETC), and carbon fixation in photosynthetic organisms, thereby lowering photosynthetic pigment levels and CO_2_ assimilation (A_CO2_) in leaves. This was supported by the reports that low pH impaired the whole PETC and lowered the levels of Chl and Car, the rates of photosynthesis [4], and the abundances of many proteins involved in Chl biosynthesis, photosynthetic electron transport, ribulose bisphosphate carboxylase/oxygenase (Rubisco) activation, and Calvin cycle and carbon fixation in low pH-treated sweet orange leaves [3]. In the biosynthesis pathway of Car, ZDS and carotenoid isomerase (CRTISO) catalyze the conversion of ζ-carotene to lycopene, the precursor of ABA. Overexpression of *IbZDS* increased the concentration of ABA in transgenic sweetpotato relative to wild type (WT) plants [23]. Yuan et al. [24] reported that low pH increased the ABA level in *Atractylodes lancea* roots. The upregulation of *ZDS1* suggested that low pH might increase ABA biosynthesis and accumulation in leaves, thus stimulating stomatal closure, as observed by decreased stomatal conductance [4]. This was also supported by our results that two genes (Cs1g13200 and Cs5g04280) involved in stomatal movement (GO:0010118) were downregulated in low pH-treated leaves (Table S7). Yan et al. [25] reported that exogenous ABA might alleviate low pH-induced inhibition of corn and broad bean root growth. PEP carboxylase (PEPC) catalyzes the irreversible β-carboxylation of PEP to yield oxaloacetate (OAA) and inorganic phosphate (Pi). PCK catalyzes the reversible reaction: OAA + ATP ↔ PEP + ADP + CO_2_. The bypass of the pyruvate kinase (PK) step in glycolysis via induction of PEPC is one of the metabolic responses to Pi deficiency [26,27]. Pi-deficiency-induction upregulation of *PCK1* could be explained in this way, because phosphorus (P) level was greatly reduced in pH 2.5-treated leaves [4]. In plant tissues, PCK is also induced by acidification and is believed to play a role in the regulation of pH [28]. Thus, the upregulation of *ZDS1* and *PCK1* might be an adaptive strategy of leaves to low pH. Unexpectedly, *PEPC 1* (Cs2g15520) expression was downregulated in low pH-treated leaves (Table S7). However, PEPC activity was increased in low pH-treated sweet orange leaves [13]. This implied that PEPC activity could not be regulated in the transcriptional level.

When plants are exposed to adverse conditions, they usually suffer from energy starvation. There is an intimate relationship between stress and energy availability. In order to maintain an appropriate metabolic balance under adverse conditions, plant growth will slow down to improve the accumulation of carbohydrates [29]. In addition to supplying energy, carbohydrates, especially soluble sugars, can also be used as plant osmotic protectants, antioxidants, and signaling molecules. If necessary, starch can be immediately hydrolyzed into soluble sugar [30]. Recent studies showed that low pH increased the levels of glucose, fructose, sucrose, starch, and total nonstructural carbohydrates (TNC, the summation of starch + sucrose + fructose + glucose), decreased A_CO2_, and affected the levels of malate, citrate, and isocitrate and the activities of acid-metabolizing enzymes in sweet orange leaves [4,13]. Thus, the expression of genes related to carbon, carbohydrate, and energy metabolisms should be changed in low pH-treated leaves. As expected, we identified 40 downregulated and four upregulated [*CYP enzymes assisting alcohol dehydrogenase* (*CYPADH*; Cs7g14430), *PCK1, soluble inorganic pyrophosphatase 4* (*PPiase4*; Cs6g05740) and *NADH*-*ubiquinone oxidoreductase chain 4* (*ND4*; orange1.1t06026)] genes involved in carbon metabolism (cit01200; 18 downregulated genes and upregulated *CYPADH* and *PCK1*), starch and sucrose metabolism (cit00500; five downregulated genes), pentose Pi pathway (PPP, cit00030; eight downregulated genes), glycolysis/gluconeogenesis (cit00010; nine downregulated genes and upregulated *CYPADH* and *PCK1*), pyruvate metabolism (cit00620; seven downregulated genes and upregulated *CYPADH*), glyoxylate and dicarboxylate metabolism (cit00630; seven downregulated genes), citrate cycle (TCA cycle, cit00020; four downregulated genes and upregulated *PCK1*), oxidative phosphorylation (cit00190; four downregulated genes and upregulated *PPiase4*), ATP biosynthetic process [GO:0006754; downregulated *ATP synthase gamma chain, chloroplastic* (Cs2g03080)], ATP generation from ADP [GO:0006757; downregulated *phosphoglycerate kinase, chloroplastic* (orange1.1t03280)], generation of precursor metabolites and energy (GO:0006091; 16 downregulated genes and upregulated *ND4*) and energy derivation by oxidation of organic compounds (GO:0015980; five downregulated genes and upregulated *ND4*) in low pH-treated leaves (Table S8). In addition, low pH reduced the concentrations of fumaric acid, malic acid, citric acid, PN, and TOAs in leaves (Figure 4B). Obviously, low pH reduced carbon and carbohydrate metabolisms, as well as the production of energy (ATP) in leaves. This agreed with the reports that ATP production and accumulation were reduced in drought and Pi-deficient leaves [31,32], because low pH reduced relative water content (RWC) and P concentration in leaves [4]. In low pH-treated leaves, the accumulation of TNC occurred despite reduced A_CO2_ [3,4], because they were less used due to decreased growth [4], as indicated by the decreased biosynthesis of OAs and the accumulation of TOAs (Figure 4B).

OAs can act as carbon precursors for AA biosynthesis. The reduced demand for carbon skeleton precursors due to reduced biosynthesis of AAs and proteins might contribute to the accumulation of TNC, because the levels of TFAAs (Figure 4A) and total soluble proteins [4] were reduced in low pH-treated leaves. However, the levels of PPA, NA, succinic acid, PA, and PG were increased in low pH-treated leaves (Figure 4B). Succinic acid is the primary intermediate of TCA cycle, which plays a key role in the production of ATP. By over-biosynthesis of succinic acid, mitochondria can accumulate more ATP for adverse conditions [33]. Ullah et al. [34] showed that a drought-induced increase of succinate levels in drought-tolerant triticeae (TR39477) leaves and roots was associated with the efficient use of TCA cycle to produce more energy under drought stress. The observed higher concentration of succinic acid can be explained in this way, because low pH reduced RWC in sweet orange leaves [4].

PPA is the intermediate in the shikimic acid pathway for the biosynthesis of the key aromatic AAs, Tyr, and Phe in plants. Zandalinas et al. [35] found that drought, heat, and a combination of drought and heat increased and decreased the concentration of PPA in tolerant Carrizo citrange and non-tolerant Cleopatra mandarin leaves, respectively.

PG is involved in the glutathione cycle. Jiménez-Arias et al. [36] reported that PG could induce lettuce plants’ tolerance to drought by improving antioxidant defenses and photosynthesis. Low pH-induced accumulation of PG (Figure 4B) agreed with the report that the concentration of PG in leaves of Cleopatra plants (self-grafted) increased when exposed to drought, heat, or drought and heat combination [37].

PA (vitamin B_5_, VB_5_) belongs to vitamin B group and is an important antioxidant. It can stimulate the biosynthesis of coenzyme A (CoA) and acyl-carrier protein. CoA can lower the level of *tert*-butyl hydroperoxide (a ROS generator), and increase active state respiration, the rate of ATP biosynthesis, the level of total glutathione level, and the ratio of reduced glutathione (GSH) to oxidized glutathione (GSSG) in *Ehrlich ascites* tumor cells [38]. Interestingly, we observed that the expression of *PANK2* (Cs5g05515) encoding pantothenate kinase 2, an enzyme catalyzing the phosphorylation of PA, the first step in CoA biosynthesis, was reduced in low pH-treated leaves (Table S3). Tilton et al. [39] reported that AtPANK1 and AtPANK2 had complementary roles in *Arabidopsis*, and that in silique tissue, CoA concentrations reduced only 18% and 12% in *pank1-1* and *pank2-1* mutants, respectively. Thus, the downregulation of *PANK2* did not necessarily imply that the level of CoA was greatly reduced in low pH-treated leaves.

In living organisms, nicotinamide (NAM) adenine dinucleotide (NAD) is biosynthesized either by a de novo pathway or by a salvage pathway. NA can serve as a salvage pathway NAD precursor and in this way counteract the decrease of NAD caused by oxidative stress. A reduction in NAD degradation may enhance an efficient energy homeostasis and tolerance to oxidative stress [40]. Ahmad et al. [41] reported that overexpression of *nicotinamidase 3* (*NIC3*), which functions in the conversion of NAM to NA in the NAD salvage pathway, conferred drought tolerance and increased NA accumulation in *Arabidopsis*, and an exogenous application of NA also enhanced *Arabidopsis* drought tolerance. The low pH-induced leaf accumulation of NA agreed with the report that Pi-deficiency increased the concentration of NA in soybean shoots [42]. 

The upregulation of *CYPADH* involved in strictosidine biosynthesis suggested that the biosynthesis and accumulation of strictosidine were improved in low pH-treated leaves [43]. Strictosidine is the precursor of many terpenoid indole alkaloids in plants. Zhu et al. [44] showed that alkaloids (ajmalicine, vindoline, catharanthine, and strictosidine) in *Catharanthus roseus* leaves were increased after UV-B irradiation and dark incubation to improve the scavenging capacity of ROS. 

PPiases, which catalyze the hydrolysis of pyrophosphate (PPi, a Pi donor and energy source) to Pi, play a role in plant tolerance to Pi-deficiency [45]. NADH-ubiquinone oxidoreductase is the core subunit of the mitochondrial membrane respiratory chain NADH dehydrogenase (complex I). The upregulation of *ND4* might improve respiration and provide the energy to maintain basic metabolic processes in low pH-treated leaves with decreased A_CO2_ [46]. Thus, the low pH-induced increases of PA, PG, PPA, NA, and succinic acid and the upregulation of *PCK1, PPiase4*, *ND4*, and *CYPADH* might be the adaptive responses of leaves to low pH.

### 3.2. Low pH Altered the Expression of Genes Related to N, Protein, and AA Metabolisms in Leaves

Because low pH reduced the concentrations of N, total soluble proteins, and TFAAs, and affected the levels of individual FAA in leaves (Figure 4A), the expression of genes related to N should be altered in low pH-treated leaves. As shown in Table S9, we obtained 120 downregulated and 41 upregulated genes involved in N and protein metabolisms in low pH-treated leaves, including three downregulated and one upregulated genes in the N metabolism (cit00910), 60 downregulated and 18 upregulated genes in the cellular N compound biosynthetic process (GO:0044271), two upregulated genes in the cellular N compound catabolic process (GO:0044270), 44 downregulated and 16 upregulated genes in the protein metabolic process (GO:0019538), 11 downregulated and three upregulated genes in the protein catabolic process (GO:0030163), one downregulated and four upregulated genes in the protein ubiquitination (GO:0016567), three downregulated and two upregulated genes in the proteasome-mediated ubiquitin-dependent protein catabolic process (GO:0043161), five downregulated and two upregulated genes in the ubiquitin-dependent protein catabolic process (GO:0006511), one downregulated and nine upregulated genes in the protein phosphorylation (GO:0006468), one downregulated gene in the protein dephosphorylation (GO:0006470), 13 downregulated and three upregulated genes in the protein processing in endoplasmic reticulum (cit04141), 20 downregulated and three downregulated genes in the protein folding (GO:0006457), 20 downregulated genes in the ribosome biogenesis (GO:0042254), and three downregulated genes in the aminoacyl-tRNA biosynthesis (cit00970). Generally viewed, low pH decreased and increased the biosynthesis and catabolism of N compounds, respectively, thus lowering the accumulation of N compounds in low pH-treated leaves, as indicated by the decreased level of N [4]. In contrast, low pH impaired protein processing and folding and reduced protein translation and catabolism in leaves. Thus, the low pH-induced decrease in protein concentration in leaves [4] was caused by reduced biosynthesis rather than by increased degradation.

Heat shock proteins (HSPs) and/or molecular chaperones undertake a function in protecting plants from abiotic stresses by preventing protein misfolding, denaturing, agglomeration, and degradation, as well as by maintaining transportation of newly synthesized proteins across cell organelles and cellular homeostasis. Protein degradation not only provides respiratory substrates for plants, but also triggers adaptive responses to abiotic stresses by redistributing nutrients from nonessential metabolic areas to key cellular activities [47]. Plant proteases play a key role in the selective degradation of specific proteins and the strict control of protein quality under stressed conditions. Both inactive (i.e., misfolded) and futile proteins are ubiquitinated and degraded [48]. Here, we identified 18 downregulated and two upregulated *HSPs* (*chaperones*) related to the protein folding, and 13 downregulated and three upregulated genes involved in the protein catabolic process, the proteasome-mediated ubiquitin-dependent protein catabolic process, and the ubiquitin-dependent protein catabolic process in low pH-treated leaves (Table S9). The downregulation of these genes might impair N protein metabolism, thus lowering leaf acid-tolerance. There is evidence that PTMs, such as ubiquitination, phosphorylation, and dephosphorylation, play a key role in the abiotic stress response of plants [49]. Here, the expression of genes related to ubiquitination and phosphorylation were induced in low pH-treated leaves. In addition, we identified one downregulated *ABA receptor PYL4* (Cs7g30500) involved in the inhibition of group-A protein phosphatases type 2C (PP2Cs) activity in low pH-treated leaves [50]. The downregulation of *PYL4* agreed with the above inference that low pH increased ABA accumulation in leaves, because *PYL4* was strongly repressed in ABA-treated *Arabidopsis* seedlings [50]. These results suggested that low pH activated protein ubiquitination, phosphorylation, and dephosphorylation, thus enhancing leaf acid-tolerance.

As shown in Table S9, we identified 26 downregulated and four upregulated genes involved in the AA metabolism in low pH-treated leaves, including 12 downregulated genes and one upregulated gene in the biosynthesis of AAs (cit01230), two downregulated in the cellular AA catabolic process (GO:0009063), seven downregulated genes and one upregulated gene in glycine, serine, and threonine metabolism (cit00260), eight downregulated genes in cysteine and methionine metabolism (cit00270), five downregulated genes in alanine, aspartate, and glutamate metabolism (cit00250), three downregulated genes in arginine biosynthesis (cit00220), one downregulated gene and three upregulated genes in tyrosine metabolism (cit00350), one downregulated gene in lysine biosynthesis(cit00300), one downregulated gene and one upregulated gene in phenylalanine, tyrosine and tryptophan biosynthesis (cit00400), one downregulated gene in phenylalanine metabolism (cit00360), one downregulated gene in histidine metabolism (cit00340), two downregulated genes in tryptophan metabolism (cit00380), and two downregulated genes in arginine and proline metabolism (cit00330). This implied that both the biosynthesis and catabolism of AAs were reduced in low pH-treated leaves. Thus, the reduction of Asn, Asp, Ser, Gln, Ala, Thr, GABA, Glu, and TFAAs in low pH-treated leaves was mainly caused by decreased biosynthesis rather than by increased degradation and utilization, because the concentration of total soluble proteins was reduced in leaves [4]. Low pH-induced decreases in the concentrations of proteins and TFAAs have been obtained in *Pennisetum clandestinum* [51], sweet orange [4,14], and *Calopogonium mucunoides* [52] leaves.

Interestingly, the levels of Trp, Orn, Phe, and Pro in leaves were increased by low pH (Figure 4A). This agreed with the reports that low pH increased the concentration of Pro in wheat leaves at grain filling [12] and pea leaves [53], that drought increased the levels of Phe, Trp, and Pro in leaves of maize hybrids [54], and that Cu-toxicity improved the levels of Phe, Trp, indole-3-acetic acid (IAA), and total auxins in *C. grandis* leaves [17,55]. In plants, Pro is biosynthesized by two pathways, the Orn and Glu pathways. Here, we identified five downregulated [*phospho-2-dehydro-3-deoxyheptonate aldolase 2, chloroplastic* (*DHS2*; orange1.1t02000); *primary amine oxidase* (Cs9g06720); *aldehyde dehydrogenase (ALDH) family 3 member H1* (*ALDH3H1* or *ALDH4*; Cs3g19820); *dihydrolipoyl dehydrogenase* (*dihydrolipoamide dehydrogenase*) *1, mitochondrial* (Cs9g01670; *mtLPD1*), and *S-adenosylmethionine decarboxylase (SAMDC) proenzyme* (*SAMDCP*; Cs7g12410)] and two upregulated [*arogenate dehydrogenase 1, chloroplastic* (*TYRAAT1*; Cs7g30350), and *primary amine oxidase 2* (Cs9g06700)] genes involved in phenylalanine, tyrosine, and tryptophan biosynthesis (*DHS2* and *TYRAAT1*), phenylalanine metabolism (Cs9g06720 and Cs9g06700), tryptophan metabolism (*ALDH4* and *mtLPD1*), and arginine and proline metabolism (*ASMDCP* and *ALDH4*) in low pH-treated leaves (Table S9). DHS2 was involved in the biosynthesis of chorismate, the precursor of Trp, Phe, and Tyr. ALDH is involved in the catabolism of Pro and Trp. ASMDC functions in the biosynthesis of Pro, spermidine, and spermine [56]. These results suggested that low pH-induced accumulation of Trp, Phe, and Pro was related to the decreased degradation rather than to the increased biosynthesis.

Orn is the precursor for Pro biosynthesis. Low pH-induced accumulation of Pro might be caused by an increased Orn pathway, as indicated by the increased concentration of Orn (Figure 4A). PPA can serve as a precursor for Phe biosynthesis and vice versa. Low pH-induced accumulation of Phe might be related to increased accumulation of PPA in leaves (Figure 4). Pro plays a key role in plant stress tolerance by stabilizing the antioxidant system through osmotic adjustment and protecting the integrity of cell membranes, acting as a molecular chaperone, and protecting the integrity of proteins and enzymes. Naing et al. [57] showed that overexpression of *RsMYB1* conferred anthocyanin-enriched transgenetic petunia lines low-pH tolerance by upregulating the expression of genes related to Pro and antioxidant production. Alotaibi et al. [58] reported that arbuscular mycorrhizae-mediated mitigation of Al-toxicity in barley and lotus plants grown in acid soil was associated with increased accumulation of Pro due to increased biosynthesis and decreased degradation. Liu et al. [56] demonstrated that overexpression of *SAMDC* conferred *Arabidopsis* salt-tolerance by improving Pro and polyamine levels. Aromatic AAs (Phe, Trp, and Tyr) can act as precursors for many SMs and various phytohormones, such as auxin and salicylate, that play a key role in the stress tolerance of plants [59]. Exogenous application of Phe enhanced the tolerance of tomato fruits to chilling via ensuring the supply of NADPH for activation of antioxidant systems and maintaining membrane integrity [60]. Foliar application of Trp and salicylic acid conferred maize drought tolerance by improving RWC and maintaining membrane integrity in leaves [61]. Thus, the low pH-induced accumulation of Pro, Phe, Trp, and Orn in leaves might contribute to the acid-tolerance of leaves.

To conclude, low pH downregulated the biosynthesis of N compounds, proteins, and FAAs in leaves. This might be helpful for low pH-treated leaves in maintaining energy homeostasis during ATP deprivation, because inorganic N assimilation and biosynthesis of N-containing compounds require photosynthesis or carbohydrate degradation to provide reducing power, energy (ATP), and carbon [62].

### 3.3. Low Pi-Responsive Genes Displayed Adaptive Responses to Low pH in Leaves

Low pH reduced P uptake and concentrations in sweet orange leaves, stems, and roots [4]. Pi starvation can trigger adaptive metabolic responses to maintain cellular Pi homeostasis in plants [63]. Pi remobilization from organic-P containing compounds plays a role in Pi homeostasis [14]. Here, we identified 12 downregulated genes and one upregulated [*phosphatidylcholine*:*diacylglycerol cholinephosphotransferase 1* (*PDCT1*, also known as *ROD1*; Cs1g12410)] gene involved in the organophosphate biosynthetic process (GO:0090407), and one upregulated *glycerophosphodiester phosphodiesterase GDPD3* (Cs7g02950) involved in the organophosphate catabolic process (GO:0046434) in low pH-treated leaves (Table S10), implying that low pH reduced the accumulation of organic-P-containing compounds in leaves due to reduced biosynthesis and increased degradation.

One strategy for plants to acquire Pi under Pi-deprivation conditions is to replace membrane phospholipids (a Pi reserve) with non-P membrane lipids [14]. Here, we obtained two downregulated [*geranylgeranyl diphosphate reductase, chloroplastic* (*CHLP*; Cs5g10740) and *4-hydroxy-3-methylbut-2-enyl diphosphate reductase, chloroplastic* (*ISPH*; Cs5g28200)] and five upregulated [*GDPD3*, *PDCT1*, *mitochondrial translocator assembly and maintenance protein 41* (*TAM41*; Cs2g11760) and *O-acyltransferase WSD1* (Cs2g23350)] genes involved in the phospholipid catabolic process (GO:0009395; *GDPD3*), the phospholipid biosynthetic process (GO:0008654; *CHLP*, *ISPH*, *PDCT1* and *TAM41*), the glycerolipid catabolic process (GO:0046503; *GDPD3*), and the glycerolipid biosynthetic process (GO:0045017; *PDCT1*, *TAM41* and *WSD1*) in low pH-treated leaves (Table S10). In *Arabidopsis*, Cheng et al. [64] indicated that Pi-deficiency-induced upregulation of *GDPDs* might be involved in the maintenance of cellular Pi homeostasis by promoting the release of Pi in phospholipids during Pi-deficiency. PDCT catalyzes the interconversion between phosphatidylcholine and diacylglycerol (DAG). Overexpression of *PDCT1* increased the accumulation of hydroxy fatty acids (HFAs) in triacylglycerol by promoting the conversion of HFA-containing phosphatidylcholine into DAG in *Arabidopsis* seeds [65]. These results indicated that low pH might reduce the accumulation of phospholipids due to decreased biosynthesis and increased degradation, and increase the accumulation of glycerolipid due to increased biosynthesis.

In addition, we identified two downregulated [*vacuolar cation/proton exchanger 3* (*CAX3*; Cs6g08320) and *sodium-dependent Pi transport protein 1, chloroplastic* (*ANTR1*, also known as *PHT4;1*; Cs8g19980)] and five upregulated [*PPiase1* (Cs1g18540), *PCK1*, *SPX domain-containing protein 2* (*SPX2*; Cs4g17870), *GDPD3* and *ABC transporter I family member 17* (*ABCI17*, also known as *STAR1*; Cs4g04420)] genes involved in cellular response to Pi starvation (GO:0016036; *PPiase 1*, *PCK1* and *SPX2*), Pi ion homeostasis (GO:0055062; *CAX3* and *GDPD3*), and inorganic Pi transmembrane transporter activity (GO:0005315; *ABCI17* and *ANTR1*), as well as two other upregulated [*purple acid phosphatase (PAP) 8* (*PAP8*; Cs2g17940) and *ribonuclease (RNase) 2* (*RNS2*; Cs2g18380)] low-Pi-responsive genes in low pH-treated leaves (Table S10). Pi-deficiency-induced upregulation of *PPiase1* has been shown to play a role in Pi homeostasis by catalyzing the cleavage of PPi in *Arabidopsis* [66]. Evidence shows that SPX2 played a role in Pi homeostasis by interacting with Pi starvation response regulator 2 (PHR2) in a Pi-dependent manner [67].

CAX3 is involved in Pi homeostasis associated with CAX1. In *Arabidopsis*, *cax1*/*cax3* double mutant had increased concentration of Pi in shoots and upregulated expression of genes encoding SPX domain-containing proteins, *SPX1* and *SPX3* [68]. Low pH-induced upregulation of *STAR1* in leaves agreed with the reports that *STAR1* was activated by the reduced concentration of Pi in the cytosol [69] and by Al-toxicity [70]. However, the expression level of *STAR1* in rice root was not higher at pH 4.5 than at pH 5.5 [70]. *Arabidopsis atstar1* mutant was more sensitive to Pi-deficiency than WT, as indicated by a greater Pi-deficiency-induced inhibition of root growth [71]). ANTR1 has been shown to export Pi from the thylakoid lumen, which contains enzymatic ATP-consuming reactions related to photosystem II (PSII) repair [72]. Karlsson et al. [73] indicated that the absence of *PHT4;1* altered the pH gradient across the thylakoid membrane, leading to a faster activation of photoprotective mechanisms [non-photochemical quenching (NPQ)], and decreased the availability of Pi in the stroma, resulting in a decrease in ATP synthesis. The downregulation of *ANTR1* in low pH-treated leaves agreed with the report that low pH increased dissipated energy per reaction center (cross section) and quantum yield for energy dissipation in leaves [4,7], and with the above inference that ATP biosynthesis was reduced in low pH-treated leaves.

PAPs are involved in Pi acquisition and recycling in plants. Gho et al. [74] found that Pi-starvation increased the expression of *OsRNS* and the activities of RNase and reduced the concentrations of total RNA in rice shoots and roots, concluding that Pi-starvation-inducible S-like RNase genes were involved in Pi recycling by RNA degradation. Rojas et al. [75] indicated that a class III non-S-RNase gene *NnSR1* in *Nicotiana alata* shoots and roots was induced by Pi-deficiency, and that NnSR1 might contribute to Pi mobilization from RNA.

To conclude, we identified 13 downregulated and 10 upregulated low-Pi-responsive genes in low pH-treated leaves. Low pH-treated leaves displayed some adaptive responses to Pi starvation, such as Pi recycling, lipid remodeling (replacement of membrane phospholipids with non-P membrane lipids), and Pi transport, thus enhancing leaf acid-tolerance.

### 3.4. Low pH Reduced SM Biosynthesis and Accumulation in Leaves

We identified 49 downregulated and 10 upregulated genes in the biosynthesis of SMs (cit01110) involved in low pH-treated leaves (Table S11). The phenylpropanoid pathway plays a key role in the biosynthesis of SMs. Here, we obtained four downregulated [*β-glucosidase 13* (*BGLU13*; Cs7g01350), *shikimate O-hydroxycinnamoyltransferase* (*HCT*; Cs4g02360), *caffeic acid 3-O-methyltransferase* (*OMT*; Cs1g22590), *flavone 3’-O-methyltransferase 1* (*OMT1*; orange1.1t02947)] and two upregulated [*berberine bridge enzyme (BBE)-like 13* (*BBE-like 13*; Cs2g10070) and *peroxidase 47* (*PER47*; Cs1g19540)] genes involved in the phenylpropanoid biosynthesis (cit00940) in low pH-treated leaves (Table S11). These results implied that SM biosynthesis was downregulated in these leaves. This agreed with the report that SM biosynthesis might be downregulated in pH 3.0-treated sweet orange leaves relative to pH 4.8-treated leaves, as indicated by 11 downregulated and six upregulated SMs [14]. It is worth noting that only three downregulated and three upregulated genes related to the biosynthesis of SMs, and two downregulated genes related to phenylpropanoid biosynthesis were identified in in pH 3.0-treated sweet orange leaves. This might be related to higher pH, because the adverse effects of pH 3.0 on sweet orange leaves were much less than that of pH 2.5 [4]. Aromatic AAs can act as precursors for the biosynthesis of various SMs. The downregulation of SM biosynthesis indicated that the demand for these aromatic AA precursors was reduced in low pH-treated leaves, thus contributing to the accumulation of aromatic AAs in these leaves, as indicated by increased concentrations of Phe and Trp and unaltered concentration (Figure 4A).

Lignin is a phenylpropanoid-derived polymer from the oxidative polymerization of three monolignols, which are biosynthesized from Phe via a specialized branch of phenylpropanoid biosynthesis. Except for *BGLU13*, the other five DEGs related to phenylpropanoid biosynthesis were involved in lignin biosynthesis. BBE-like enzyme catalyzes the oxidation of monolignols to their corresponding aldehydes, which together with monolignols are the precursors of lignin biosynthesis. Then, these compounds are polymerized to form lignin by peroxidases and laccases in the secondary cell wall [76]. In *Arabidopsis*, Klason lignin was not lower (higher) in *omt1* mutant (*OMT1* overexpressed line) than in WT. OMT could not be a limiting enzyme for S-unit biosynthesis [77]. Besseau et al. [78] found that *HCT*-silenced *Arabidopsis* plants had a decreased lignin level, but an increased flavonoid level. These results implied that lignin biosynthesis was not inhibited in low pH-treated leaves. This agreed with the report that lignin concentration in sweet orange leaves was not lower at pH 3.0 than at pH 4.8, although more downregulated (11) than upregulated (6) SMs were identified in pH 3.0-treated leaves [14].

VB_6_ is a collection of six different related molecules: PN, pyridoxamine, pyridoxal, and their phosphorylated derivatives, pyridoxine 5’-Pi, pyridoxamine 5’-Pi, and pyridoxal 5’-Pi, respectively [79]. Here, we identified three downregulated genes [*pyridoxal 5’-Pi synthase-like subunit PDX1.2* (*PDX12*; Cs1g14970), *probable pyridoxal 5’-Pi synthase subunit PDX1* (*PDX1*, Cs8g20230), and *threonine synthase 1, chloroplastic* (*TS1*, Cs4g15090)] and one upregulated (*PPiase1*) gene involved in VB_6_ metabolism (cit00750) in low pH-treated leaves (Table S11). The downregulation of *PDX12* and *PDX1* implied that VB_6_ biosynthesis was inhibited in low pH-treated leaves. This was supported by our finding that low pH reduced PN concentration in leaves (Figure 4B).

The main function of SMs is considered to promote the growth and survival of plants under stress. In this study, we obtained 51 downregulated and 12 upregulated genes related to SM metabolism in low pH-treated leaves, implying that low pH reduced SM biosynthesis and accumulation, thus lowering leaf acid-tolerance. This agreed with the reports that increased pH-mediated alleviation of Cu-toxicity in leaves and Al-toxicity in roots of sweet orange seedlings involved increased SM biosynthesis and accumulation [14,19].

### 3.5. Low pH Broke the Balance between Production and Detoxification of ROS and Aldehydes, thus Leading to Oxidative Damage in Leaves

Low pH can stimulate the generation of ROS, thereby causing oxidative damage in a plant cell. In addition, it can induce the production of aldehydes [4,22]. Increased accumulation of aldehydes can lead to oxidative damage in a plant cell [80]. Therefore, both enzymatic [peroxidase, ascorbate (ASC) peroxidase (APX), glutathione S-transferase (GST) and superoxide dismutase (SOD)] and non-enzymatic (ASC, GSH, vitamins and Trp) antioxidant systems might be altered in low pH-treated leaves to meet the increased demand for the scavenging of ROS and aldehydes. As expected, we identified 25 downregulated and eight upregulated [*PER47*, *SOD [Cu-Zn] 1* (*SODCC.1*, Cs3g12000), *SOD [Cu-Zn]*, *chloroplastic* (*SODCP*, Cs8g15520), *glutathione gamma-glutamylcysteinyltransferase 2* (*PCS2*; Cs5g29460), *sulfate transporter 2.1* (*SULTR2;1*, Cs1g01730), *glucuronokinase 1* (*GLCAK1*; Cs2g27180), *PPiase1* and *GST zeta class* (*GSTZ*; Cs3g01240)] genes related to ROS and aldehyde detoxification in low pH-treated leaves, including three downregulated [*SOD [Fe] 2, chloroplastic* (*FSD2*; Cs7g19240), *probable L-ASC peroxidase 6, chloroplastic/mitochondrial* (*APX6*; Cs3g19810) and *thioredoxin-like protein CDSP32*, *chloroplastic* (*CDSP32*, Cs3g26690)] and three upregulated (*PER47*, *SODCC.1*, and *SODCP*) genes in the antioxidant activity (GO:0016209), five downregulated genes in the sulfur (S) compound metabolic process (GO:0006790), four downregulated genes in the S amino acid biosynthetic process (GO:0000097), one upregulated *PCS2* (Cs5g29460) in the phytochelatin (PC) biosynthetic process (GO:0046938), two downregulated genes in the glutathione metabolism (cit00480), one upregulated *SULTR2;1* in S compound transport (GO:0072348), four downregulated genes [*ALDH4*, *inositol oxygenase 1* (*MIOX1*, Cs1g16030), *monodehydroascorbate reductase (MDAR) 4, peroxisomal* (*MDAR4*, Cs2g27950) and *APX6*] and one upregulated *GLCAK1* in the ASC and aldarate metabolism (cit00053), three downregulated genes and one upregulated gene in the VB_6_ metabolism, one downregulated *geranylgeranyl diphosphate reductase, chloroplastic* (*CHLP*; Cs5g10740) in the V_E_ biosynthetic process (GO:0010189), seven downregulated genes in the PPP pathway, and two downregulated other genes [*NADPH-dependent aldo-keto reductase, chloroplastic* (*AKR4C9*; orange1.1t03055), *2-alkenal reductase (NADP(+)-dependent)* (*DBR*; orange1.1t04575)] and one upregulated other gene (*GSTZ*) related to ROS and aldehyde detoxification. The antioxidant enzyme system is regarded as the first line of defense against oxidative stress in plant cells [80]. In this study, we identified four upregulated (*PER47*, *SODCC.1*, *SODCP* and *GSTZ*) and three downregulated (*APX6*, *FSD2* and *MDAR4*) antioxidant enzyme genes in low pH-treated leaves. This was consistent with higher SOD and peroxidase activities, but inconsistent with higher MDAR and APX activities and lower GST activity in low pH-treated sweet orange leaves [11]. The differences between the activities of enzymes and the expression abundances of related genes implied that PTMs and/or other factors had influence on enzyme activities.

S metabolism, a central pathway for the biosynthesis of S-containing compounds-namely PCs, Met, GSH, and cysteine, plays key roles in plant abiotic stress-tolerance. Here, all seven DEGs related to the S compound metabolic process, the S amino acid biosynthetic process, and the glutathione metabolism were downregulated in low pH-treated leaves, implying that the biosynthesis of S-containing compounds might be reduced in these leaves. Interestingly, the expression of *SULTR2;1* and *PCS2* were induced in low pH-treated leaves. *SULTR2;1* functions in root-to-shoot translocation of sulfate and plays a key role in the regulation of sulfate assimilation [81]. *SULTR2;1* antisense *Arabidopsis* plants displayed significant decreases of sulfate concentration in seeds and of cysteine and GSH concentrations in leaves and seeds, as well as a reduced trend in sulfate concentration in leaves [82]. The upregulation of *SULTR2;1* and *PCS2* could explain why low pH increased S, GSH, and GSSG concentrations in leaves, as well as S concentrations in stems and roots [4,11], and did not significantly affect PC (an increased trend) [22] and Met (Figure 4A) concentrations in leaves.

Both water-soluble (vitamins C and B) and lipid-soluble (vitamins E, A, and K) vitamins have strong antioxidant potential in plants. Here, low pH increased the concentrations of NA and PA, but decreased the concentrations of PN, ASC, and DHA [11] and might impair V_E_ biosynthesis, as indicated by the downregulated expression of *CHLP* in sweet orange leaves (Figure 4). MIOX functions in ASC biosynthesis. MDAR reduces monodehydroascorbate (MDHA) to ASC using NAD(P)H. APX catalyzes the conversion of H_2_O_2_ and ASC to H_2_O and MDHA. MDHA can be spontaneously disproportionated to ASC and dehydroascorbate (DHA). Low pH-induced downregulation of *MIOX* and upregulation of APX and MDAR activities implied that a reduction in ASC in low pH-treated leaves might be mainly caused by decreased biosynthesis and increased degradation. In addition, low pH increased the concentrations of the other antioxidants, such as Pro, Trp, and PG, but decreased the concentration of GABA in low pH-treated leaves (Figure 4).

In plants, SMs play an important role in the detoxification of ROS after the inactivation of antioxidant enzymes when exposed to severe/long-term stress [83]. Because of its function in providing the antioxidant cofactor NADPH for the biosynthesis of GSH, PPP is an important player in the detoxification of ROS in plants [84].

Aldo-keto reductase (AKR), alkenal reductase (AER), and ALDH play a role in oxidative stress tolerance through the detoxification of aldehydes derived from lipid peroxides in plants [85]. In addition to these detoxification enzymes, GSH conjugation to aldehydes leads to the detoxification of aldehydes [86]. This reaction can be catalyzed by GSTs. The downregulation of *ALDH4*, *AKR49* and *DBR* expression levels (Table S12) and reduction of GST activity [11] in low-pH treated leaves indicated that the detoxification of aldehydes was impaired in these leaves, as indicated by the increased concentration of methylglyoxal [11].

To conclude, low pH upregulated the expression of some antioxidant enzyme genes and increased the accumulation of some antioxidants (GSH, Trp, Pro, Met, NA, PA, and PG), but impaired PPP, V_E_, and SM biosynthesis and downregulated the expression of some ROS and aldehyde detoxifying enzymes (APX, AKR, and AER) and the concentrations of some antioxidants (ASC, PN, and GABA) in leaves. Generally viewed, the low pH disturbed the balance between production and detoxification of ROS and aldehydes, thus causing oxidative damage to leaves, as indicated by the decreased ratios of ASC/DHA and GSH/GSH, and increased concentrations of malondialdehyde and methylglyoxal [11].

## 4. Materials and Methods

### 4.1. Seedling Culture

Seedling cultures were performed according to Long et al. [4]. Seeds of sweet orange [*Citrus sinensis* (L.) Osbeck cv. Xuegan] were germinated in plastic trays filled with sand. Four weeks after germination, uniform seedlings were transplanted to 6 L pots containing sand (two seedlings per pot) and grown in the greenhouse at Fujian Agriculture and Forestry University, Fuzhou, China. Each treatment had 20 pots (a total of 40 seedlings) in a completely randomized design. Seven weeks after transporting, seedlings were supplied with freshly prepared nutrients solution containing macronutrients (in mM): KNO_3_, 2.5; KH_2_PO_4_, 0.5; Ca(NO_3_)_2_, 2.5 and MgSO_4_, 1; and micronutrients (in μM): Fe-EDTA, 20; H_3_BO_3_, 10; ZnSO_4_, 2; MnCl_2_, 2; CuSO_4_, 0.5 and (NH_4_)_6_Mo_7_O_24_, 0.065 at a pH 2.5 (low pH) and 6.0 (control, adjusted by 1 M HCl) until the dripping of nutrient solution from the hole at the bottom of pots. The recently fully expanded (~ 7-week-old) leaves were harvested nine months after pH treatments, immersed in liquid N_2_ immediately, then stored in −80 °C until extraction of RNA, OAs, and FAAs.

### 4.2. RNA Extraction, Library Construction, RNA-Seq, and Analysis

Equal amounts of sample from four seedlings (one seedling per pot) were mixed as a biological replicate. There were 2–3 biological replicates per treatment. Frozen leaves samples were sent to Biomarker Technologies (Beijing, China) for RNA extraction, library construction, sequencing, data processing and gene mapping. RNA was extracted with Biomarker Plant Total RNA Isolation Kit (RK02004, Beijing, China). RNA-Seq was performed at Biomarker Technologies with an Illumina HiSeq™ X-TEN platform (San Diego, CA, USA) using paired-end technology. After the removal of reads with low quality and reads containing ploy-N and/or adapter from raw data, high quality clean reads were mapped to *C. sinensis* reference sequences downloaded from the genome website (http://citrus.hzau.edu.cn/download.php, assessed on 1 January 2018) (*C. sinensis* v2.0) using TopHat2 [87]. Genes with a value of |log_2_(fold change)| > 1 and a false discovery rate (FDR) < 0.05 were assigned as DEGs. Gene functions were annotated according to KOG, Swiss-Prot, KEGG, GO, and National Centre for Biotechnology Information (NCBI) non-redundant protein sequences (NR) [11,88].

### 4.3. qRT-PCR Validation

Twenty-six DEGs were randomly selected for qRT-PCR validation. The sequences of the Reverse and Forward primers designed using Primer version 5.0 (Premier Biosoft International, Palo Alto, CA, USA) were summarized in Table S13. qRT-PCR was run in three biological replicates and two technical replicates using a CFX96 Touch^TM^ Deep Well Real-Time PCR Detection System (Bio-Rad, Hercules, CA, USA) [16]. *Actin, PRPF31*, and *β*-*tubulin* were selected as internal standards.

### 4.4. Assay of OAs and FAAs in Leaves

FAAs and OAs were assayed with an ultra-performance liquid chromatography-electrospray ionization-tandem mass spectrometry (UPLC-ESI-MS/MS) system (UPLC, Waters ACQUITY UPLC; MS, Applied Biosystems 4000 Triple Quadrupole) in Suzhou BioNovoGene Biomedical Tech Co., LTD (Suzhou, China) after 15 mg of freeze-dried leaves were extracted with 200 μL of 10% (*v*/*v*) formic acid dissolved in methanol solution: ddH_2_O (1:1 *v*/*v*) and 10 mg of freeze-dried leaves were extracted with 500 μL of 30% (*v*/*v*) methanol aqueous solution (containing 0.1% formic acid), respectively [55,89].

### 4.5. Statistical Analysis

Results represented the mean ± SE (*n* = 3). Comparison between two treatment means was performed in SigmaPlot 10 (Systat Software, Inc., San Jose, CA, USA) using a one-tailed *t*-test at *p* < 0.05.

## 5. Conclusions

Our results clearly demonstrated that low pH impaired photosynthetic pigment biosynthesis, light reaction, and carbon fixation in photosynthetic organisms, thereby lowering leaf pigment levels and photosynthesis. Low pH reduced carbon and carbohydrate metabolisms, OA biosynthesis, and energy (ATP) production in leaves. Low pH downregulated the biosynthesis of N compounds, proteins, and FAAs in leaves, which might be conducive to maintaining energy homeostasis during ATP deprivation. Low pH disturbed the balance between production and detoxification of ROS and aldehydes, thus causing oxidative damage to leaves. Low pH-treated leaves displayed some adaptive responses to Pi starvation, including Pi recycling, lipid remodeling (replacement of membrane phospholipids with non-P membrane lipids), and Pi transport, thus enhancing leaf acid-tolerance. These results corroborated the hypothesis that extensive reprogramming of gene expression and metabolites occurred in response to low pH in sweet orange leaves.

## Data Availability

RNA-Seq data are submitted to Gene Expression Omnibus (GEO) under accession no. GSE114369 (https://www.ncbi.nlm.nih.gov/search/all/?term=GSE114369). Data are archived in L.-S. Chen’s lab and available upon request.

## Supplementary Materials

The following supporting information can be downloaded at: https://www.mdpi.com/article/10.3390/ijms23105844/s1.

## Abbreviations

AAs	amino acids
A_CO2_	CO_2_ assimilation
AER	alkenal reductase
AKR	aldo-keto reductase
ALDH	aldehyde dehydrogenase
APX	ascorbate peroxidase
ASC	ascorbate
BP	biological process
CRTISO	carotenoid isomerase
CC	cellular component
DAG	diacylglycerol
DAMs	differentially abundant metabolites
DEGs	differentially expressed genes
DHA	dehydroascorbate
FAAs	free amino acids
GSH	reduced glutathione
GSSG	oxidized glutathione
GST	glutathione S-transferase
HFAs	hydroxy fatty acids
MDAR	monodehydroascorbate reductase
MDHA	monodehydroascorbate
MF	molecular function
NA	nicotinic acid
NAM	nicotinamide
OAA	oxaloacetate
OAs	organic acids
PA	pantothenic acid
PAP	purple acid phosphatase
PC	phytochelatin
PCK	phosphoenolpyruvate carboxykinase
PDCT	phosphatidylcholine:diacylglycerol cholinephosphotransferase
PEP	phosphoenolpyruvate
PEPC	phosphoenolpyruvate carboxylase
PETC	photosynthetic electron transport chain
PG	pyroglutamic acid
PK	pyruvate kinase
PN	pyridoxine
PPi	pyrophosphate
PPA	phenylpyruvic acid
PPiase	pyrophosphatase
PPP	pentose phosphate pathway
PTM	posttranslational modification
ROS	reactive oxygen species
RWC	relative water content
SAMDC	S-adenosylmethionine decarboxylase
SOD	superoxide dismutase
TFAAs	total free amino acids
TOAs	total organic acids
TNC	total nonstructural carbohydrates
