# Supplementary material for: Molecular and Physiological Responses of Citrus sinensis Leaves to Long-Term Low pH Revealed by RNA-Seq Integrated with Targeted Metabolomics
Source: Int J Mol Sci. 2022 May 23;23(10):5844. doi: 10.3390/ijms23105844 (PMC9142915; doi:10.3390/ijms23105844)
Supplement: Supplementary file 1 [file ijms-23-05844-s001.zip › ijms-1704493-supplementary/2022LaiIJMSSupplRevision0526/2022LaiSupplementary Tables0523.pdf]

Tables S1 and S7-S13

Table S1. Summary of the RNA-Seq data and genes mapped to the reference genome on pH 6.0-treated and of pH 2.5-treated leaves of *Citrus sinensis*. Q30 mean sequencing error rates lower than 1‰.

Treatment	Raw reads	Clean reads (%)	Clean bases (Gb)	Q30	Mapped reads	Uniquely mapped reads	Multiply mapped reads
pH 6.0	50,389,536	50,228,642 (99.68%)	7.45	94.80%	37,659,970 (74.98%)	36,187,884 (72.05%)	1,472,086 (2.93%)
pH 6.0	48,426,068	48,295,318 (99.73%)	7.17	94.71%	36,024,658 (74.59%)	34,458,981 (71.35%)	1,565,677 (3.24%)
pH 6.0	46,323,150	46,175,570 (99.68%)	6.86	94.73%	34,637,340 (75.01%)	33,275,554 (72.06%)	1,361,786 (2.95%)
pH 2.5	61,080,186	60,718,602 (99.41%)	9.00	94.86%	43,940,267 (72.37%)	42,317,244 (69.69%)	1,623,023 (2.67%)
pH 2.5	56,127,540	55,881,184 (99.56%)	8.29	94.88%	41,328,864 (73.96%)	39,673,313 (71.00%)	1,655,551 (2.96%)

Table S7. Differentially expressed genes (DEGs) related to photosynthesis in low pH-treated *Citrus sinensis* leaves

Accession No.	KEGG_annotation	Swissprot_annotation (NR_annotation)	Log_2(fold change)
Porphyrin and chlorophyll metabolism (cit00860, corrected P = 0.0005)			
Cs8g01360	K01885|8.0e-87|cit:102612397|K01885 glutamyl-tRNA synthetase [EC:6.1.1.17] | (RefSeq) glutamate--tRNA ligase, chloroplastic/mitochondrial	Glutamate--tRNA ligase, chloroplastic/mitochondrial OS=Arabidopsis thaliana OX=3702 GN=OVA3 PE=1 SV=1	-1.006
Cs4g07830	K02259|2.5e-61|cit:102630339|K02259 cytochrome c oxidase assembly protein subunit 15 | (RefSeq) cytochrome c oxidase assembly protein COX15	Cytochrome c oxidase assembly protein COX15 OS=Arabidopsis thaliana OX=3702 GN=COX15 PE=2 SV=1	-1.542
Cs3g16730	K02492|4.2e-206|cit:102624392|K02492 glutamyl-tRNA reductase [EC:1.2.1.70] | (RefSeq) glutamyl-tRNA reductase 1, chloroplastic	Glutamyl-tRNA reductase 1, chloroplastic OS=Cucumis sativus OX=3659 GN=HEMA1 PE=2 SV=1	-1.277
Cs5g10740	K10960|3.6e-141|cic:CICLE_v10020061mg|K10960 geranylgeranyl diphosphate/geranylgeranyl-bacteriochlorophyllide a reductase [EC:1.3.1.83 1.3.1.111] | (RefSeq) hypothetical protein	Geranylgeranyl diphosphate reductase, chloroplastic OS=Arabidopsis thaliana OX=3702 GN=CHLP PE=1 SV=1	-1.716
Cs2g05100	K03405|6.0e-194|cit:102620862|K03405 magnesium chelatase subunit I [EC:6.6.1.1] | (RefSeq) magnesium-chelatase subunit ChlI, chloroplastic	Magnesium-chelatase subunit ChlI, chloroplastic OS=Glycine max OX=3847 GN=CHLI PE=2 SV=1	-1.158
Cs9g04920	K19073|8.5e-235|cit:102611623|K19073 divinyl chlorophyllide a 8-vinyl-reductase [EC:1.3.1.75] | (RefSeq) divinyl chlorophyllide a 8-vinyl-reductase, chloroplastic	Divinyl chlorophyllide a 8-vinyl-reductase, chloroplastic OS=Cucumis sativus OX=3659 GN=DVR PE=1 SV=1	-1.624
Cs1g06850	K00218|5.4e-124|cit:102614959|K00218 protochlorophyllide reductase [EC:1.3.1.33] | (RefSeq) protochlorophyllide reductase, chloroplastic	Protochlorophyllide reductase, chloroplastic OS=Cucumis sativus OX=3659 GN=PORA PE=2 SV=1	-1.307
Cs3g19770	K21604|3.0e-71|cit:102608592|K21604 jasmonoyl-L-amino acid hydrolase [EC:3.5.1.127] | (RefSeq) IAA-amino acid hydrolase ILR1-like 6	IAA-amino acid hydrolase ILR1-like 6 OS=Oryza sativa subsp. japonica OX=39947 GN=ILL6 PE=2 SV=1	-1.120
Cs7g13850	K01749|3.5e-93|cic:CICLE_v10031858mg|K01749 hydroxymethylbilane synthase [EC:2.5.1.61] | (RefSeq) hypothetical protein	Porphobilinogen deaminase, chloroplastic OS=Pisum sativum OX=3888 GN=HEMC PE=1 SV=1	-1.414
Carotenoid biosynthesis (cit00906, corrected P = 0.0579)			
Cs4g14850	K06444|8.6e-73|cic:CICLE_v10008410mg|K06444 lycopene epsilon-cyclase [EC:5.5.1.18] | (RefSeq) hypothetical protein	Lycopene epsilon cyclase, chloroplastic OS=Arabidopsis thaliana OX=3702 GN=LUT2 PE=1 SV=2	-1.121
Cs5g26080	K09839|2.5e-150|cic:CICLE_v10019925mg|K09839 violaxanthin de-epoxidase [EC:1.23.5.1] | (RefSeq) hypothetical protein	Violaxanthin de-epoxidase, chloroplastic OS=Spinacia oleracea OX=3562 GN=VDE1 PE=1 SV=2	-1.246

Cs3g11180	K00514|7.0e-92|cit:102577969|K00514 zeta-carotene desaturase [EC:1.3.5.6] | (RefSeq) ZDS	Zeta-carotene desaturase, chloroplastic/chromoplastic OS=Capsicum annuum OX=4072 GN=ZDS PE=1 SV=1	-1.675
Cs3g23530	K09843|6.6e-61|cic:CICLE_v10001043mg|K09843 (+)-abscisic acid 8'-hydroxylase [EC:1.14.14.137] | (RefSeq) hypothetical protein	Abscisic acid 8'-hydroxylase 3 OS=Oryza sativa subsp. japonica OX=39947 GN=CYP707A7 PE=2 SV=1	-2.593
orange1.1t06069	K00514|1.4e-32|cit:102577969|K00514 zeta-carotene desaturase [EC:1.3.5.6] | (RefSeq) ZDS	Zeta-carotene desaturase, chloroplastic/chromoplastic OS=Tagetes erecta OX=13708 PE=2 SV=1	1.528
Photosynthesis (GO:0015979; adjusted P = 0.0030)			
Cs1g06850	K00218|5.4e-124|cit:102614959|K00218 protochlorophyllide reductase [EC:1.3.1.33] | (RefSeq) protochlorophyllide reductase, chloroplastic	Protochlorophyllide reductase, chloroplastic OS=Cucumis sativus OX=3659 GN=PORA PE=2 SV=1	-1.307
Cs1g09310	K02689|1.7e-31|aip:110272002|K02689 photosystem I P700 chlorophyll a apoprotein A1 | (RefSeq) uncharacterized protein LOC110272002	Photosystem I P700 chlorophyll a apoprotein A1 OS=Agrostis stolonifera OX=63632 GN=psaA PE=3 SV=1	-1.422
Cs1g18810	K02706|3.6e-50|ini:29082209|K02706 photosystem II P680 reaction center D2 protein [EC:1.10.3.9] | (RefSeq) psbD, IPN_cp071; photosystem II protein D2	Photosystem II D2 protein OS=Cuscuta exaltata OX=476139 GN=psbD PE=3 SV=1	-1.726
Cs1g23450	K02716|2.8e-165|cic:CICLE_v10026034mg|K02716 photosystem II oxygen-evolving enhancer protein 1 | (RefSeq) hypothetical protein	Oxygen-evolving enhancer protein 1, chloroplastic OS=Nicotiana tabacum OX=4097 GN=PSBO PE=2 SV=1	-1.142
Cs1g25510	K02641|5.8e-47|cic:CICLE_v10027108mg|K02641 ferredoxin--NADP+ reductase [EC:1.18.1.2] | (RefSeq) hypothetical protein	Ferredoxin--NADP reductase, leaf-type isozyme, chloroplastic OS=Nicotiana tabacum OX=4097 GN=PETH PE=2 SV=1	-1.068
Cs2g05100	K03405|6.0e-194|cit:102620862|K03405 magnesium chelatase subunit I [EC:6.6.1.1] | (RefSeq) magnesium-chelatase subunit ChlI, chloroplastic	Magnesium-chelatase subunit ChlI, chloroplastic OS=Glycine max OX=3847 GN=CHLI PE=2 SV=1	-1.158
Cs2g09070	K02703|1.7e-97|aly:32283147|K02703 photosystem II P680 reaction center D1 protein [EC:1.10.3.9] | (RefSeq) psbA, B9N47_pgp099; photosystem II protein D1	Photosystem II protein D1 OS=Anthoceros angustus OX=48387 GN=psbA PE=2 SV=1	-2.156
Cs2g15180	K03798|3.4e-271|cit:102623114|K03798 cell division protease FtsH [EC:3.4.24.-] | (RefSeq) ATP-dependent zinc metalloprotease FTSH 2, chloroplastic isoform X1	ATP-dependent zinc metalloprotease FTSH 2, chloroplastic OS=Arabidopsis thaliana OX=3702 GN=FTSH2 PE=1 SV=1	-1.619
Cs3g06200	K02706|1.3e-87|aly:32283168|K02706 photosystem II P680 reaction center D2 protein [EC:1.10.3.9] | (RefSeq) psbD, B9N47_pgp079; photosystem II protein D2	Photosystem II D2 protein OS=Acorus americanus OX=263995 GN=psbD PE=3 SV=1	-1.894
Cs3g27030	K03798|3.5e-297|cit:102607431|K03798 cell division protease FtsH [EC:3.4.24.-] | (RefSeq) ATP-dependent zinc metalloprotease FTSH, chloroplastic	ATP-dependent zinc metalloprotease FTSH 5, chloroplastic OS=Arabidopsis thaliana OX=3702 GN=FTSH5 PE=1 SV=1	-1.218

Cs4g07150	K08908|9.5e-112|cit:102607051|K08908 light-harvesting complex I chlorophyll a/b binding protein 2 | (RefSeq) photosystem I chlorophyll a/b-binding protein 6, chloroplastic isoform X1	Photosystem I chlorophyll a/b-binding protein 6, chloroplastic OS=Arabidopsis thaliana OX=3702 GN=LHCA6 PE=1 SV=1	-1.058
Cs5g33500	K00951|1.3e-88|cic:CICLE_v10018801mg|K00951 GTP pyrophosphokinase [EC:2.7.6.5] | (RefSeq) hypothetical protein	Putative GTP diphosphokinase RSH1, chloroplastic OS=Arabidopsis thaliana OX=3702 GN=RSH1 PE=1 SV=1	-1.345
Cs5g34250		Pentatricopeptide repeat-containing protein At2g15820, chloroplastic OS=Arabidopsis thaliana OX=3702 GN=OTP51 PE=2 SV=3	-1.618
Cs7g31190	K08915|5.5e-137|cic:CICLE_v10032377mg|K08915 light-harvesting complex II chlorophyll a/b binding protein 4 | (RefSeq) hypothetical protein	Chlorophyll a-b binding protein CP29.3, chloroplastic OS=Arabidopsis thaliana OX=3702 GN=LHCB4.3 PE=2 SV=1	-1.018
orange1.1t00477	K15111|4.9e-40|spen:107015750|K15111 solute carrier family 25 (mitochondrial S-adenosylmethionine transporter), member 26 | (RefSeq) LOW QUALITY PROTEIN: protein MITOFERRINLIKE 1, chloroplastic	Protein WHAT'S THIS FACTOR 1 homolog, chloroplastic OS=Arabidopsis thaliana OX=3702 GN=At4g01037 PE=3 SV=1	-1.774
orange1.1t01775	K03070|1.2e-82|cit:102630213|K03070 preprotein translocase subunit SecA [EC:7.4.2.8] | (RefSeq) protein translocase subunit SecA, chloroplastic	Protein translocase subunit SecA, chloroplastic OS=Spinacia oleracea OX=3562 GN=secA PE=1 SV=1	-1.357
Photosynthesis - antenna proteins (cit00196; corrected P = 0.3519)			
Cs7g31190	K08915|5.5e-137|cic:CICLE_v10032377mg|K08915 light-harvesting complex II chlorophyll a/b binding protein 4 | (RefSeq) hypothetical protein	Chlorophyll a-b binding protein CP29.3, chloroplastic OS=Arabidopsis thaliana OX=3702 GN=LHCB4.3 PE=2 SV=1	-1.018
Cs4g07150	K08908|9.5e-112|cit:102607051|K08908 light-harvesting complex I chlorophyll a/b binding protein 2 | (RefSeq) photosystem I chlorophyll a/b-binding protein 6, chloroplastic isoform X1	Photosystem I chlorophyll a/b-binding protein 6, chloroplastic OS=Arabidopsis thaliana OX=3702 GN=LHCA6 PE=1 SV=1	-1.058
Photosynthetic electron transport chain (GO:0009767; adjusted P = 0.0294)			
Cs1g18810	K02706|3.6e-50|ini:29082209|K02706 photosystem II P680 reaction center D2 protein [EC:1.10.3.9] | (RefSeq) psbD, IPN_cp071; photosystem II protein D2	Photosystem II D2 protein OS=Cuscuta exaltata OX=476139 GN=psbD PE=3 SV=1	-1.726
Cs1g25510	K02641|5.8e-47|cic:CICLE_v10027108mg|K02641 ferredoxin--NADP+ reductase [EC:1.18.1.2] | (RefSeq) hypothetical protein	Ferredoxin--NADP reductase, leaf-type isozyme, chloroplastic OS=Nicotiana tabacum OX=4097 GN=PETH PE=2 SV=1	-1.068

Cs2g09070	K02703|1.7e-97|aly:32283147|K02703 photosystem II P680 reaction center D1 protein [EC:1.10.3.9] | (RefSeq) psbA, B9N47_pgp099; photosystem II protein D1	Photosystem II protein D1 OS=Anthoceros angustus OX=48387 GN=psbA PE=2 SV=1	-2.156
Cs2g15180	K03798|3.4e-271|cit:102623114|K03798 cell division protease FtsH [EC:3.4.24.-] | (RefSeq) ATP-dependent zinc metalloprotease FTSH 2, chloroplastic isoform X1	ATP-dependent zinc metalloprotease FTSH 2, chloroplastic OS=Arabidopsis thaliana OX=3702 GN=FTSH2 PE=1 SV=1	-1.619
Cs3g06200	K02706|1.3e-87|aly:32283168|K02706 photosystem II P680 reaction center D2 protein [EC:1.10.3.9] | (RefSeq) psbD, B9N47_pgp079; photosystem II protein D2	Photosystem II D2 protein OS=Acorus americanus OX=263995 GN=psbD PE=3 SV=1	-1.894
Cs3g27030	K03798|3.5e-297|cit:102607431|K03798 cell division protease FtsH [EC:3.4.24.-] | (RefSeq) ATP-dependent zinc metalloprotease FTSH, chloroplastic	ATP-dependent zinc metalloprotease FTSH 5, chloroplastic OS=Arabidopsis thaliana OX=3702 GN=FTSH5 PE=1 SV=1	-1.218
Photosynthesis, light reaction (GO:0019684; adjusted P = 0.0074)			
Cs1g18810	K02706|3.6e-50|ini:29082209|K02706 photosystem II P680 reaction center D2 protein [EC:1.10.3.9] | (RefSeq) psbD, IPN_cp071; photosystem II protein D2	Photosystem II D2 protein OS=Cuscuta exaltata OX=476139 GN=psbD PE=3 SV=1	-1.726
Cs1g23450	K02716|2.8e-165|cic:CICLE_v10026034mg|K02716 photosystem II oxygen-evolving enhancer protein 1 | (RefSeq) hypothetical protein	Oxygen-evolving enhancer protein 1, chloroplastic OS=Nicotiana tabacum OX=4097 GN=PSBO PE=2 SV=1	-1.142
Cs1g25510	K02641|5.8e-47|cic:CICLE_v10027108mg|K02641 ferredoxin--NADP+ reductase [EC:1.18.1.2] | (RefSeq) hypothetical protein	Ferredoxin--NADP reductase, leaf-type isozyme, chloroplastic OS=Nicotiana tabacum OX=4097 GN=PETH PE=2 SV=1	-1.068
Cs2g09070	K02703|1.7e-97|aly:32283147|K02703 photosystem II P680 reaction center D1 protein [EC:1.10.3.9] | (RefSeq) psbA, B9N47_pgp099; photosystem II protein D1	Photosystem II protein D1 OS=Anthoceros angustus OX=48387 GN=psbA PE=2 SV=1	-2.156
Cs2g15180	K03798|3.4e-271|cit:102623114|K03798 cell division protease FtsH [EC:3.4.24.-] | (RefSeq) ATP-dependent zinc metalloprotease FTSH 2, chloroplastic isoform X1	ATP-dependent zinc metalloprotease FTSH 2, chloroplastic OS=Arabidopsis thaliana OX=3702 GN=FTSH2 PE=1 SV=1	-1.619
Cs3g06200	K02706|1.3e-87|aly:32283168|K02706 photosystem II P680 reaction center D2 protein [EC:1.10.3.9] | (RefSeq) psbD, B9N47_pgp079; photosystem II protein D2	Photosystem II D2 protein OS=Acorus americanus OX=263995 GN=psbD PE=3 SV=1	-1.894
Cs3g27030	K03798|3.5e-297|cit:102607431|K03798 cell division protease FtsH [EC:3.4.24.-] | (RefSeq) ATP-dependent zinc metalloprotease FTSH, chloroplastic	ATP-dependent zinc metalloprotease FTSH 5, chloroplastic OS=Arabidopsis thaliana OX=3702 GN=FTSH5 PE=1 SV=1	-1.218

Cs4g07150	K08908|9.5e-112|cit:102607051|K08908 light-harvesting complex I chlorophyll a/b binding protein 2 | (RefSeq) photosystem I chlorophyll a/b-binding protein 6, chloroplastic isoform X1	Photosystem I chlorophyll a/b-binding protein 6, chloroplastic OS=Arabidopsis thaliana OX=3702 GN=LHCA6 PE=1 SV=1	-1.058
Cs5g34250		Pentatricopeptide repeat-containing protein At2g15820, chloroplastic OS=Arabidopsis thaliana OX=3702 GN=OTP51 PE=2 SV=3	-1.618
Cs7g31190	K08915|5.5e-137|cic:CICLE_v10032377mg|K08915 light-harvesting complex II chlorophyll a/b binding protein 4 | (RefSeq) hypothetical protein	Chlorophyll a-b binding protein CP29.3, chloroplastic OS=Arabidopsis thaliana OX=3702 GN=LHCB4.3 PE=2 SV=1	-1.018
Carbon fixation in photosynthetic organisms (cit00710; corrected P < 0.0001)			
orange1.1t00226	K00615|1.8e-253|cic:CICLE_v10018991mg|K00615 transketolase [EC:2.2.1.1] | (RefSeq) hypothetical protein	Transketolase, chloroplastic OS=Zea mays OX=4577 PE=1 SV=1	-1.337
Cs3g10480	K01807|1.9e-150|cic:CICLE_v10002135mg|K01807 ribose 5-phosphate isomerase A [EC:5.3.1.6] | (RefSeq) hypothetical protein	Probable ribose-5-phosphate isomerase 3, chloroplastic OS=Arabidopsis thaliana OX=3702 GN=RPI3 PE=1 SV=1	-1.730
Cs5g10920	K01783|3.3e-19|adu:107472214|K01783 ribulose-phosphate 3-epimerase [EC:5.1.3.1] | (RefSeq) ribulose-phosphate 3-epimerase, chloroplastic	Ribulose-5-phosphate-3-epimerase, chloroplastic OS=Arabidopsis thaliana OX=3702 GN=RPE PE=2 SV=1	-1.001
Cs5g10730	K00026|2.1e-223|cit:102628532|K00026 malate dehydrogenase [EC:1.1.1.37] | (RefSeq) malate dehydrogenase, chloroplastic	Malate dehydrogenase, chloroplastic OS=Arabidopsis thaliana OX=3702 GN=At3g47520 PE=1 SV=1	-1.256
Cs3g08480	K00855|1.4e-97|cic:CICLE_v10001344mg|K00855 phosphoribulokinase [EC:2.7.1.19] | (RefSeq) hypothetical protein	Phosphoribulokinase, chloroplastic OS=Mesembryanthemum crystallinum OX=3544 PE=2 SV=1	-1.341
orange1.1t03280	K00927|9.7e-74|cic:CICLE_v10004891mg|K00927 phosphoglycerate kinase [EC:2.7.2.3] | (RefSeq) hypothetical protein	Phosphoglycerate kinase, chloroplastic OS=Nicotiana tabacum OX=4097 PE=2 SV=1	-1.999
Cs2g13700	K01807|3.2e-144|cit:102627714|K01807 ribose 5-phosphate isomerase A [EC:5.3.1.6] | (RefSeq) probable ribose-5-phosphate isomerase 2	Probable ribose-5-phosphate isomerase 2 OS=Arabidopsis thaliana OX=3702 GN=RPI2 PE=1 SV=1	-1.305
Cs6g07430	K03841|1.5e-138|cic:CICLE_v10011855mg|K03841 fructose-1,6-bisphosphatase I [EC:3.1.3.11] | (RefSeq) hypothetical protein	Fructose-1,6-bisphosphatase 1, chloroplastic OS=Arabidopsis thaliana OX=3702 GN=CFBP1 PE=1 SV=2	-1.619
Cs2g15520	K01595|6.0e-206|cit:102607204|K01595 phosphoenolpyruvate carboxylase [EC:4.1.1.31] | (RefSeq) phosphoenolpyruvate carboxylase 2	Phosphoenolpyruvate carboxylase 1 OS=Arabidopsis thaliana OX=3702 GN=PPC1 PE=1 SV=1	-2.477
Cs7g31640	K01100|1.2e-126|cic:CICLE_v10031765mg|K01100 sedoheptulose-bisphosphatase [EC:3.1.3.37] | (RefSeq) hypothetical protein	Sedoheptulose-1,7-bisphosphatase, chloroplastic OS=Triticum aestivum OX=4565 PE=2 SV=1	-1.175

Cs1g20920	K01610|1.9e-101|cit:102629129|K01610 phosphoenolpyruvate carboxykinase (ATP) [EC:4.1.1.49] | (RefSeq) phosphoenolpyruvate carboxykinase (ATP)-like	Phosphoenolpyruvate carboxykinase (ATP) 1 OS=Arabidopsis thaliana OX=3702 GN=PCK1 PE=1 SV=1	1.058
Cs7g25390	K00026|1.5e-53|cic:CICLE_v10008562mg|K00026 malate dehydrogenase [EC:1.1.1.37] | (RefSeq) hypothetical protein	Malate dehydrogenase, mitochondrial OS=Citrullus lanatus OX=3654 GN=MMDH PE=1 SV=1	-1.555
Stomatal movement (GO:0010118; adjusted P = 0.8090)			
Cs1g13200	K14558|9.9e-260|cit:102614962|K14558 periodic tryptophan protein 2 | (RefSeq) periodic tryptophan protein 2	Periodic tryptophan protein 2 OS=Arabidopsis thaliana OX=3702 GN=PWP2 PE=1 SV=1	-1.251
Cs5g04280	K14638|2.0e-181|cit:102607539|K14638 solute carrier family 15 (peptide/histidine transporter), member 3/4 | (RefSeq) protein NRT1/ PTR FAMILY 4.6 isoform X1	Protein NRT1/ PTR FAMILY 4.6 OS=Arabidopsis thaliana OX=3702 GN=NPF4.6 PE=1 SV=1	-1.838

Table S8. Differentially expressed genes (DEGs) related to carbohydrate and energy metabolisms in low pH-treated *Citrus sinensis* leaves

Accession No.	KEGG_annotation	Swissprot_annotation (NR_annotation)	Log_2(fold change)
	Carbon metabolism (cit01200; corrected P = 0.0008)		
Cs3g10480	K01807|1.9e-150|cic:CICLE_v10002135mg|K01807 ribose 5-phosphate isomerase A [EC:5.3.1.6] | (RefSeq) hypothetical protein	Probable ribose-5-phosphate isomerase 3, chloroplastic OS=Arabidopsis thaliana OX=3702 GN=RPI3 PE=1 SV=1	-1.730
Cs7g13170	K00627|2.3e-167|cit:102609118|K00627 pyruvate dehydrogenase E2 component (dihydrolipoamide acetyltransferase) [EC:2.3.1.12] | (RefSeq) dihydrolipoyllysine-residue acetyltransferase component 4 of pyruvate dehydrogenase complex, chloroplastic	Dihydrolipoyllysine-residue acetyltransferase component 4 of pyruvate dehydrogenase complex, chloroplastic OS=Arabidopsis thaliana OX=3702 GN=LTA2 PE=2 SV=1	-1.118
orange1.1t03280	K00927|9.7e-74|cic:CICLE_v10004891mg|K00927 phosphoglycerate kinase [EC:2.7.2.3] | (RefSeq) hypothetical protein	Phosphoglycerate kinase, chloroplastic OS=Nicotiana tabacum OX=4097 PE=2 SV=1	-1.999
Cs5g10920	K01783|3.3e-19|adu:107472214|K01783 ribulose-phosphate 3-epimerase [EC:5.1.3.1] | (RefSeq) ribulose-phosphate 3-epimerase, chloroplastic	Ribulose-5-phosphate-3-epimerase, chloroplastic OS=Arabidopsis thaliana OX=3702 GN=RPE PE=2 SV=1	-1.001
Cs7g31640	K01100|1.2e-126|cic:CICLE_v10031765mg|K01100 sedoheptulose-bisphosphatase [EC:3.1.3.37] | (RefSeq) hypothetical protein	Sedoheptulose-1,7-bisphosphatase, chloroplastic OS=Triticum aestivum OX=4565 PE=2 SV=1	-1.175
Cs7g25390	K00026|1.5e-53|cic:CICLE_v10008562mg|K00026 malate dehydrogenase [EC:1.1.1.37] | (RefSeq) hypothetical protein	Malate dehydrogenase, mitochondrial OS=Citrullus lanatus OX=3654 GN=MMDH PE=1 SV=1	-1.555
Cs2g02890	K00605|5.7e-118|cic:CICLE_v10015433mg|K00605 aminomethyltransferase [EC:2.1.2.10] | (RefSeq) hypothetical protein	Aminomethyltransferase, mitochondrial OS=Solanum tuberosum OX=4113 GN=GDCST PE=2 SV=1	-1.910
Cs4g18520	K00281|5.5e-312|cit:102627653|K00281 glycine dehydrogenase [EC:1.4.4.2] | (RefSeq) glycine dehydrogenase (decarboxylating), mitochondrial	Glycine dehydrogenase (decarboxylating) A, mitochondrial OS=Flaveria pringlei OX=4226 GN=GDCSPA PE=2 SV=1	-1.292
Cs9g02530	K01913|1.0e-266|cic:CICLE_v10004645mg|K01913 acetate/butyrate---CoA ligase [EC:6.2.1.1 6.2.1.2] | (RefSeq) hypothetical protein	Acetate/butyrate--CoA ligase AAE7, peroxisomal OS=Arabidopsis thaliana OX=3702 GN=AAE7 PE=1 SV=1	-1.210
Cs7g14430	K00121|1.7e-106|cit:102622247|K00121 S-(hydroxymethyl)glutathione dehydrogenase / alcohol dehydrogenase [EC:1.1.1.284 1.1.1.1] | (RefSeq) alcohol dehydrogenase-like 2 isoform X1	CYP enzymes assisting alcohol dehydrogenase OS=Catharanthus roseus OX=4058 GN=CYPADH PE=1 SV=1	1.605
Cs6g07430	K03841|1.5e-138|cic:CICLE_v10011855mg|K03841 fructose-1,6-bisphosphatase I [EC:3.1.3.11] | (RefSeq) hypothetical protein	Fructose-1,6-bisphosphatase 1, chloroplastic OS=Arabidopsis thaliana OX=3702 GN=CFBP1 PE=1 SV=2	-1.619

Cs2g15520	K01595|6.0e-206|cit:102607204|K01595 phosphoenolpyruvate carboxylase [EC:4.1.1.31] | (RefSeq) phosphoenolpyruvate carboxylase 2	Phosphoenolpyruvate carboxylase 1 OS=Arabidopsis thaliana OX=3702 GN=PPC1 PE=1 SV=1	-2.477
Cs9g01670	K00382|3.8e-234|cit:102627107|K00382 dihydrolipoamide dehydrogenase [EC:1.8.1.4] | (RefSeq) dihydrolipoyl dehydrogenase 1, mitochondrial isoform X1	Dihydrolipoyl dehydrogenase 1, mitochondrial OS=Arabidopsis thaliana OX=3702 GN=LPD1 PE=1 SV=2	-2.754
Cs3g11490	K01738|3.2e-72|cic:CICLE_v10001793mg|K01738 cysteine synthase [EC:2.5.1.47] | (RefSeq) hypothetical protein	Cysteine synthase, chloroplastic/chromoplastic OS=Solanum tuberosum OX=4113 PE=2 SV=1	-1.300
Cs7g11110	K00036|2.2e-114|cit:102624333|K00036 glucose-6-phosphate 1-dehydrogenase [EC:1.1.1.49 1.1.1.363] | (RefSeq) glucose-6-phosphate 1-dehydrogenase, chloroplastic isoform X1	Glucose-6-phosphate 1-dehydrogenase, chloroplastic OS=Nicotiana tabacum OX=4097 PE=2 SV=1	-1.151
Cs2g13700	K01807|3.2e-144|cit:102627714|K01807 ribose 5-phosphate isomerase A [EC:5.3.1.6] | (RefSeq) probable ribose-5-phosphate isomerase 2	Probable ribose-5-phosphate isomerase 2 OS=Arabidopsis thaliana OX=3702 GN=RPI2 PE=1 SV=1	-1.305
Cs5g10730	K00026|2.1e-223|cit:102628532|K00026 malate dehydrogenase [EC:1.1.1.37] | (RefSeq) malate dehydrogenase, chloroplastic	Malate dehydrogenase, chloroplastic OS=Arabidopsis thaliana OX=3702 GN=At3g47520 PE=1 SV=1	-1.256
Cs3g08480	K00855|1.4e-97|cic:CICLE_v10001344mg|K00855 phosphoribulokinase [EC:2.7.1.19] | (RefSeq) hypothetical protein	Phosphoribulokinase, chloroplastic OS=Mesembryanthemum crystallinum OX=3544 PE=2 SV=1	-1.341
Cs1g20920	K01610|1.9e-101|cit:102629129|K01610 phosphoenolpyruvate carboxykinase (ATP) [EC:4.1.1.49] | (RefSeq) phosphoenolpyruvate carboxykinase (ATP)-like	Phosphoenolpyruvate carboxykinase (ATP) 1 OS=Arabidopsis thaliana OX=3702 GN=PCK1 PE=1 SV=1	1.058
orange1.1t00226	K00615|1.8e-253|cic:CICLE_v10018991mg|K00615 transketolase [EC:2.2.1.1] | (RefSeq) hypothetical protein	Transketolase, chloroplastic OS=Zea mays OX=4577 PE=1 SV=1	-1.337
Starch and sucrose metabolism (cit00500; corrected P = 0.6822)			
Cs9g06360	K00975|2.1e-69|cic:CICLE_v10004731mg|K00975 glucose-1-phosphate adenylyltransferase [EC:2.7.7.27] | (RefSeq) hypothetical protein	Glucose-1-phosphate adenylyltransferase large subunit 1, chloroplastic OS=Arabidopsis thaliana OX=3702 GN=ADG2 PE=1 SV=3	-1.206
orange1.1t05474	K01835|3.9e-26|cic:CICLE_v10014721mg|K01835 phosphoglucomutase [EC:5.4.2.2] | (RefSeq) hypothetical protein	Phosphoglucomutase, cytoplasmic OS=Mesembryanthemum crystallinum OX=3544 GN=PGM1 PE=2 SV=1	-1.607

Cs6g21370	K00703|1.0e-258|cit:102614846|K00703 starch synthase [EC:2.4.1.21] | (RefSeq) granule-bound starch synthase 2, chloroplastic/amyloplastic	Granule-bound starch synthase 2, chloroplastic/amyloplastic OS=Solanum tuberosum OX=4113 GN=SS2 PE=1 SV=3	-1.754
Cs2g17050	K01835|1.1e-16|cic:CICLE_v10014721mg|K01835 phosphoglucomutase [EC:5.4.2.2] | (RefSeq) hypothetical protein	Probable phosphoglucomutase, cytoplasmic 1 OS=Arabidopsis thaliana OX=3702 GN=At1g23190 PE=2 SV=2	-1.761
Cs7g01350	K01188|1.0e-74|cic:CICLE_v10031209mg|K01188 beta-glucosidase [EC:3.2.1.21] | (RefSeq) hypothetical protein	Beta-glucosidase 13 OS=Oryza sativa subsp. japonica OX=39947 GN=BGLU13 PE=2 SV=2	-1.095
Pentose phosphate pathway (cit00030; corrected P = 0.0029)			
orange1.1t00226	K00615|1.8e-253|cic:CICLE_v10018991mg|K00615 transketolase [EC:2.2.1.1] | (RefSeq) hypothetical protein	Transketolase, chloroplastic OS=Zea mays OX=4577 PE=1 SV=1	-1.337
Cs3g10480	K01807|1.9e-150|cic:CICLE_v10002135mg|K01807 ribose 5-phosphate isomerase A [EC:5.3.1.6] | (RefSeq) hypothetical protein	Probable ribose-5-phosphate isomerase 3, chloroplastic OS=Arabidopsis thaliana OX=3702 GN=RPI3 PE=1 SV=1	-1.730
Cs7g11110	K00036|2.2e-114|cit:102624333|K00036 glucose-6-phosphate 1-dehydrogenase [EC:1.1.1.49 1.1.1.363] | (RefSeq) glucose-6-phosphate 1-dehydrogenase, chloroplastic isoform X1	Glucose-6-phosphate 1-dehydrogenase, chloroplastic OS=Nicotiana tabacum OX=4097 PE=2 SV=1	-1.151
Cs5g10920	K01783|3.3e-19|adu:107472214|K01783 ribulose-phosphate 3-epimerase [EC:5.1.3.1] | (RefSeq) ribulose-phosphate 3-epimerase, chloroplastic	Ribulose-5-phosphate-3-epimerase, chloroplastic OS=Arabidopsis thaliana OX=3702 GN=RPE PE=2 SV=1	-1.001
Cs2g13700	K01807|3.2e-144|cit:102627714|K01807 ribose 5-phosphate isomerase A [EC:5.3.1.6] | (RefSeq) probable ribose-5-phosphate isomerase 2	Probable ribose-5-phosphate isomerase 2 OS=Arabidopsis thaliana OX=3702 GN=RPI2 PE=1 SV=1	-1.305
Cs6g07430	K03841|1.5e-138|cic:CICLE_v10011855mg|K03841 fructose-1,6-bisphosphatase I [EC:3.1.3.11] | (RefSeq) hypothetical protein	Fructose-1,6-bisphosphatase 1, chloroplastic OS=Arabidopsis thaliana OX=3702 GN=CFBP1 PE=1 SV=2	-1.619
orange1.1t05474	K01835|3.9e-26|cic:CICLE_v10014721mg|K01835 phosphoglucomutase [EC:5.4.2.2] | (RefSeq) hypothetical protein	Phosphoglucomutase, cytoplasmic OS=Mesembryanthemum crystallinum OX=3544 GN=PGM1 PE=2 SV=1	-1.607
Cs2g17050	K01835|1.1e-16|cic:CICLE_v10014721mg|K01835 phosphoglucomutase [EC:5.4.2.2] | (RefSeq) hypothetical protein	Probable phosphoglucomutase, cytoplasmic 1 OS=Arabidopsis thaliana OX=3702 GN=At1g23190 PE=2 SV=2	-1.761
Glycolysis / gluconeogenesis (cit00010; corrected P = 0.0148)			

orange1.1t03055	K00002 | 1.2e-61 | cic:CICLE_v10012243mg | K00002 alcohol dehydrogenase (NADP+) [EC:1.1.1.2] | (RefSeq) hypothetical protein	NADPH-dependent aldo-keto reductase, chloroplastic OS=Arabidopsis thaliana OX=3702 GN=AKR4C9 PE=1 SV=1	-1.133
Cs7g13170	K00627 | 2.3e-167 | cit:102609118 | K00627 pyruvate dehydrogenase E2 component (dihydrolipoamide acetyltransferase) [EC:2.3.1.12] | (RefSeq) dihydrolipoyllysine-residue acetyltransferase component 4 of pyruvate dehydrogenase complex, chloroplastic	Dihydrolipoyllysine-residue acetyltransferase component 4 of pyruvate dehydrogenase complex, chloroplastic OS=Arabidopsis thaliana OX=3702 GN=LTA2 PE=2 SV=1	-1.118
orange1.1t03280	K00927 | 9.7e-74 | cic:CICLE_v10004891mg | K00927 phosphoglycerate kinase [EC:2.7.2.3] | (RefSeq) hypothetical protein	Phosphoglycerate kinase, chloroplastic OS=Nicotiana tabacum OX=4097 PE=2 SV=1	-1.999
Cs3g19820	K00128 | 2.9e-44 | cit:102611460 | K00128 aldehyde dehydrogenase (NAD+) [EC:1.2.1.3] | (RefSeq) aldehyde dehydrogenase family 3 member H1	Aldehyde dehydrogenase family 3 member H1 OS=Arabidopsis thaliana OX=3702 GN=ALDH3H1 PE=1 SV=2	-1.055
Cs9g01670	K00382 | 3.8e-234 | cit:102627107 | K00382 dihydrolipoamide dehydrogenase [EC:1.8.1.4] | (RefSeq) dihydrolipoyl dehydrogenase 1, mitochondrial isoform X1	Dihydrolipoyl dehydrogenase 1, mitochondrial OS=Arabidopsis thaliana OX=3702 GN=LPD1 PE=1 SV=2	-2.754
Cs9g02530	K01913 | 1.0e-266 | cic:CICLE_v10004645mg | K01913 acetate/butyrate---CoA ligase [EC:6.2.1.1 6.2.1.2] | (RefSeq) hypothetical protein	Acetate/butyrate--CoA ligase AAE7, peroxisomal OS=Arabidopsis thaliana OX=3702 GN=AAE7 PE=1 SV=1	-1.210
Cs6g07430	K03841 | 1.5e-138 | cic:CICLE_v10011855mg | K03841 fructose-1,6-bisphosphatase I [EC:3.1.3.11] | (RefSeq) hypothetical protein	Fructose-1,6-bisphosphatase 1, chloroplastic OS=Arabidopsis thaliana OX=3702 GN=CFBP1 PE=1 SV=2	-1.619
orange1.1t05474	K01835 | 3.9e-26 | cic:CICLE_v10014721mg | K01835 phosphoglucomutase [EC:5.4.2.2] | (RefSeq) hypothetical protein	Phosphoglucomutase, cytoplasmic OS=Mesembryanthemum crystallinum OX=3544 GN=PGM1 PE=2 SV=1	-1.607
Cs7g14430	K00121 | 1.7e-106 | cit:102622247 | K00121 S-(hydroxymethyl)glutathione dehydrogenase / alcohol dehydrogenase [EC:1.1.1.284 1.1.1.1] | (RefSeq) alcohol dehydrogenase-like 2 isoform X1	CYP enzymes assisting alcohol dehydrogenase OS=Catharanthus roseus OX=4058 GN=CYPADH PE=1 SV=1	1.605
Cs1g20920	K01610 | 1.9e-101 | cit:102629129 | K01610 phosphoenolpyruvate carboxykinase (ATP) [EC:4.1.1.49] | (RefSeq) phosphoenolpyruvate carboxykinase (ATP)-like	Phosphoenolpyruvate carboxykinase (ATP) 1 OS=Arabidopsis thaliana OX=3702 GN=PCK1 PE=1 SV=1	1.058
Cs2g17050	K01835 | 1.1e-16 | cic:CICLE_v10014721mg | K01835 phosphoglucomutase [EC:5.4.2.2] | (RefSeq) hypothetical protein	Probable phosphoglucomutase, cytoplasmic 1 OS=Arabidopsis thaliana OX=3702 GN=At1g23190 PE=2 SV=2	-1.761

Pyruvate metabolism (cit00620; corrected P = 0.0197)			
Cs7g13170	K00627|2.3e-167|cit:102609118|K00627 pyruvate dehydrogenase E2 component (dihydrolipoamide acetyltransferase) [EC:2.3.1.12] | (RefSeq) dihydrolipoyllysine-residue acetyltransferase component 4 of pyruvate dehydrogenase complex, chloroplastic	Dihydrolipoyllysine-residue acetyltransferase component 4 of pyruvate dehydrogenase complex, chloroplastic OS=Arabidopsis thaliana OX=3702 GN=LTA2 PE=2 SV=1	-1.118
Cs7g25390	K00026|1.5e-53|cic:CICLE_v10008562mg|K00026 malate dehydrogenase [EC:1.1.1.37] | (RefSeq) hypothetical protein	Malate dehydrogenase, mitochondrial OS=Citrullus lanatus OX=3654 GN=MMDH PE=1 SV=1	-1.555
Cs3g19820	K00128|2.9e-44|cit:102611460|K00128 aldehyde dehydrogenase (NAD+) [EC:1.2.1.3] | (RefSeq) aldehyde dehydrogenase family 3 member H1	Aldehyde dehydrogenase family 3 member H1 OS=Arabidopsis thaliana OX=3702 GN=ALDH3H1 PE=1 SV=2	-1.055
Cs9g02530	K01913|1.0e-266|cic:CICLE_v10004645mg|K01913 acetate/butyrate---CoA ligase [EC:6.2.1.1 6.2.1.2] | (RefSeq) hypothetical protein	Acetate/butyrate--CoA ligase AAE7, peroxisomal OS=Arabidopsis thaliana OX=3702 GN=AAE7 PE=1 SV=1	-1.210
Cs2g15520	K01595|6.0e-206|cit:102607204|K01595 phosphoenolpyruvate carboxylase [EC:4.1.1.31] | (RefSeq) phosphoenolpyruvate carboxylase 2	Phosphoenolpyruvate carboxylase 1 OS=Arabidopsis thaliana OX=3702 GN=PPC1 PE=1 SV=1	-2.477
Cs9g01670	K00382|3.8e-234|cit:102627107|K00382 dihydrolipoamide dehydrogenase [EC:1.8.1.4] | (RefSeq) dihydrolipoyl dehydrogenase 1, mitochondrial isoform X1	Dihydrolipoyl dehydrogenase 1, mitochondrial OS=Arabidopsis thaliana OX=3702 GN=LPD1 PE=1 SV=2	-2.754
Cs5g10730	K00026|2.1e-223|cit:102628532|K00026 malate dehydrogenase [EC:1.1.1.37] | (RefSeq) malate dehydrogenase, chloroplastic	Malate dehydrogenase, chloroplastic OS=Arabidopsis thaliana OX=3702 GN=At3g47520 PE=1 SV=1	-1.256
Cs1g20920	K01610|1.9e-101|cit:102629129|K01610 phosphoenolpyruvate carboxykinase (ATP) [EC:4.1.1.49] | (RefSeq) phosphoenolpyruvate carboxykinase (ATP)-like	Phosphoenolpyruvate carboxykinase (ATP) 1 OS=Arabidopsis thaliana OX=3702 GN=PCK1 PE=1 SV=1	1.058
Glyoxylate and dicarboxylate metabolism (cit00630; corrected P = 0.0213)			
Cs7g25390	K00026|1.5e-53|cic:CICLE_v10008562mg|K00026 malate dehydrogenase [EC:1.1.1.37] | (RefSeq) hypothetical protein	Malate dehydrogenase, mitochondrial OS=Citrullus lanatus OX=3654 GN=MMDH PE=1 SV=1	-1.555
Cs2g02890	K00605|5.7e-118|cic:CICLE_v10015433mg|K00605 aminomethyltransferase [EC:2.1.2.10] | (RefSeq) hypothetical protein	Aminomethyltransferase, mitochondrial OS=Solanum tuberosum OX=4113 GN=GDCST PE=2 SV=1	-1.910
Cs4g18520	K00281|5.5e-312|cit:102627653|K00281 glycine dehydrogenase [EC:1.4.4.2] | (RefSeq) glycine dehydrogenase (decarboxylating), mitochondrial	Glycine dehydrogenase (decarboxylating) A, mitochondrial OS=Flaveria pringlei OX=4226 GN=GDCSPA PE=2 SV=1	-1.292

Cs9g02530	K01913|1.0e-266|cic:CICLE_v10004645mg|K01913 acetate/butyrate---CoA ligase [EC:6.2.1.1 6.2.1.2] | (RefSeq) hypothetical protein	Acetate/butyrate--CoA ligase AAE7, peroxisomal OS=Arabidopsis thaliana OX=3702 GN=AAE7 PE=1 SV=1	-1.210
Cs9g01670	K00382|3.8e-234|cit:102627107|K00382 dihydrolipoamide dehydrogenase [EC:1.8.1.4] | (RefSeq) dihydrolipoyl dehydrogenase 1, mitochondrial isoform X1	Dihydrolipoyl dehydrogenase 1, mitochondrial OS=Arabidopsis thaliana OX=3702 GN=LPD1 PE=1 SV=2	-2.754
Cs9g05680	K01915|7.0e-71|cic:CICLE_v10005007mg|K01915 glutamine synthetase [EC:6.3.1.2] | (RefSeq) hypothetical protein	Glutamine synthetase, chloroplastic OS=Daucus carota OX=4039 GN=GLN2 PE=2 SV=1	-1.445
Cs5g10730	K00026|2.1e-223|cit:102628532|K00026 malate dehydrogenase [EC:1.1.1.37] | (RefSeq) malate dehydrogenase, chloroplastic	Malate dehydrogenase, chloroplastic OS=Arabidopsis thaliana OX=3702 GN=At3g47520 PE=1 SV=1	-1.256
Citrate cycle (TCA cycle) (cit00020; corrected P = 0.0681)			
Cs7g25390	K00026|1.5e-53|cic:CICLE_v10008562mg|K00026 malate dehydrogenase [EC:1.1.1.37] | (RefSeq) hypothetical protein	Malate dehydrogenase, mitochondrial OS=Citrullus lanatus OX=3654 GN=MMDH PE=1 SV=1	-1.555
Cs7g13170	K00627|2.3e-167|cit:102609118|K00627 pyruvate dehydrogenase E2 component (dihydrolipoamide acetyltransferase) [EC:2.3.1.12] | (RefSeq) dihydrolipoyllysine-residue acetyltransferase component 4 of pyruvate dehydrogenase complex, chloroplastic	Dihydrolipoyllysine-residue acetyltransferase component 4 of pyruvate dehydrogenase complex, chloroplastic OS=Arabidopsis thaliana OX=3702 GN=LTA2 PE=2 SV=1	-1.118
Cs5g10730	K00026|2.1e-223|cit:102628532|K00026 malate dehydrogenase [EC:1.1.1.37] | (RefSeq) malate dehydrogenase, chloroplastic	Malate dehydrogenase, chloroplastic OS=Arabidopsis thaliana OX=3702 GN=At3g47520 PE=1 SV=1	-1.256
Cs1g20920	K01610|1.9e-101|cit:102629129|K01610 phosphoenolpyruvate carboxykinase (ATP) [EC:4.1.1.49] | (RefSeq) phosphoenolpyruvate carboxykinase (ATP)-like	Phosphoenolpyruvate carboxykinase (ATP) 1 OS=Arabidopsis thaliana OX=3702 GN=PCK1 PE=1 SV=1	1.058
Cs9g01670	K00382|3.8e-234|cit:102627107|K00382 dihydrolipoamide dehydrogenase [EC:1.8.1.4] | (RefSeq) dihydrolipoyl dehydrogenase 1, mitochondrial isoform X1	Dihydrolipoyl dehydrogenase 1, mitochondrial OS=Arabidopsis thaliana OX=3702 GN=LPD1 PE=1 SV=2	-2.754
Oxidative phosphorylation (cit00190; corrected P = 0.6344)			
Cs5g16400	K03934|1.5e-188|cit:102610171|K03934 NADH dehydrogenase (ubiquinone) Fe-S protein 1 [EC:7.1.1.2] | (RefSeq) NADH dehydrogenase [ubiquinone] iron-sulfur protein 1, mitochondrial	NADH dehydrogenase [ubiquinone] iron-sulfur protein 1, mitochondrial OS=Solanum tuberosum OX=4113 PE=2 SV=1	-1.047
Cs2g03080	K02115|4.3e-195|cit:102629991|K02115 F-type H+-transporting ATPase subunit gamma | (RefSeq) ATP synthase gamma chain, chloroplastic	ATP synthase gamma chain, chloroplastic OS=Nicotiana tabacum OX=4097 GN=ATPC PE=1 SV=1	-1.309

Cs7g07300	K01535|0.0e+00|cit:102629401|K01535 H+-transporting ATPase [EC:7.1.2.1] | (RefSeq) plasma membrane ATPase-like	Plasma membrane ATPase OS=Oryza sativa subsp. japonica OX=39947 GN=Os04g0656100 PE=2 SV=1	-1.156
Cs6g05740	K01507|2.3e-39|cic:CICLE_v10012723mg|K01507 inorganic pyrophosphatase [EC:3.6.1.1] | (RefSeq) hypothetical protein	Soluble inorganic pyrophosphatase 4 OS=Arabidopsis thaliana OX=3702 GN=PPA4 PE=1 SV=1	1.164
Cs4g07830	K02259|2.5e-61|cit:102630339|K02259 cytochrome c oxidase assembly protein subunit 15 | (RefSeq) cytochrome c oxidase assembly protein COX15	Cytochrome c oxidase assembly protein COX15 OS=Arabidopsis thaliana OX=3702 GN=COX15 PE=2 SV=1	-1.542
ATP biosynthetic process (GO:0006754; adjusted P = 0.8922)			
Cs2g03080	K02115|4.3e-195|cit:102629991|K02115 F-type H+-transporting ATPase subunit gamma | (RefSeq) ATP synthase gamma chain, chloroplastic	ATP synthase gamma chain, chloroplastic OS=Nicotiana tabacum OX=4097 GN=ATPC PE=1 SV=1	-1.309
ATP generation from ADP (GO:0006757; adjusted P = 0.9342)			
orange1.1t03280	K00927|9.7e-74|cic:CICLE_v10004891mg|K00927 phosphoglycerate kinase [EC:2.7.2.3] | (RefSeq) hypothetical protein	Phosphoglycerate kinase, chloroplastic OS=Nicotiana tabacum OX=4097 PE=2 SV=1	-1.999
Generation of precursor metabolites and energy (GO:0006091; adjusted P = 0.0185)			
Cs1g18810	K02706|3.6e-50|ini:29082209|K02706 photosystem II P680 reaction center D2 protein [EC:1.10.3.9] | (RefSeq) psbD, IPN_cp071; photosystem II protein D2	Photosystem II D2 protein OS=Cuscuta exaltata OX=476139 GN=psbD PE=3 SV=1	-1.726
Cs1g23450	K02716|2.8e-165|cic:CICLE_v10026034mg|K02716 photosystem II oxygen-evolving enhancer protein 1 | (RefSeq) hypothetical protein	Oxygen-evolving enhancer protein 1, chloroplastic OS=Nicotiana tabacum OX=4097 GN=PSBO PE=2 SV=1	-1.142
Cs1g25510	K02641|5.8e-47|cic:CICLE_v10027108mg|K02641 ferredoxin--NADP+ reductase [EC:1.18.1.2] | (RefSeq) hypothetical protein	Ferredoxin--NADP reductase, leaf-type isozyme, chloroplastic OS=Nicotiana tabacum OX=4097 GN=PETH PE=2 SV=1	-1.068
Cs2g09070	K02703|1.7e-97|aly:32283147|K02703 photosystem II P680 reaction center D1 protein [EC:1.10.3.9] | (RefSeq) psbA, B9N47_pgp099; photosystem II protein D1	Photosystem II protein D1 OS=Anthoceros angustus OX=48387 GN=psbA PE=2 SV=1	-2.156
Cs2g15180	K03798|3.4e-271|cit:102623114|K03798 cell division protease FtsH [EC:3.4.24.-] | (RefSeq) ATP-dependent zinc metalloprotease FTSH 2, chloroplastic isoform X1	ATP-dependent zinc metalloprotease FTSH 2, chloroplastic OS=Arabidopsis thaliana OX=3702 GN=FTSH2 PE=1 SV=1	-1.619

Cs2g15520	K01595|6.0e-206|cit:102607204|K01595 phosphoenolpyruvate carboxylase [EC:4.1.1.31] | (RefSeq) phosphoenolpyruvate carboxylase 2	Phosphoenolpyruvate carboxylase 1 OS=Arabidopsis thaliana OX=3702 GN=PPC1 PE=1 SV=1	-2.477
Cs3g06200	K02706|1.3e-87|aly:32283168|K02706 photosystem II P680 reaction center D2 protein [EC:1.10.3.9] | (RefSeq) psbD, B9N47_pgp079; photosystem II protein D2	Photosystem II D2 protein OS=Acorus americanus OX=263995 GN=psbD PE=3 SV=1	-1.894
Cs3g27030	K03798|3.5e-297|cit:102607431|K03798 cell division protease FtsH [EC:3.4.24.-] | (RefSeq) ATP-dependent zinc metalloprotease FTSH, chloroplastic	ATP-dependent zinc metalloprotease FTSH 5, chloroplastic OS=Arabidopsis thaliana OX=3702 GN=FTSH5 PE=1 SV=1	-1.218
Cs4g07150	K08908|9.5e-112|cit:102607051|K08908 light-harvesting complex I chlorophyll a/b binding protein 2 | (RefSeq) photosystem I chlorophyll a/b-binding protein 6, chloroplastic isoform X1	Photosystem I chlorophyll a/b-binding protein 6, chloroplastic OS=Arabidopsis thaliana OX=3702 GN=LHCA6 PE=1 SV=1	-1.058
Cs5g10730	K00026|2.1e-223|cit:102628532|K00026 malate dehydrogenase [EC:1.1.1.37] | (RefSeq) malate dehydrogenase, chloroplastic	Malate dehydrogenase, chloroplastic OS=Arabidopsis thaliana OX=3702 GN=At3g47520 PE=1 SV=1	-1.256
Cs5g16400	K03934|1.5e-188|cit:102610171|K03934 NADH dehydrogenase (ubiquinone) Fe-S protein 1 [EC:7.1.1.2] | (RefSeq) NADH dehydrogenase [ubiquinone] iron-sulfur protein 1, mitochondrial	NADH dehydrogenase [ubiquinone] iron-sulfur protein 1, mitochondrial OS=Solanum tuberosum OX=4113 PE=2 SV=1	-1.047
Cs5g34250		Pentatricopeptide repeat-containing protein At2g15820, chloroplastic OS=Arabidopsis thaliana OX=3702 GN=OTP51 PE=2 SV=3	-1.618
Cs7g25390	K00026|1.5e-53|cic:CICLE_v10008562mg|K00026 malate dehydrogenase [EC:1.1.1.37] | (RefSeq) hypothetical protein	Malate dehydrogenase, mitochondrial OS=Citrullus lanatus OX=3654 GN=MMDH PE=1 SV=1	-1.555
Cs7g31190	K08915|5.5e-137|cic:CICLE_v10032377mg|K08915 light-harvesting complex II chlorophyll a/b binding protein 4 | (RefSeq) hypothetical protein	Chlorophyll a-b binding protein CP29.3, chloroplastic OS=Arabidopsis thaliana OX=3702 GN=LHCB4.3 PE=2 SV=1	-1.018
Cs9g06360	K00975|2.1e-69|cic:CICLE_v10004731mg|K00975 glucose-1-phosphate adenylyltransferase [EC:2.7.7.27] | (RefSeq) hypothetical protein	Glucose-1-phosphate adenylyltransferase large subunit 1, chloroplastic OS=Arabidopsis thaliana OX=3702 GN=ADG2 PE=1 SV=3	-1.206
orange1.1t03280	K00927|9.7e-74|cic:CICLE_v10004891mg|K00927 phosphoglycerate kinase [EC:2.7.2.3] | (RefSeq) hypothetical protein	Phosphoglycerate kinase, chloroplastic OS=Nicotiana tabacum OX=4097 PE=2 SV=1	-1.999
orange1.1t06026	K03881|4.1e-45|bvg:809503|K03881 NADH-ubiquinone oxidoreductase chain 4 [EC:7.1.1.2] | (RefSeq) nad4, BevupMp106; NADH dehydrogenase subunit 4	NADH-ubiquinone oxidoreductase chain 4 OS=Arabidopsis thaliana OX=3702 GN=ND4 PE=2 SV=2	2.075

Energy derivation by oxidation of organic compounds (GO:0015980; adjusted P = 0.3682)			
Cs2g15520	K01595|6.0e-206|cit:102607204|K01595 phosphoenolpyruvate carboxylase [EC:4.1.1.31] | (RefSeq) phosphoenolpyruvate carboxylase 2	Phosphoenolpyruvate carboxylase 1 OS=Arabidopsis thaliana OX=3702 GN=PPC1 PE=1 SV=1	-2.477
Cs5g10730	K00026|2.1e-223|cit:102628532|K00026 malate dehydrogenase [EC:1.1.1.37] | (RefSeq) malate dehydrogenase, chloroplastic	Malate dehydrogenase, chloroplastic OS=Arabidopsis thaliana OX=3702 GN=At3g47520 PE=1 SV=1	-1.256
Cs5g16400	K03934|1.5e-188|cit:102610171|K03934 NADH dehydrogenase (ubiquinone) Fe-S protein 1 [EC:7.1.1.2] | (RefSeq) NADH dehydrogenase [ubiquinone] iron-sulfur protein 1, mitochondrial	NADH dehydrogenase [ubiquinone] iron-sulfur protein 1, mitochondrial OS=Solanum tuberosum OX=4113 PE=2 SV=1	-1.047
Cs7g25390	K00026|1.5e-53|cic:CICLE_v10008562mg|K00026 malate dehydrogenase [EC:1.1.1.37] | (RefSeq) hypothetical protein	Malate dehydrogenase, mitochondrial OS=Citrullus lanatus OX=3654 GN=MMDH PE=1 SV=1	-1.555
Cs9g06360	K00975|2.1e-69|cic:CICLE_v10004731mg|K00975 glucose-1-phosphate adenylyltransferase [EC:2.7.7.27] | (RefSeq) hypothetical protein	Glucose-1-phosphate adenylyltransferase large subunit 1, chloroplastic OS=Arabidopsis thaliana OX=3702 GN=ADG2 PE=1 SV=3	-1.206
orange1.1t06026	K03881|4.1e-45|bvg:809503|K03881 NADH-ubiquinone oxidoreductase chain 4 [EC:7.1.1.2] | (RefSeq) nad4, BevupMp106; NADH dehydrogenase subunit 4	NADH-ubiquinone oxidoreductase chain 4 OS=Arabidopsis thaliana OX=3702 GN=ND4 PE=2 SV=2	2.075

Table S9. Differentially expressed genes (DEGs) related to nitrogen, protein and amino acid metabolisms in low pH-treated *Citrus sinensis* leaves

Accession No.	KEGG_annotation	Swissprot_annotation (NR_annotation)	Log_2(fold change)
Nitrogen metabolism (cit00910; corrected P = 0.0773)			
Cs3g19060	K10534|0.0e+00|cit:102610866|K10534 nitrate reductase (NAD(P)H) [EC:1.7.1.1 1.7.1.2 1.7.1.3] | (RefSeq) nitrate reductase [NAD(P)H]-like	Nitrate reductase [NADH] OS=Cucurbita maxima OX=3661 PE=2 SV=1	-1.351
Cs8g05970	K00366|1.2e-181|cit:102616714|K00366 ferredoxin-nitrite reductase [EC:1.7.7.1] | (RefSeq) ferredoxin--nitrite reductase, chloroplastic	Ferredoxin--nitrite reductase, chloroplastic OS=Betula pendula OX=3505 GN=NIR1 PE=2 SV=1	-1.226
Cs8g10280	K01673|8.3e-34|cic:CICLE_v10028905mg|K01673 carbonic anhydrase [EC:4.2.1.1] | (RefSeq) hypothetical protein	Beta carbonic anhydrase 5, chloroplastic OS=Arabidopsis thaliana OX=3702 GN=BCA5 PE=2 SV=1	1.014
Cs9g05680	K01915|7.0e-71|cic:CICLE_v10005007mg|K01915 glutamine synthetase [EC:6.3.1.2] | (RefSeq) hypothetical protein	Glutamine synthetase, chloroplastic OS=Daucus carota OX=4039 GN=GLN2 PE=2 SV=1	-1.445
Cellular nitrogen compound biosynthetic process (GO:0044271, adjusted P = 0.9389)			
Cs1g06850	K00218|5.4e-124|cit:102614959|K00218 protochlorophyllide reductase [EC:1.3.1.33] | (RefSeq) protochlorophyllide reductase, chloroplastic	Protochlorophyllide reductase, chloroplastic OS=Cucumis sativus OX=3659 GN=PORA PE=2 SV=1	-1.307
Cs1g14970	K06215|3.8e-168|cit:102610004|K06215 pyridoxal 5'-phosphate synthase pdxS subunit [EC:4.3.3.6] | (RefSeq) pyridoxal 5'-phosphate synthase-like subunit PDX1.2	Pyridoxal 5'-phosphate synthase-like subunit PDX1.2 OS=Arabidopsis thaliana OX=3702 GN=PDX12 PE=1 SV=1	-1.362
Cs1g21370		Basic leucine zipper 4 OS=Arabidopsis thaliana OX=3702 GN=BZIP4 PE=1 SV=1	1.240
Cs1g21640	K11338|2.5e-132|cit:102628941|K11338 RuvB-like protein 2 [EC:3.6.4.12] | (RefSeq) ruvB-like 2	RuvB-like protein 1 OS=Arabidopsis thaliana OX=3702 GN=RIN1 PE=1 SV=1	-1.199
Cs1g21890		Ribonuclease III domain-containing protein RNC1, chloroplastic OS=Arabidopsis thaliana OX=3702 GN=RNC1 PE=2 SV=1	-1.344
Cs2g03080	K02115|4.3e-195|cit:102629991|K02115 F-type H+-transporting ATPase subunit gamma | (RefSeq) ATP synthase gamma chain, chloroplastic	ATP synthase gamma chain, chloroplastic OS=Nicotiana tabacum OX=4097 GN=ATPC PE=1 SV=1	-1.309
Cs2g05100	K03405|6.0e-194|cit:102620862|K03405 magnesium chelatase subunit I [EC:6.6.1.1] | (RefSeq) magnesium-chelatase subunit ChlI, chloroplastic	Magnesium-chelatase subunit ChlI, chloroplastic OS=Glycine max OX=3847 GN=CHLI PE=2 SV=1	-1.158

Cs2g06020	K02888|2.2e-65|cic:CICLE_v10016378mg|K02888 large subunit ribosomal protein L21 | (RefSeq) hypothetical protein	50S ribosomal protein L21, mitochondrial OS=Arabidopsis thaliana OX=3702 GN=RPL21M PE=1 SV=1	-1.240
Cs2g06400	K20043|6.5e-23|aip:107640400|K20043 clathrin coat assembly protein AP180 | (RefSeq) putative clathrin assembly protein At5g35200	Dof zinc finger protein DOF3.4 OS=Arabidopsis thaliana OX=3702 GN=DOF3.4 PE=1 SV=2	-2.300
Cs2g07130			1.324
Cs2g13230	K12135|9.4e-06|eus:EUTSA_v10015486mg|K12135 zinc finger protein CONSTANS | (RefSeq) hypothetical protein	Putative zinc finger protein At1g68190 OS=Arabidopsis thaliana OX=3702 GN=At1g68190 PE=2 SV=1	1.773
Cs2g17040		HMG-Y-related protein A OS=Glycine max OX=3847 PE=2 SV=1	-1.194
Cs2g19240	K23330|6.7e-15|mis:MICPUN_100143|K23330 leucine-zipper-like transcriptional regulator 1 | (RefSeq) predicted protein	Nitrile-specifier protein 2 OS=Arabidopsis thaliana OX=3702 GN=NSP2 PE=2 SV=1	1.406
Cs2g19360	K08065|4.2e-74|cit:102616254|K08065 nuclear transcription Y subunit beta | (RefSeq) nuclear transcription factor Y subunit B-3	Nuclear transcription factor Y subunit B-2 OS=Arabidopsis thaliana OX=3702 GN=NFYB2 PE=1 SV=1	1.345
Cs3g01350	K00521|2.7e-68|cmos:111447866|K00521 ferric-chelate reductase [EC:1.16.1.7] | (RefSeq) ferric reduction oxidase 7, chloroplastic-like	NAC domain-containing protein 35 OS=Arabidopsis thaliana OX=3702 GN=NAC035 PE=1 SV=2	-1.715
Cs3g15140	K05953|7.2e-181|cit:102621359|K05953 nicotianamine synthase [EC:2.5.1.43] | (RefSeq) nicotianamine synthase	Nicotianamine synthase OS=Solanum lycopersicum OX=4081 GN=CHLN PE=2 SV=1	-2.242
Cs3g16730	K02492|4.2e-206|cit:102624392|K02492 glutamyl-tRNA reductase [EC:1.2.1.70] | (RefSeq) glutamyl-tRNA reductase 1, chloroplastic	Glutamyl-tRNA reductase 1, chloroplastic OS=Cucumis sativus OX=3659 GN=HEMA1 PE=2 SV=1	-1.277
Cs3g19060	K10534|0.0e+00|cit:102610866|K10534 nitrate reductase (NAD(P)H) [EC:1.7.1.1 1.7.1.2 1.7.1.3] | (RefSeq) nitrate reductase [NAD(P)H]-like	Nitrate reductase [NADH] OS=Cucurbita maxima OX=3661 PE=2 SV=1	-1.351
Cs4g02080	K10908|2.8e-170|cit:102620149|K10908 DNA-directed RNA polymerase, mitochondrial [EC:2.7.7.6] | (RefSeq) DNA-directed RNA polymerase 3, chloroplastic	DNA-directed RNA polymerase 3B, chloroplastic OS=Nicotiana tabacum OX=4097 GN=RPOT3-TOM PE=2 SV=2	-1.873
Cs4g07830	K02259|2.5e-61|cit:102630339|K02259 cytochrome c oxidase assembly protein subunit 15 | (RefSeq) cytochrome c oxidase assembly protein COX15	Cytochrome c oxidase assembly protein COX15 OS=Arabidopsis thaliana OX=3702 GN=COX15 PE=2 SV=1	-1.542
Cs4g11870		Transcription factor bHLH123 OS=Arabidopsis thaliana OX=3702 GN=BHLH123 PE=1 SV=1	-1.838
Cs4g15820	K09419|1.2e-94|cit:102615136|K09419 heat shock transcription factor, other eukaryote | (RefSeq) heat stress transcription factor C-1	Heat stress transcription factor C-1 OS=Arabidopsis thaliana OX=3702 GN=HSFC1 PE=1 SV=1	1.861

Cs4g16220	K14484 | 2.3e-69 | cit:102628728 | K14484 auxin-responsive protein IAA | (RefSeq) auxin-responsive protein IAA27 isoform X1	Auxin-responsive protein IAA8 OS=Arabidopsis thaliana OX=3702 GN=IAA8 PE=1 SV=1	3.330
Cs4g16260	K02357 | 2.0e-148 | cic:CICLE_v10007553mg | K02357 elongation factor Ts | (RefSeq) hypothetical protein	Polyprotein of EF-Ts, chloroplastic OS=Oryza sativa subsp. indica OX=39946 GN=PETs PE=3 SV=1	-1.927
Cs5g01380	K22382 | 4.2e-11 | oeu:111408549 | K22382 WD repeat-containing protein 26 | (RefSeq) WD repeat-containing protein 26 homolog	Growth-regulating factor 8 OS=Arabidopsis thaliana OX=3702 GN=GRF8 PE=2 SV=1	1.459
Cs5g03510	K01951 | 1.9e-120 | cic:CICLE_v10019676mg | K01951 GMP synthase (glutamine-hydrolysing) [EC:6.3.5.2] | (RefSeq) hypothetical protein	(Hypothetical protein CUMW_041950 [Citrus unshiu])	-1.355
Cs5g05515	K09680 | 2.6e-54 | cit:102629945 | K09680 type II pantothenate kinase [EC:2.7.1.33] | (RefSeq) pantothenate kinase 2	Pantothenate kinase 2 OS=Arabidopsis thaliana OX=3702 GN=PANK2 PE=1 SV=2	-1.247
Cs5g06190	K02358 | 1.6e-262 | cit:102625264 | K02358 elongation factor Tu | (RefSeq) elongation factor Tu, chloroplastic	Elongation factor Tu, chloroplastic OS=Glycine max OX=3847 GN=TUFA PE=3 SV=1	-1.437
Cs5g06590	K04498 | 2.2e-06 | mesc:110630983 | K04498 E1A/CREB-binding protein [EC:2.3.1.48] | (RefSeq) histone acetyltransferase HAC12-like isoform X1	BTB/POZ and TAZ domain-containing protein 1 OS=Arabidopsis thaliana OX=3702 GN=BT1 PE=1 SV=1	-1.874
Cs5g07120	K01940 | 1.1e-109 | cit:102630538 | K01940 argininosuccinate synthase [EC:6.3.4.5] | (RefSeq) argininosuccinate synthase, chloroplastic	Argininosuccinate synthase, chloroplastic OS=Arabidopsis thaliana OX=3702 GN=At4g24830 PE=1 SV=3	-1.032
Cs5g09330	K02355 | 0.0e+00 | cit:102624603 | K02355 elongation factor G | (RefSeq) elongation factor G-2, chloroplastic	Elongation factor G-2, chloroplastic OS=Glycine max OX=3847 GN=fusA2 PE=3 SV=1	-1.711
Cs5g10740	K10960 | 3.6e-141 | cic:CICLE_v10020061mg | K10960 geranylgeranyl diphosphate/geranylgeranyl-bacteriochlorophyllide a reductase [EC:1.3.1.83 1.3.1.111] | (RefSeq) hypothetical protein	Geranylgeranyl diphosphate reductase, chloroplastic OS=Arabidopsis thaliana OX=3702 GN=CHLP PE=1 SV=1	-1.716
Cs5g12780	K17413 | 1.3e-74 | cit:102631054 | K17413 small subunit ribosomal protein S35 | (RefSeq) uncharacterized protein LOC102631054	(Hypothetical protein CISIN_1g014729mg [Citrus sinensis])	-1.352
Cs5g15470	K00521 | 2.4e-19 | cmos:111447866 | K00521 ferric-chelate reductase [EC:1.16.1.7] | (RefSeq) ferric reduction oxidase 7, chloroplastic-like	NAC domain-containing protein 21/22 OS=Arabidopsis thaliana OX=3702 GN=NAC021 PE=1 SV=2	-1.170
Cs5g19690	K11808 | 1.6e-06 | qsu:111985940 | K11808 phosphoribosylaminoimidazole carboxylase [EC:4.1.1.21] | (RefSeq) phosphoribosylaminoimidazole carboxylase, chloroplastic-like isoform X1	(Hypothetical protein CUMW_218330 [Citrus unshiu])	-2.035
Cs5g25440		Protein ILITYHIA OS=Arabidopsis thaliana OX=3702 GN=ILA PE=1 SV=1	-1.084

Cs5g29460	K05941|7.2e-112|cic:CICLE_v10024202mg|K05941 glutathione gamma-glutamylcysteinyltransferase [EC:2.3.2.15] | (RefSeq) hypothetical protein	Glutathione gamma-glutamylcysteinyltransferase 2 OS=Lotus japonicus OX=34305 GN=PCS2 PE=2 SV=2	4.419
Cs5g30230	K11275|2.9e-48|cic:CICLE_v10021453mg|K11275 histone H1/5 | (RefSeq) hypothetical protein	Histone H1 OS=Euphorbia esula OX=3993 PE=2 SV=1	-2.447
Cs5g32250	K14549|6.2e-296|cic:CICLE_v10019671mg|K14549 U3 small nucleolar RNA-associated protein 15 | (RefSeq) hypothetical protein	Protein SLOW WALKER 1 OS=Arabidopsis thaliana OX=3702 GN=SWA1 PE=2 SV=1	-1.526
Cs6g04090	K02930|1.4e-202|cic:CICLE_v10011875mg|K02930 large subunit ribosomal protein L4e | (RefSeq) hypothetical protein	60S ribosomal protein L4 OS=Prunus armeniaca OX=36596 GN=RPL4 PE=2 SV=1	-1.093
Cs6g10620	K02879|1.5e-71|cic:CICLE_v10012716mg|K02879 large subunit ribosomal protein L17 | (RefSeq) hypothetical protein	50S ribosomal protein L17, chloroplastic OS=Nicotiana tabacum OX=4097 GN=RPL17 PE=2 SV=1	-1.210
Cs6g19900	K10908|6.3e-221|cit:102622473|K10908 DNA-directed RNA polymerase, mitochondrial [EC:2.7.7.6] | (RefSeq) DNA-directed RNA polymerase 2, chloroplastic/mitochondrial isoform X1	DNA-directed RNA polymerase 2, chloroplastic/mitochondrial OS=Nicotiana sylvestris OX=4096 GN=RPOT2 PE=2 SV=2	-1.441
Cs7g01720	K08064|1.1e-86|cic:CICLE_v10032207mg|K08064 nuclear transcription factor Y, alpha | (RefSeq) hypothetical protein	Nuclear transcription factor Y subunit A-9 OS=Arabidopsis thaliana OX=3702 GN=NFYA9 PE=1 SV=1	1.178
Cs7g03650	K15032|4.2e-150|cit:102611895|K15032 mTERF domain-containing protein, mitochondrial | (RefSeq) transcription termination factor MTEF1, chloroplastic	Transcription termination factor MTEF1, chloroplastic OS=Arabidopsis thaliana OX=3702 GN=MTERF1 PE=2 SV=2	-1.142
Cs7g03670	K16222|1.6e-61|csat:104740088|K16222 Dof zinc finger protein DOF5.5 | (RefSeq) cyclic dof factor 1	Cyclic dof factor 3 OS=Arabidopsis thaliana OX=3702 GN=CDF3 PE=1 SV=2	1.004
Cs7g03680		Myb-related protein 2 OS=Arabidopsis thaliana OX=3702 GN=MYR2 PE=1 SV=1	1.366
Cs7g08170		(Uncharacterized protein LOC18045706 [Citrus clementina])	-1.016
Cs7g10960	K09264|1.1e-09|cit:102618544|K09264 MADS-box transcription factor, plant | (RefSeq) MADS-box transcription factor 14-like isoform X1	(Hypothetical protein CISIN_1g035665mg, partial [Citrus sinensis])	1.524
Cs7g11680	K00847|1.1e-19|egr:104449325|K00847 fructokinase [EC:2.7.1.4] | (RefSeq) probable fructokinase-7	Fructokinase-like 2, chloroplastic OS=Arabidopsis thaliana OX=3702 GN=FLN2 PE=1 SV=2	-2.248
Cs7g11770		Squamosa promoter-binding-like protein 6 OS=Arabidopsis thaliana OX=3702 GN=SPL6 PE=1 SV=2	1.198

Cs7g12410	K01611|9.7e-208|cit:102578065|K01611 S-adenosylmethionine decarboxylase [EC:4.1.1.50] | (RefSeq) SAMDC; S-adenosylmethionine decarboxylase	S-adenosylmethionine decarboxylase proenzyme OS=Nicotiana tabacum OX=4097 GN=SAMDC PE=2 SV=1	-1.653
Cs7g13850	K01749|3.5e-93|cic:CICLE_v10031858mg|K01749 hydroxymethylbilane synthase [EC:2.5.1.61] | (RefSeq) hypothetical protein	Porphobilinogen deaminase, chloroplastic OS=Pisum sativum OX=3888 GN=HEMC PE=1 SV=1	-1.414
Cs7g15400	K02988|7.3e-169|cic:CICLE_v10031158mg|K02988 small subunit ribosomal protein S5 | (RefSeq) hypothetical protein	(Uncharacterized protein LOC18044585 [Citrus clementina])	-1.044
Cs7g16040	K02906|2.5e-99|cic:CICLE_v10032370mg|K02906 large subunit ribosomal protein L3 | (RefSeq) hypothetical protein	50S ribosomal protein L3-1, chloroplastic OS=Arabidopsis thaliana OX=3702 GN=RPL3A PE=2 SV=1	-1.133
Cs8g01080	K15032|7.4e-81|cit:102627586|K15032 mTERF domain-containing protein, mitochondrial | (RefSeq) transcription termination factor MTERF6, chloroplastic/mitochondrial	Transcription termination factor MTERF6, chloroplastic/mitochondrial OS=Arabidopsis thaliana OX=3702 GN=MTERF6 PE=2 SV=1	-1.220
Cs8g01360	K01885|8.0e-87|cit:102612397|K01885 glutamyl-tRNA synthetase [EC:6.1.1.17] | (RefSeq) glutamate--tRNA ligase, chloroplastic/mitochondrial	Glutamate--tRNA ligase, chloroplastic/mitochondrial OS=Arabidopsis thaliana OX=3702 GN=OVA3 PE=1 SV=1	-1.006
Cs8g06380	K15032|2.0e-81|cit:102629419|K15032 mTERF domain-containing protein, mitochondrial | (RefSeq) transcription termination factor MTERF2, chloroplastic	Transcription termination factor MTERF2, chloroplastic OS=Arabidopsis thaliana OX=3702 GN=MTERF2 PE=3 SV=1	-1.062
Cs8g07660	K00764|7.6e-168|cit:102622004|K00764 amidophosphoribosyltransferase [EC:2.4.2.14] | (RefSeq) LOW QUALITY PROTEIN: amidophosphoribosyltransferase, chloroplastic-like	Amidophosphoribosyltransferase 2, chloroplastic OS=Arabidopsis thaliana OX=3702 GN=ASE2 PE=1 SV=1	-1.418
Cs8g10730	K01880|1.3e-196|cit:102621048|K01880 glycyl-tRNA synthetase [EC:6.1.1.14] | (RefSeq) glycine--tRNA ligase, mitochondrial 1-like	Glycine--tRNA ligase, mitochondrial 1 OS=Arabidopsis thaliana OX=3702 GN=At1g29880 PE=1 SV=1	-1.088
Cs8g13200	K11808|5.1e-73|cit:102626933|K11808 phosphoribosylaminoimidazole carboxylase [EC:4.1.1.21] | (RefSeq) phosphoribosylaminoimidazole carboxylase, chloroplastic isoform X1	Phosphoribosylaminoimidazole carboxylase, chloroplastic (Fragment) OS=Vigna aconitifolia OX=3918 GN=PURKE PE=2 SV=1	-1.164
Cs8g20230	K06215|4.4e-175|cic:CICLE_v10028683mg|K06215 pyridoxal 5'-phosphate synthase pdxS subunit [EC:4.3.3.6] | (RefSeq) hypothetical protein	Probable pyridoxal 5'-phosphate synthase subunit PDX1 OS=Hevea brasiliensis OX=3981 GN=PDX1 PE=2 SV=1	-1.356
Cs9g03370			-1.201

Cs9g03750	K14844|4.6e-96|cit:102625301|K14844 pumilio homology domain family member 6 | (RefSeq) pumilio homolog 24 isoform X1	Pumilio homolog 24 OS=Arabidopsis thaliana OX=3702 GN=APUM24 PE=1 SV=1	-1.349
Cs9g04410	K01870|8.8e-102|cit:102618088|K01870 isoleucyl-tRNA synthetase [EC:6.1.1.5] | (RefSeq) isoleucine--tRNA ligase, chloroplastic/mitochondrial	Isoleucine--tRNA ligase, chloroplastic/mitochondrial OS=Arabidopsis thaliana OX=3702 GN=OVA2 PE=2 SV=1	-1.177
Cs9g04920	K19073|8.5e-235|cit:102611623|K19073 divinyl chlorophyllide a 8-vinyl-reductase [EC:1.3.1.75] | (RefSeq) divinyl chlorophyllide a 8-vinyl-reductase, chloroplastic	Divinyl chlorophyllide a 8-vinyl-reductase, chloroplastic OS=Cucumis sativus OX=3659 GN=DVR PE=1 SV=1	-1.624
Cs9g06680	K18787|6.3e-59|cic:CICLE_v10005374mg|K18787 thermospermine synthase [EC:2.5.1.79] | (RefSeq) hypothetical protein	Thermospermine synthase ACAULIS5 OS=Arabidopsis thaliana OX=3702 GN=ACL5 PE=1 SV=1	1.929
Cs9g06870	K02974|2.3e-25|cic:CICLE_v10006194mg|K02974 small subunit ribosomal protein S24e | (RefSeq) hypothetical protein	40S ribosomal protein S24-1 OS=Arabidopsis thaliana OX=3702 GN=RPS24A PE=2 SV=1	1.242
Cs9g07680	K17290|1.9e-09|apro:F751_3177|K17290 oxidoreductase [EC:1.1.1.-] | (RefSeq) Oxidoreductase HTATIP2	Early light-induced protein 1, chloroplastic OS=Arabidopsis thaliana OX=3702 GN=ELIP1 PE=1 SV=1	-1.298
Cs9g08110	K14484|4.1e-47|cic:CICLE_v10005789mg|K14484 auxin-responsive protein IAA | (RefSeq) hypothetical protein	Auxin-responsive protein IAA17 OS=Arabidopsis thaliana OX=3702 GN=IAA17 PE=1 SV=2	1.138
orange1.1t00409	K02931|6.5e-73|cic:CICLE_v10021696mg|K02931 large subunit ribosomal protein L5 | (RefSeq) hypothetical protein	50S ribosomal protein L5, chloroplastic OS=Arabidopsis thaliana OX=3702 GN=RPL5 PE=2 SV=1	-1.326
orange1.1t01583		B-box zinc finger protein 22 OS=Arabidopsis thaliana OX=3702 GN=BBX22 PE=1 SV=2	-2.224
orange1.1t01850	K14492|5.7e-65|cit:102607514|K14492 two-component response regulator ARR-A family | (RefSeq) two-component response regulator ORR10	Two-component response regulator ARR9 OS=Arabidopsis thaliana OX=3702 GN=ARR9 PE=1 SV=1	-1.171
orange1.1t01948	K02925|4.7e-182|cit:102611748|K02925 large subunit ribosomal protein L3e | (RefSeq) 60S ribosomal protein L3-2	60S ribosomal protein L3-2 OS=Arabidopsis thaliana OX=3702 GN=ARP2 PE=2 SV=4	-1.227
orange1.1t02555	K22382|1.8e-13|oeu:111408549|K22382 WD repeat-containing protein 26 | (RefSeq) WD repeat-containing protein 26 homolog	Growth-regulating factor 1 OS=Zea mays OX=4577 GN=GRF1 PE=1 SV=1	3.369
orange1.1t04239	K03125|4.0e-74|cic:CICLE_v10004140mg|K03125 transcription initiation factor TFIID subunit 1 [EC:2.3.1.48 2.7.11.1] | (RefSeq) hypothetical protein	Transcription initiation factor TFIID subunit 1 OS=Arabidopsis thaliana OX=3702 GN=TAF1 PE=1 SV=1	-1.305
orange1.1t04338	K14760|8.7e-55|mcha:111015751|K14760 acyl-activating enzyme 14 [EC:6.2.1.26] | (RefSeq) 2-succinylbenzoate--CoA ligase, chloroplastic/peroxisomal isoform X1	(Hypothetical protein CISIN_1g002828mg [Citrus sinensis])	-1.144

orange1.1t04599		BEL1-like homeodomain protein 1 OS=Arabidopsis thaliana OX=3702 GN=BLH1 PE=1 SV=1	-1.131
orange1.1t04817	K02874|1.3e-29|cit:4271188|K02874 large subunit ribosomal protein L14 | (RefSeq) rpl14, CisiCp058; ribosomal protein L14	50S ribosomal protein L14, chloroplastic OS=Carica papaya OX=3649 GN=rpl14 PE=3 SV=1	-1.143
Cellular nitrogen compound catabolic process (GO:0044270; adjusted P = 1.0000)			
Cs2g18380	K01166|7.9e-45|cit:102616155|K01166 ribonuclease T2 [EC:4.6.1.19] | (RefSeq) ribonuclease 2	Ribonuclease 2 OS=Arabidopsis thaliana OX=3702 GN=RNS2 PE=2 SV=1	1.289
Cs7g25620	K12613|1.6e-58|cic:CICLE_v10032124mg|K12613 mRNA-decapping enzyme subunit 2 [EC:3.6.1.62] | (RefSeq) hypothetical protein	mRNA-decapping enzyme subunit 2 OS=Arabidopsis thaliana OX=3702 GN=DCP2 PE=1 SV=1	1.042
Protein metabolic process (GO:0019538; adjusted P = 1.0000)			
Cs1g09310	K02689|1.7e-31|aip:110272002|K02689 photosystem I P700 chlorophyll a apoprotein A1 | (RefSeq) uncharacterized protein LOC110272002	Photosystem I P700 chlorophyll a apoprotein A1 OS=Agrostis stolonifera OX=63632 GN=psaA PE=3 SV=1	-1.422
Cs1g14893	K24104|1.1e-35|thj:104799061|K24104 GPN-loop GTPase | (RefSeq) LOW QUALITY PROTEIN: uncharacterized protein LOC104799061	Receptor-like serine/threonine-protein kinase SD1-6 OS=Arabidopsis thaliana OX=3702 GN=SD16 PE=1 SV=2	1.262
Cs1g19910	K14498|2.3e-88|cit:102623202|K14498 serine/threonine-protein kinase SRK2 [EC:2.7.11.1] | (RefSeq) serine/threonine-protein kinase SAPK2 isoform X1	Serine/threonine-protein kinase SAPK2 OS=Oryza sativa subsp. indica OX=39946 GN=SAPK2 PE=2 SV=2	1.270
Cs1g21640	K11338|2.5e-132|cit:102628941|K11338 RuvB-like protein 2 [EC:3.6.4.12] | (RefSeq) ruvB-like 2	RuvB-like protein 1 OS=Arabidopsis thaliana OX=3702 GN=RIN1 PE=1 SV=1	-1.199
Cs1g21890		Ribonuclease III domain-containing protein RNC1, chloroplastic OS=Arabidopsis thaliana OX=3702 GN=RNC1 PE=2 SV=1	-1.344
Cs2g06020	K02888|2.2e-65|cic:CICLE_v10016378mg|K02888 large subunit ribosomal protein L21 | (RefSeq) hypothetical protein	50S ribosomal protein L21, mitochondrial OS=Arabidopsis thaliana OX=3702 GN=RPL21M PE=1 SV=1	-1.240
Cs2g09030	K10268|3.2e-99|cit:102614204|K10268 F-box and leucine-rich repeat protein 2/20 | (RefSeq) F-box/LRR-repeat protein 3-like	F-box/LRR-repeat protein 3 OS=Arabidopsis thaliana OX=3702 GN=FBL3 PE=2 SV=1	1.752
Cs2g09070	K02703|1.7e-97|aly:32283147|K02703 photosystem II P680 reaction center D1 protein [EC:1.10.3.9] | (RefSeq) psbA, B9N47_pgp099; photosystem II protein D1	Photosystem II protein D1 OS=Anthoceros angustus OX=48387 GN=psbA PE=2 SV=1	-2.156

Cs2g10550	K01180|2.6e-27|cmax:111487562|K01180 endo-1,3(4)-beta-glucanase [EC:3.2.1.6] | (RefSeq) uncharacterized protein LOC111487562	Probable LRR receptor-like serine/threonine-protein kinase At1g56130 OS=Arabidopsis thaliana OX=3702 GN=At1g56130 PE=2 SV=2	1.345
Cs2g10710	K19042|7.1e-93|cit:102618190|K19042 E3 ubiquitin-protein ligase BOI and related proteins [EC:2.3.2.27] | (RefSeq) BOI-related E3 ubiquitin-protein ligase 1	E3 ubiquitin-protein ligase BOI OS=Arabidopsis thaliana OX=3702 GN=BOI PE=1 SV=1	1.097
Cs2g15180	K03798|3.4e-271|cit:102623114|K03798 cell division protease FtsH [EC:3.4.24.-] | (RefSeq) ATP-dependent zinc metalloprotease FTSH 2, chloroplastic isoform X1	ATP-dependent zinc metalloprotease FTSH 2, chloroplastic OS=Arabidopsis thaliana OX=3702 GN=FTSH2 PE=1 SV=1	-1.619
Cs2g19040	K16285|5.4e-88|cic:CICLE_v10017032mg|K16285 RING/U-box domain-containing protein [EC:2.3.2.27] | (RefSeq) hypothetical protein	Probable E3 ubiquitin-protein ligase XERICO OS=Arabidopsis thaliana OX=3702 GN=XERICO PE=1 SV=1	1.329
Cs3g02720		(Uncharacterized protein LOC102623398 isoform X1 [Citrus sinensis])	1.063
Cs3g06200	K02706|1.3e-87|aly:32283168|K02706 photosystem II P680 reaction center D2 protein [EC:1.10.3.9] | (RefSeq) psbD, B9N47_pgp079; photosystem II protein D2	Photosystem II D2 protein OS=Acorus americanus OX=263995 GN=psbD PE=3 SV=1	-1.894
Cs3g15120	K08675|7.4e-155|cit:102620332|K08675 ATP-dependent lon protease [EC:3.4.21.53] | (RefSeq) lon protease homolog 1, mitochondrial	Lon protease homolog, mitochondrial OS=Oryza sativa subsp. indica OX=39946 GN=OsI_27415 PE=2 SV=1	-1.117
Cs3g27030	K03798|3.5e-297|cit:102607431|K03798 cell division protease FtsH [EC:3.4.24.-] | (RefSeq) ATP-dependent zinc metalloprotease FTSH, chloroplastic	ATP-dependent zinc metalloprotease FTSH 5, chloroplastic OS=Arabidopsis thaliana OX=3702 GN=FTSH5 PE=1 SV=1	-1.218
Cs3g27250	K21777|7.0e-46|cit:102615702|K21777 G2/mitotic-specific cyclin-B, other | (RefSeq) cyclin-B2-4	G2/mitotic-specific cyclin-2 (Fragment) OS=Medicago sativa OX=3879 PE=2 SV=1	1.122
Cs4g06500		F-box only protein 13 OS=Arabidopsis thaliana OX=3702 GN=FBX13 PE=2 SV=2	-2.642
Cs4g06540	K16281|3.8e-44|jcu:105638045|K16281 RING-H2 zinc finger protein RHA1 | (RefSeq) E3 ubiquitin-protein ligase ATL4	Brassinosteroid-responsive RING protein 1 OS=Arabidopsis thaliana OX=3702 GN=BRH1 PE=2 SV=1	1.032
Cs4g07150	K08908|9.5e-112|cit:102607051|K08908 light-harvesting complex I chlorophyll a/b binding protein 2 | (RefSeq) photosystem I chlorophyll a/b-binding protein 6, chloroplastic isoform X1	Photosystem I chlorophyll a/b-binding protein 6, chloroplastic OS=Arabidopsis thaliana OX=3702 GN=LHCA6 PE=1 SV=1	-1.058
Cs4g16260	K02357|2.0e-148|cic:CICLE_v10007553mg|K02357 elongation factor Ts | (RefSeq) hypothetical protein	Polyprotein of EF-Ts, chloroplastic OS=Oryza sativa subsp. indica OX=39946 GN=PETs PE=3 SV=1	-1.927

Cs5g06190	K02358|1.6e-262|cit:102625264|K02358 elongation factor Tu | (RefSeq) elongation factor Tu, chloroplastic	Elongation factor Tu, chloroplastic OS=Glycine max OX=3847 GN=TUFA PE=3 SV=1	-1.437
Cs5g06200	K08867|2.2e-168|cit:102625534|K08867 WNK lysine deficient protein kinase [EC:2.7.11.1] | (RefSeq) probable serine/threonine-protein kinase WNK3 isoform X1	Probable serine/threonine-protein kinase WNK3 OS=Arabidopsis thaliana OX=3702 GN=WNK3 PE=2 SV=1	1.028
Cs5g06590	K04498|2.2e-06|mesc:110630983|K04498 E1A/CREB-binding protein [EC:2.3.1.48] | (RefSeq) histone acetyltransferase HAC12-like isoform X1	BTB/POZ and TAZ domain-containing protein 1 OS=Arabidopsis thaliana OX=3702 GN=BT1 PE=1 SV=1	-1.874
Cs5g09330	K02355|0.0e+00|cit:102624603|K02355 elongation factor G | (RefSeq) elongation factor G-2, chloroplastic	Elongation factor G-2, chloroplastic OS=Glycine max OX=3847 GN=fusA2 PE=3 SV=1	-1.711
Cs5g12780	K17413|1.3e-74|cit:102631054|K17413 small subunit ribosomal protein S35 | (RefSeq) uncharacterized protein LOC102631054	(Hypothetical protein CISIN_1g014729mg [Citrus sinensis])	-1.352
Cs5g22690	K13430|1.6e-63|dzi:111291965|K13430 serine/threonine-protein kinase PBS1 [EC:2.7.11.1] | (RefSeq) serine/threonine-protein kinase PBL27-like isoform X1	Serine/threonine-protein kinase-like protein ACR4 OS=Arabidopsis thaliana OX=3702 GN=ACR4 PE=1 SV=1	1.450
Cs5g25440		Protein ILITYHIA OS=Arabidopsis thaliana OX=3702 GN=ILA PE=1 SV=1	-1.084
Cs6g04090	K02930|1.4e-202|cic:CICLE_v10011875mg|K02930 large subunit ribosomal protein L4e | (RefSeq) hypothetical protein	60S ribosomal protein L4 OS=Prunus armeniaca OX=36596 GN=RPL4 PE=2 SV=1	-1.093
Cs6g04920	K14015|1.3e-184|cic:CICLE_v10011862mg|K14015 nuclear protein localization protein 4 homolog | (RefSeq) hypothetical protein	NPL4-like protein 1 OS=Arabidopsis thaliana OX=3702 GN=At3g63000 PE=1 SV=1	-1.010
Cs6g10620	K02879|1.5e-71|cic:CICLE_v10012716mg|K02879 large subunit ribosomal protein L17 | (RefSeq) hypothetical protein	50S ribosomal protein L17, chloroplastic OS=Nicotiana tabacum OX=4097 GN=RPL17 PE=2 SV=1	-1.210
Cs7g08170		(Uncharacterized protein LOC18045706 [Citrus clementina])	-1.016
Cs7g08350	K10663|1.9e-25|sind:105167419|K10663 E3 ubiquitin-protein ligase ATL4 [EC:2.3.2.27] | (RefSeq) E3 ubiquitin-protein ligase ATL4-like	RING-H2 finger protein ATL47 OS=Arabidopsis thaliana OX=3702 GN=ATL47 PE=2 SV=1	1.105
Cs7g08960		F-box/kelch-repeat protein At1g23390 OS=Arabidopsis thaliana OX=3702 GN=At1g23390 PE=2 SV=1	-1.265
Cs7g11140	K03768|9.0e-42|cit:102625552|K03768 peptidyl-prolyl cis-trans isomerase B (cyclophilin B) [EC:5.2.1.8] | (RefSeq) photosynthetic NDH subunit of lumenal location 5, chloroplastic	Photosynthetic NDH subunit of lumenal location 5, chloroplastic OS=Arabidopsis thaliana OX=3702 GN=PNSL5 PE=1 SV=1	-1.910
Cs7g13800	K14563|1.2e-81|cic:CICLE_v10032221mg|K14563 rRNA 2'-O-methyltransferase fibrillarin [EC:2.1.1.-] | (RefSeq) hypothetical protein	rRNA 2'-O-methyltransferase fibrillarin 1 OS=Arabidopsis thaliana OX=3702 GN=FIB1 PE=1 SV=1	-1.182

Cs7g13850	K01749|3.5e-93|cic:CICLE_v10031858mg|K01749 hydroxymethylbilane synthase [EC:2.5.1.61] | (RefSeq) hypothetical protein	Porphobilinogen deaminase, chloroplastic OS=Pisum sativum OX=3888 GN=HEMC PE=1 SV=1	-1.414
Cs7g13940	K16297|1.8e-115|cit:102608354|K16297 serine carboxypeptidase-like clade II [EC:3.4.16.-] | (RefSeq) serine carboxypeptidase-like 35	Serine carboxypeptidase-like 35 OS=Arabidopsis thaliana OX=3702 GN=SCPL35 PE=2 SV=1	1.140
Cs7g15400	K02988|7.3e-169|cic:CICLE_v10031158mg|K02988 small subunit ribosomal protein S5 | (RefSeq) hypothetical protein	(Uncharacterized protein LOC18044585 [Citrus clementina])	-1.044
Cs7g16040	K02906|2.5e-99|cic:CICLE_v10032370mg|K02906 large subunit ribosomal protein L3 | (RefSeq) hypothetical protein	50S ribosomal protein L3-1, chloroplastic OS=Arabidopsis thaliana OX=3702 GN=RPL3A PE=2 SV=1	-1.133
Cs7g16620	K09571|2.4e-84|cic:CICLE_v10031088mg|K09571 FK506-binding protein 4/5 [EC:5.2.1.8] | (RefSeq) hypothetical protein	70 kDa peptidyl-prolyl isomerase OS=Triticum aestivum OX=4565 GN=FKBP70 PE=1 SV=1	-1.225
Cs7g30500	K14496|1.2e-114|cic:CICLE_v10032781mg|K14496 abscisic acid receptor PYR/PYL family | (RefSeq) hypothetical protein	Abscisic acid receptor PYL4 OS=Arabidopsis thaliana OX=3702 GN=PYL4 PE=1 SV=1	-1.525
Cs7g31190	K08915|5.5e-137|cic:CICLE_v10032377mg|K08915 light-harvesting complex II chlorophyll a/b binding protein 4 | (RefSeq) hypothetical protein	Chlorophyll a-b binding protein CP29.3, chloroplastic OS=Arabidopsis thaliana OX=3702 GN=LHCB4.3 PE=2 SV=1	-1.018
Cs8g01360	K01885|8.0e-87|cit:102612397|K01885 glutamyl-tRNA synthetase [EC:6.1.1.17] | (RefSeq) glutamate--tRNA ligase, chloroplastic/mitochondrial	Glutamate--tRNA ligase, chloroplastic/mitochondrial OS=Arabidopsis thaliana OX=3702 GN=OVA3 PE=1 SV=1	-1.006
Cs8g08320	K01373|2.0e-115|cit:102620490|K01373 cathepsin F [EC:3.4.22.41] | (RefSeq) probable cysteine protease RD19B	Cysteine protease RD19A OS=Arabidopsis thaliana OX=3702 GN=RD19A PE=1 SV=1	-1.117
Cs8g10730	K01880|1.3e-196|cit:102621048|K01880 glycyl-tRNA synthetase [EC:6.1.1.14] | (RefSeq) glycine--tRNA ligase, mitochondrial 1-like	Glycine--tRNA ligase, mitochondrial 1 OS=Arabidopsis thaliana OX=3702 GN=At1g29880 PE=1 SV=1	-1.088
Cs8g15970	K13525|3.2e-159|cit:102612408|K13525 transitional endoplasmic reticulum ATPase | (RefSeq) cell division cycle protein 48 homolog	Cell division cycle protein 48 homolog OS=Glycine max OX=3847 GN=CDC48 PE=2 SV=1	-1.028
Cs9g03750	K14844|4.6e-96|cit:102625301|K14844 pumilio homology domain family member 6 | (RefSeq) pumilio homolog 24 isoform X1	Pumilio homolog 24 OS=Arabidopsis thaliana OX=3702 GN=APUM24 PE=1 SV=1	-1.349
Cs9g04410	K01870|8.8e-102|cit:102618088|K01870 isoleucyl-tRNA synthetase [EC:6.1.1.5] | (RefSeq) isoleucine--tRNA ligase, chloroplastic/mitochondrial	Isoleucine--tRNA ligase, chloroplastic/mitochondrial OS=Arabidopsis thaliana OX=3702 GN=OVA2 PE=2 SV=1	-1.177
Cs9g06720	K00276|1.0e-185|cic:CICLE_v10004484mg|K00276 primary-amine oxidase [EC:1.4.3.21] | (RefSeq) hypothetical protein	Primary amine oxidase (Fragment) OS=Lens culinaris OX=3864 PE=1 SV=3	-3.393
Cs9g06870	K02974|2.3e-25|cic:CICLE_v10006194mg|K02974 small subunit ribosomal protein S24e | (RefSeq) hypothetical protein	40S ribosomal protein S24-1 OS=Arabidopsis thaliana OX=3702 GN=RPS24A PE=2 SV=1	1.242

orange1.1t00405	K13430|2.7e-29|ccaj:109802387|K13430 serine/threonine-protein kinase PBS1 [EC:2.7.11.1] | (RefSeq) serine/threonine-protein kinase PBS1-like isoform X1	Probable serine/threonine-protein kinase At1g01540 OS=Arabidopsis thaliana OX=3702 GN=At1g01540 PE=1 SV=2	1.099
orange1.1t00409	K02931|6.5e-73|cic:CICLE_v10021696mg|K02931 large subunit ribosomal protein L5 | (RefSeq) hypothetical protein	50S ribosomal protein L5, chloroplastic OS=Arabidopsis thaliana OX=3702 GN=RPL5 PE=2 SV=1	-1.326
orange1.1t00499		(Hypothetical protein CUMW_091500 [Citrus unshiu])	-1.481
orange1.1t01687	K01358|1.5e-59|cic:CICLE_v10021318mg|K01358 ATP-dependent Clp protease, protease subunit [EC:3.4.21.92] | (RefSeq) hypothetical protein	ATP-dependent Clp protease proteolytic subunit 5, chloroplastic OS=Arabidopsis thaliana OX=3702 GN=CLPP5 PE=1 SV=1	-1.105
orange1.1t01948	K02925|4.7e-182|cit:102611748|K02925 large subunit ribosomal protein L3e | (RefSeq) 60S ribosomal protein L3-2	60S ribosomal protein L3-2 OS=Arabidopsis thaliana OX=3702 GN=ARP2 PE=2 SV=4	-1.227
orange1.1t03252	K03798|1.3e-100|cic:CICLE_v10004242mg|K03798 cell division protease FtsH [EC:3.4.24.-] | (RefSeq) hypothetical protein	ATP-dependent zinc metalloprotease FTSH 12, chloroplastic OS=Arabidopsis thaliana OX=3702 GN=FTSH12 PE=2 SV=2	-1.563
orange1.1t04239	K03125|4.0e-74|cic:CICLE_v10004140mg|K03125 transcription initiation factor TFIID subunit 1 [EC:2.3.1.48 2.7.11.1] | (RefSeq) hypothetical protein	Transcription initiation factor TFIID subunit 1 OS=Arabidopsis thaliana OX=3702 GN=TAF1 PE=1 SV=1	-1.305
orange1.1t04760	K13420|7.4e-110|cit:102626235|K13420 LRR receptor-like serine/threonine-protein kinase FLS2 [EC:2.7.11.1] | (RefSeq) probable LRR receptor-like serine/threonine-protein kinase At3g47570	Probable LRR receptor-like serine/threonine-protein kinase At3g47570 OS=Arabidopsis thaliana OX=3702 GN=At3g47570 PE=2 SV=1	1.925
orange1.1t04817	K02874|1.3e-29|cit:4271188|K02874 large subunit ribosomal protein L14 | (RefSeq) rpl14, CisiCp058; ribosomal protein L14	50S ribosomal protein L14, chloroplastic OS=Carica papaya OX=3649 GN=rpl14 PE=3 SV=1	-1.143
Protein catabolic process (GO:0030163; adjusted P = 0.9342)			
Cs2g09030	K10268|3.2e-99|cit:102614204|K10268 F-box and leucine-rich repeat protein 2/20 | (RefSeq) F-box/LRR-repeat protein 3-like	F-box/LRR-repeat protein 3 OS=Arabidopsis thaliana OX=3702 GN=FBL3 PE=2 SV=1	1.752
Cs2g15180	K03798|3.4e-271|cit:102623114|K03798 cell division protease FtsH [EC:3.4.24.-] | (RefSeq) ATP-dependent zinc metalloprotease FTSH 2, chloroplastic isoform X1	ATP-dependent zinc metalloprotease FTSH 2, chloroplastic OS=Arabidopsis thaliana OX=3702 GN=FTSH2 PE=1 SV=1	-1.619
Cs2g19040	K16285|5.4e-88|cic:CICLE_v10017032mg|K16285 RING/U-box domain-containing protein [EC:2.3.2.27] | (RefSeq) hypothetical protein	Probable E3 ubiquitin-protein ligase XERICO OS=Arabidopsis thaliana OX=3702 GN=XERICO PE=1 SV=1	1.329

Cs3g15120	K08675|7.4e-155|cit:102620332|K08675 ATP-dependent lon protease [EC:3.4.21.53] | (RefSeq) lon protease homolog 1, mitochondrial	Lon protease homolog, mitochondrial OS=Oryza sativa subsp. indica OX=39946 GN=OsI_27415 PE=2 SV=1	-1.117
Cs3g27030	K03798|3.5e-297|cit:102607431|K03798 cell division protease FtsH [EC:3.4.24.-] | (RefSeq) ATP-dependent zinc metalloprotease FTSH, chloroplastic	ATP-dependent zinc metalloprotease FTSH 5, chloroplastic OS=Arabidopsis thaliana OX=3702 GN=FTSH5 PE=1 SV=1	-1.218
Cs4g06500		F-box only protein 13 OS=Arabidopsis thaliana OX=3702 GN=FBX13 PE=2 SV=2	-2.642
Cs5g06590	K04498|2.2e-06|mesc:110630983|K04498 E1A/CREB-binding protein [EC:2.3.1.48] | (RefSeq) histone acetyltransferase HAC12-like isoform X1	BTB/POZ and TAZ domain-containing protein 1 OS=Arabidopsis thaliana OX=3702 GN=BT1 PE=1 SV=1	-1.874
Cs6g04920	K14015|1.3e-184|cic:CICLE_v10011862mg|K14015 nuclear protein localization protein 4 homolog | (RefSeq) hypothetical protein	NPL4-like protein 1 OS=Arabidopsis thaliana OX=3702 GN=At3g63000 PE=1 SV=1	-1.010
Cs7g08960		F-box/kelch-repeat protein At1g23390 OS=Arabidopsis thaliana OX=3702 GN=At1g23390 PE=2 SV=1	-1.265
Cs7g13940	K16297|1.8e-115|cit:102608354|K16297 serine carboxypeptidase-like clade II [EC:3.4.16.-] | (RefSeq) serine carboxypeptidase-like 35	Serine carboxypeptidase-like 35 OS=Arabidopsis thaliana OX=3702 GN=SCPL35 PE=2 SV=1	1.140
Cs8g08320	K01373|2.0e-115|cit:102620490|K01373 cathepsin F [EC:3.4.22.41] | (RefSeq) probable cysteine protease RD19B	Cysteine protease RD19A OS=Arabidopsis thaliana OX=3702 GN=RD19A PE=1 SV=1	-1.117
Cs8g15970	K13525|3.2e-159|cit:102612408|K13525 transitional endoplasmic reticulum ATPase | (RefSeq) cell division cycle protein 48 homolog	Cell division cycle protein 48 homolog OS=Glycine max OX=3847 GN=CDC48 PE=2 SV=1	-1.028
orange1.1t00499		(Hypothetical protein CUMW_091500 [Citrus unshiu])	-1.481
orange1.1t01687	K01358|1.5e-59|cic:CICLE_v10021318mg|K01358 ATP-dependent Clp protease, protease subunit [EC:3.4.21.92] | (RefSeq) hypothetical protein	ATP-dependent Clp protease proteolytic subunit 5, chloroplastic OS=Arabidopsis thaliana OX=3702 GN=CLPP5 PE=1 SV=1	-1.105
Protein ubiquitination (GO:0016567; adjusted P = 1.0000)			
Cs2g10710	K19042|7.1e-93|cit:102618190|K19042 E3 ubiquitin-protein ligase BOI and related proteins [EC:2.3.2.27] | (RefSeq) BOI-related E3 ubiquitin-protein ligase 1	E3 ubiquitin-protein ligase BOI OS=Arabidopsis thaliana OX=3702 GN=BOI PE=1 SV=1	1.097
Cs2g19040	K16285|5.4e-88|cic:CICLE_v10017032mg|K16285 RING/U-box domain-containing protein [EC:2.3.2.27] | (RefSeq) hypothetical protein	Probable E3 ubiquitin-protein ligase XERICO OS=Arabidopsis thaliana OX=3702 GN=XERICO PE=1 SV=1	1.329

Cs4g06500		F-box only protein 13 OS=Arabidopsis thaliana OX=3702 GN=FBX13 PE=2 SV=2	-2.642
Cs4g06540	K16281|3.8e-44|jcu:105638045|K16281 RING-H2 zinc finger protein RHA1 | (RefSeq) E3 ubiquitin-protein ligase ATL4	Brassinosteroid-responsive RING protein 1 OS=Arabidopsis thaliana OX=3702 GN=BRH1 PE=2 SV=1	1.032
Cs7g08350	K10663|1.9e-25|sind:105167419|K10663 E3 ubiquitin-protein ligase ATL4 [EC:2.3.2.27] | (RefSeq) E3 ubiquitin-protein ligase ATL4-like	RING-H2 finger protein ATL47 OS=Arabidopsis thaliana OX=3702 GN=ATL47 PE=2 SV=1	1.105
Proteasome-mediated ubiquitin-dependent protein catabolic process (GO:0043161; adjusted P = 0.9970)			
Cs2g09030	K10268|3.2e-99|cit:102614204|K10268 F-box and leucine-rich repeat protein 2/20 | (RefSeq) F-box/LRR-repeat protein 3-like	F-box/LRR-repeat protein 3 OS=Arabidopsis thaliana OX=3702 GN=FBL3 PE=2 SV=1	1.752
Cs2g19040	K16285|5.4e-88|cic:CICLE_v10017032mg|K16285 RING/U-box domain-containing protein [EC:2.3.2.27] | (RefSeq) hypothetical protein	Probable E3 ubiquitin-protein ligase XERICO OS=Arabidopsis thaliana OX=3702 GN=XERICO PE=1 SV=1	1.329
Cs4g06500		F-box only protein 13 OS=Arabidopsis thaliana OX=3702 GN=FBX13 PE=2 SV=2	-2.642
Cs5g06590	K04498|2.2e-06|mesc:110630983|K04498 E1A/CREB-binding protein [EC:2.3.1.48] | (RefSeq) histone acetyltransferase HAC12-like isoform X1	BTB/POZ and TAZ domain-containing protein 1 OS=Arabidopsis thaliana OX=3702 GN=BT1 PE=1 SV=1	-1.874
Cs7g08960		F-box/kelch-repeat protein At1g23390 OS=Arabidopsis thaliana OX=3702 GN=At1g23390 PE=2 SV=1	-1.265
Ubiquitin-dependent protein catabolic process (GO:0006511; adjusted P = 1.0000)			
Cs2g09030	K10268|3.2e-99|cit:102614204|K10268 F-box and leucine-rich repeat protein 2/20 | (RefSeq) F-box/LRR-repeat protein 3-like	F-box/LRR-repeat protein 3 OS=Arabidopsis thaliana OX=3702 GN=FBL3 PE=2 SV=1	1.752
Cs2g19040	K16285|5.4e-88|cic:CICLE_v10017032mg|K16285 RING/U-box domain-containing protein [EC:2.3.2.27] | (RefSeq) hypothetical protein	Probable E3 ubiquitin-protein ligase XERICO OS=Arabidopsis thaliana OX=3702 GN=XERICO PE=1 SV=1	1.329
Cs4g06500		F-box only protein 13 OS=Arabidopsis thaliana OX=3702 GN=FBX13 PE=2 SV=2	-2.642
Cs5g06590	K04498|2.2e-06|mesc:110630983|K04498 E1A/CREB-binding protein [EC:2.3.1.48] | (RefSeq) histone acetyltransferase HAC12-like isoform X1	BTB/POZ and TAZ domain-containing protein 1 OS=Arabidopsis thaliana OX=3702 GN=BT1 PE=1 SV=1	-1.874

Cs6g04920	K14015|1.3e-184|cic:CICLE_v10011862mg|K14015 nuclear protein localization protein 4 homolog | (RefSeq) hypothetical protein	NPL4-like protein 1 OS=Arabidopsis thaliana OX=3702 GN=At3g63000 PE=1 SV=1	-1.010
Cs7g08960		F-box/kelch-repeat protein At1g23390 OS=Arabidopsis thaliana OX=3702 GN=At1g23390 PE=2 SV=1	-1.265
orange1.1t00499		(Hypothetical protein CUMW_091500 [Citrus unshiu])	-1.481
Protein phosphorylation (GO:0006468, adjusted P = 1.0000)			
Cs1g14893	K24104|1.1e-35|thj:104799061|K24104 GPN-loop GTPase | (RefSeq) LOW QUALITY PROTEIN: uncharacterized protein LOC104799061	Receptor-like serine/threonine-protein kinase SD1-6 OS=Arabidopsis thaliana OX=3702 GN=SD16 PE=1 SV=2	1.262
Cs1g19910	K14498|2.3e-88|cit:102623202|K14498 serine/threonine-protein kinase SRK2 [EC:2.7.11.1] | (RefSeq) serine/threonine-protein kinase SAPK2 isoform X1	Serine/threonine-protein kinase SAPK2 OS=Oryza sativa subsp. indica OX=39946 GN=SAPK2 PE=2 SV=2	1.270
Cs2g10550	K01180|2.6e-27|cmax:111487562|K01180 endo-1,3(4)-beta-glucanase [EC:3.2.1.6] | (RefSeq) uncharacterized protein LOC111487562	Probable LRR receptor-like serine/threonine-protein kinase At1g56130 OS=Arabidopsis thaliana OX=3702 GN=At1g56130 PE=2 SV=2	1.345
Cs3g27250	K21777|7.0e-46|cit:102615702|K21777 G2/mitotic-specific cyclin-B, other | (RefSeq) cyclin-B2-4	G2/mitotic-specific cyclin-2 (Fragment) OS=Medicago sativa OX=3879 PE=2 SV=1	1.122
Cs5g06200	K08867|2.2e-168|cit:102625534|K08867 WNK lysine deficient protein kinase [EC:2.7.11.1] | (RefSeq) probable serine/threonine-protein kinase WNK3 isoform X1	Probable serine/threonine-protein kinase WNK3 OS=Arabidopsis thaliana OX=3702 GN=WNK3 PE=2 SV=1	1.028
Cs5g22690	K13430|1.6e-63|dzi:111291965|K13430 serine/threonine-protein kinase PBS1 [EC:2.7.11.1] | (RefSeq) serine/threonine-protein kinase PBL27-like isoform X1	Serine/threonine-protein kinase-like protein ACR4 OS=Arabidopsis thaliana OX=3702 GN=ACR4 PE=1 SV=1	1.450
Cs9g06720	K00276|1.0e-185|cic:CICLE_v10004484mg|K00276 primary-amine oxidase [EC:1.4.3.21] | (RefSeq) hypothetical protein	Primary amine oxidase (Fragment) OS=Lens culinaris OX=3864 PE=1 SV=3	-3.393
orange1.1t00405	K13430|2.7e-29|ccaj:109802387|K13430 serine/threonine-protein kinase PBS1 [EC:2.7.11.1] | (RefSeq) serine/threonine-protein kinase PBS1-like isoform X1	Probable serine/threonine-protein kinase At1g01540 OS=Arabidopsis thaliana OX=3702 GN=At1g01540 PE=1 SV=2	1.099
orange1.1t04760	K13420|7.4e-110|cit:102626235|K13420 LRR receptor-like serine/threonine-protein kinase FLS2 [EC:2.7.11.1] | (RefSeq) probable LRR receptor-like serine/threonine-protein kinase At3g47570	Probable LRR receptor-like serine/threonine-protein kinase At3g47570 OS=Arabidopsis thaliana OX=3702 GN=At3g47570 PE=2 SV=1	1.925
Protein dephosphorylation (GO:0006470; adjusted P = 1.0000)			

Cs7g30500	K14496 | 1.2e-114 | cic:CICLE_v10032781mg | K14496 abscisic acid receptor PYR/PYL family | (RefSeq) hypothetical protein	Abscisic acid receptor PYL4 OS=Arabidopsis thaliana OX=3702 GN=PYL4 PE=1 SV=1	-1.525
Protein processing in endoplasmic reticulum (cit04141; corrected P = 0.0066)			
Cs7g32260	K13993 | 1.1e-81 | cit:102617602 | K13993 HSP20 family protein | (RefSeq) 18.1 kDa class I heat shock protein-like	18.2 kDa class I heat shock protein OS=Medicago sativa OX=3879 GN=HSP18.2 PE=2 SV=1	1.504
Cs7g10040	K13993 | 9.2e-84 | cit:102610711 | K13993 HSP20 family protein | (RefSeq) small heat shock protein, chloroplastic-like	Heat shock protein 21, chloroplastic OS=Arabidopsis thaliana OX=3702 GN=HSP21 PE=1 SV=1	-1.139
orange1.1t01933	K03283 | 3.9e-138 | cit:112495491 | K03283 heat shock 70kDa protein 1/2/6/8 | (RefSeq) heat shock 70 kDa protein 6, chloroplastic	Heat shock 70 kDa protein 6, chloroplastic OS=Arabidopsis thaliana OX=3702 GN=HSP70-6 PE=1 SV=1	-1.589
Cs8g18240	K03283 | 3.2e-35 | cic:CICLE_v10028007mg | K03283 heat shock 70kDa protein 1/2/6/8 | (RefSeq) hypothetical protein	Heat shock 70 kDa protein 4 OS=Arabidopsis thaliana OX=3702 GN=HSP70-4 PE=1 SV=1	-1.128
Cs7g28940	K03283 | 0.0e+00 | cic:CICLE_v10033622mg | K03283 heat shock 70kDa protein 1/2/6/8 | (RefSeq) hypothetical protein	Heat shock cognate 70 kDa protein OS=Petunia hybrida OX=4102 GN=HSP70 PE=2 SV=1	-1.727
Cs8g19520	K13993 | 1.1e-86 | cic:CICLE_v10029437mg | K13993 HSP20 family protein | (RefSeq) hypothetical protein	18.2 kDa class I heat shock protein OS=Medicago sativa OX=3879 GN=HSP18.2 PE=2 SV=1	-2.519
Cs2g24360	K13993 | 2.8e-85 | cic:CICLE_v10016547mg | K13993 HSP20 family protein | (RefSeq) hypothetical protein	K13993 | 2.8e-85 | cic:CICLE_v10016547mg | K13993 HSP20 family protein | (RefSeq) hypothetical protein	-2.930
Cs7g23510	K09503 | 6.0e-81 | tcc:18586851 | K09503 DnaJ homolog subfamily A member 2 | (RefSeq) dnaJ protein homolog	DnaJ protein homolog ANJ1 OS=Atriplex nummularia OX=3553 PE=2 SV=1	1.258
Cs4g08220	K04079 | 0.0e+00 | cic:CICLE_v10007633mg | K04079 molecular chaperone HtpG | (RefSeq) hypothetical protein	Heat shock cognate protein 80 OS=Solanum lycopersicum OX=4081 GN=HSC80 PE=2 SV=1	-1.286
Cs2g23310	K06689 | 3.8e-21 | boe:106307910 | K06689 ubiquitin-conjugating enzyme E2 D [EC:2.3.2.23] | (RefSeq) SUMO-conjugating enzyme UBC9	SUMO-conjugating enzyme UBC9 OS=Arabidopsis thaliana OX=3702 GN=UBC9 PE=1 SV=1	1.048
Cs5g03150	K04079 | 7.1e-271 | cit:102616579 | K04079 molecular chaperone HtpG | (RefSeq) heat shock protein 83-like	Heat shock protein 83 OS=Ipomoea nil OX=35883 GN=HSP83A PE=2 SV=1	-1.705
Cs8g15970	K13525 | 3.2e-159 | cit:102612408 | K13525 transitional endoplasmic reticulum ATPase | (RefSeq) cell division cycle protein 48 homolog	Cell division cycle protein 48 homolog OS=Glycine max OX=3847 GN=CDC48 PE=2 SV=1	-1.028
Cs9g19220	K04079 | 5.5e-274 | cic:CICLE_v10004456mg | K04079 molecular chaperone HtpG | (RefSeq) hypothetical protein	Heat shock protein 83 OS=Ipomoea nil OX=35883 GN=HSP83A PE=2 SV=1	-2.036
Cs9g14690	K13993 | 9.0e-71 | cit:102631003 | K13993 HSP20 family protein | (RefSeq) heat shock 22 kDa protein, mitochondrial	Heat shock 22 kDa protein, mitochondrial OS=Pisum sativum OX=3888 GN=HSP22 PE=2 SV=1	-1.931

Cs7g12130	K09487|8.8e-81|cit:102629966|K09487 heat shock protein 90kDa beta | (RefSeq) heat shock protein 90-5, chloroplastic	Heat shock protein 90-5, chloroplastic OS=Arabidopsis thaliana OX=3702 GN=HSP90-5 PE=1 SV=1	-1.570
Cs6g04920	K14015|1.3e-184|cic:CICLE_v10011862mg|K14015 nuclear protein localization protein 4 homolog | (RefSeq) hypothetical protein	NPL4-like protein 1 OS=Arabidopsis thaliana OX=3702 GN=At3g63000 PE=1 SV=1	-1.010
Protein folding (GO:0006457, adjusted P = 0.0003)			
Cs2g23125		(chaperonin CPN60, mitochondrial-like, partial [Citrus clementina])	1.327
Cs2g23190		(chaperonin CPN60, mitochondrial-like isoform X4 [Citrus clementina])	1.158
Cs3g04960	K04043|6.4e-116|cit:102606731|K04043 molecular chaperone DnaK | (RefSeq) heat shock 70 kDa protein, mitochondrial	Heat shock 70 kDa protein, mitochondrial OS=Phaseolus vulgaris OX=3885 PE=2 SV=1	-1.536
Cs3g17640		Chaperone protein dnaJ 1, mitochondrial OS=Arabidopsis thaliana OX=3702 GN=ATJ1 PE=2 SV=2	-1.037
Cs4g07030	K04078|1.6e-62|cic:CICLE_v10009281mg|K04078 chaperonin GroES | (RefSeq) hypothetical protein	20 kDa chaperonin, chloroplastic OS=Arabidopsis thaliana OX=3702 GN=CPN20 PE=1 SV=2	-1.076
Cs4g08220	K04079|0.0e+00|cic:CICLE_v10007633mg|K04079 molecular chaperone HtpG | (RefSeq) hypothetical protein	Heat shock cognate protein 80 OS=Solanum lycopersicum OX=4081 GN=HSC80 PE=2 SV=1	-1.286
Cs5g03150	K04079|7.1e-271|cit:102616579|K04079 molecular chaperone HtpG | (RefSeq) heat shock protein 83-like	Heat shock protein 83 OS=Ipomoea nil OX=35883 GN=HSP83A PE=2 SV=1	-1.705
Cs5g34180	K05864|6.6e-96|cit:102610492|K05864 peptidyl-prolyl isomerase D [EC:5.2.1.8] | (RefSeq) peptidyl-prolyl cis-trans isomerase CYP40	Peptidyl-prolyl cis-trans isomerase CYP40 OS=Arabidopsis thaliana OX=3702 GN=CYP40 PE=2 SV=1	-1.363
Cs6g18810	K09498|7.8e-65|cit:102616990|K09498 T-complex protein 1 subunit zeta | (RefSeq) T-complex protein 1 subunit zeta 1 isoform X1	T-complex protein 1 subunit zeta 1 OS=Arabidopsis thaliana OX=3702 GN=CCT6A PE=1 SV=1	-1.022
Cs7g01450	K03686|1.4e-28|cic:CICLE_v10031530mg|K03686 molecular chaperone DnaJ | (RefSeq) hypothetical protein	Chaperone protein dnaJ GFA2, mitochondrial OS=Arabidopsis thaliana OX=3702 GN=GFA2 PE=2 SV=1	-1.071
Cs7g11140	K03768|9.0e-42|cit:102625552|K03768 peptidyl-prolyl cis-trans isomerase B (cyclophilin B) [EC:5.2.1.8] | (RefSeq) photosynthetic NDH subunit of lumenal location 5, chloroplastic	Photosynthetic NDH subunit of lumenal location 5, chloroplastic OS=Arabidopsis thaliana OX=3702 GN=PNSL5 PE=1 SV=1	-1.910
Cs7g12130	K09487|8.8e-81|cit:102629966|K09487 heat shock protein 90kDa beta | (RefSeq) heat shock protein 90-5, chloroplastic	Heat shock protein 90-5, chloroplastic OS=Arabidopsis thaliana OX=3702 GN=HSP90-5 PE=1 SV=1	-1.570

Cs7g16320	K03687 | 2.3e-89 | cit:102608828 | K03687 molecular chaperone GrpE | (RefSeq) uncharacterized protein LOC102608828 isoform X1	(Uncharacterized protein LOC102608828 isoform X1 [Citrus sinensis])	-1.217
Cs7g16620	K09571 | 2.4e-84 | cic:CICLE_v10031088mg | K09571 FK506-binding protein 4/5 [EC:5.2.1.8] | (RefSeq) hypothetical protein	70 kDa peptidyl-prolyl isomerase OS=Triticum aestivum OX=4565 GN=FKBP70 PE=1 SV=1	-1.225
Cs7g23510	K09503 | 6.0e-81 | tcc:18586851 | K09503 DnaJ homolog subfamily A member 2 | (RefSeq) dnaJ protein homolog	DnaJ protein homolog ANJ1 OS=Atriplex nummularia OX=3553 PE=2 SV=1	1.258
Cs8g03010	K04043 | 8.1e-199 | cit:102615346 | K04043 molecular chaperone DnaK | (RefSeq) heat shock 70 kDa protein, mitochondrial	Heat shock 70 kDa protein, mitochondrial OS=Phaseolus vulgaris OX=3885 PE=2 SV=1	-1.551
Cs8g16040	K04077 | 2.6e-58 | cre:CHLREDRAFT_136288 | K04077 chaperonin GroEL | (RefSeq) CPN60B2; chaperonin 60B2	RuBisCO large subunit-binding protein subunit alpha, chloroplastic OS=Pisum sativum OX=3888 PE=1 SV=2	-1.832
Cs8g19520	K13993 | 1.1e-86 | cic:CICLE_v10029437mg | K13993 HSP20 family protein | (RefSeq) hypothetical protein	18.2 kDa class I heat shock protein OS=Medicago sativa OX=3879 GN=HSP18.2 PE=2 SV=1	-2.519
Cs9g03300	K04077 | 1.6e-133 | cic:CICLE_v10014640mg | K04077 chaperonin GroEL | (RefSeq) hypothetical protein	Chaperonin 60 subunit beta 1, chloroplastic OS=Arabidopsis thaliana OX=3702 GN=CPN60B1 PE=1 SV=3	-1.597
Cs9g19220	K04079 | 5.5e-274 | cic:CICLE_v10004456mg | K04079 molecular chaperone HtpG | (RefSeq) hypothetical protein	Heat shock protein 83 OS=Ipomoea nil OX=35883 GN=HSP83A PE=2 SV=1	-2.036
orange1.1t00006	K09496 | 1.8e-291 | cic:CICLE_v10007963mg | K09496 T-complex protein 1 subunit delta | (RefSeq) hypothetical protein	T-complex protein 1 subunit delta OS=Arabidopsis thaliana OX=3702 GN=CCT4 PE=1 SV=1	-1.079
orange1.1t01459	K04077 | 1.1e-66 | cit:102620413 | K04077 chaperonin GroEL | (RefSeq) chaperonin CPN60-2, mitochondrial	Chaperonin CPN60, mitochondrial OS=Arabidopsis thaliana OX=3702 GN=CPN60 PE=1 SV=2	-1.626
orange1.1t01933	K03283 | 3.9e-138 | cit:112495491 | K03283 heat shock 70kDa protein 1/2/6/8 | (RefSeq) heat shock 70 kDa protein 6, chloroplastic	Heat shock 70 kDa protein 6, chloroplastic OS=Arabidopsis thaliana OX=3702 GN=HSP70-6 PE=1 SV=1	-1.589
Ribosome biogenesis (GO:0042254; adjusted P = 0.0056)			
Cs1g13200	K14558 | 9.9e-260 | cit:102614962 | K14558 periodic tryptophan protein 2 | (RefSeq) periodic tryptophan protein 2	Periodic tryptophan protein 2 OS=Arabidopsis thaliana OX=3702 GN=PWP2 PE=1 SV=1	-1.251
Cs1g16770	K14832 | 7.8e-140 | cit:102626732 | K14832 ribosome biogenesis protein MAK21 | (RefSeq) CCAAT/enhancer-binding protein zeta	(CCAAT/enhancer-binding protein zeta [Citrus sinensis])	-1.367
Cs1g21890		Ribonuclease III domain-containing protein RNC1, chloroplastic OS=Arabidopsis thaliana OX=3702 GN=RNC1 PE=2 SV=1	-1.344

Cs2g09700	K14793 | 8.1e-97 | cit:102629432 | K14793 ribosomal RNA-processing protein 9 | (RefSeq) U3 snoRNP-associated protein-like EMB2271	U3 snoRNP-associated protein-like EMB2271 OS=Arabidopsis thaliana OX=3702 GN=EMB2271 PE=2 SV=1	-1.225
Cs3g21970	K13126 | 6.7e-12 | mtr:MTR_6g040070 | K13126 polyadenylate-binding protein | (RefSeq) DEAD/DEAH-box helicase	DEAD-box ATP-dependent RNA helicase 39 OS=Oryza sativa subsp. japonica OX=39947 GN=Os01g0184500 PE=2 SV=1	-1.173
Cs5g06450	K11294 | 5.7e-112 | cit:102607837 | K11294 nucleolin | (RefSeq) 28 kDa ribonucleoprotein, chloroplastic-like	31 kDa ribonucleoprotein, chloroplastic OS=Arabidopsis thaliana OX=3702 GN=CP31A PE=1 SV=1	-1.136
Cs5g30340	K14553 | 0.0e+00 | cic:CICLE_v10019516mg | K14553 U3 small nucleolar RNA-associated protein 18 | (RefSeq) hypothetical protein	U3 small nucleolar RNA-associated protein 18 homolog OS=Arabidopsis thaliana OX=3702 GN=At5g14050 PE=1 SV=1	-1.304
Cs5g32250	K14549 | 6.2e-296 | cic:CICLE_v10019671mg | K14549 U3 small nucleolar RNA-associated protein 15 | (RefSeq) hypothetical protein	Protein SLOW WALKER 1 OS=Arabidopsis thaliana OX=3702 GN=SWA1 PE=2 SV=1	-1.526
Cs6g16090	K14769 | 3.0e-23 | cit:102608730 | K14769 U3 small nucleolar RNA-associated protein 11 | (RefSeq) probable U3 small nucleolar RNA-associated protein 11	Probable U3 small nucleolar RNA-associated protein 11 OS=Oryza sativa subsp. japonica OX=39947 GN=Os01g0810000 PE=2 SV=2	-1.014
Cs7g08170		(Uncharacterized protein LOC18045706 [Citrus clementina])	-1.016
Cs7g13800	K14563 | 1.2e-81 | cic:CICLE_v10032221mg | K14563 rRNA 2'-O-methyltransferase fibrillarin [EC:2.1.1.-] | (RefSeq) hypothetical protein	rRNA 2'-O-methyltransferase fibrillarin 1 OS=Arabidopsis thaliana OX=3702 GN=FIB1 PE=1 SV=1	-1.182
Cs7g27880	K02864 | 1.1e-111 | cic:CICLE_v10032705mg | K02864 large subunit ribosomal protein L10 | (RefSeq) hypothetical protein	50S ribosomal protein L10, chloroplastic OS=Arabidopsis thaliana OX=3702 GN=RPL10 PE=2 SV=1	-1.101
Cs7g32480	K06941 | 4.3e-45 | cic:CICLE_v10031999mg | K06941 23S rRNA (adenine2503-C2)-methyltransferase [EC:2.1.1.192] | (RefSeq) hypothetical protein	Telomere repeat-binding protein 4 OS=Arabidopsis thaliana OX=3702 GN=TRP4 PE=1 SV=1	-1.149
Cs8g06380	K15032 | 2.0e-81 | cit:102629419 | K15032 mTERF domain-containing protein, mitochondrial | (RefSeq) transcription termination factor MTERF2, chloroplastic	Transcription termination factor MTERF2, chloroplastic OS=Arabidopsis thaliana OX=3702 GN=MTERF2 PE=3 SV=1	-1.062
Cs9g04840	K03979 | 4.8e-43 | mis:MICPUN_78980 | K03979 GTPase [EC:3.6.5.-] | (RefSeq) predicted protein	GTP-binding protein OBGC, chloroplastic OS=Arabidopsis thaliana OX=3702 GN=OBGL PE=2 SV=1	-1.106
Cs9g11490	K06943 | 0.0e+00 | cit:102614112 | K06943 nucleolar GTP-binding protein | (RefSeq) nucleolar GTP-binding protein 1-like	Nucleolar GTP-binding protein 1 OS=Arabidopsis thaliana OX=3702 GN=At1g50920 PE=2 SV=1	-1.193
orange1.1t00409	K02931 | 6.5e-73 | cic:CICLE_v10021696mg | K02931 large subunit ribosomal protein L5 | (RefSeq) hypothetical protein	50S ribosomal protein L5, chloroplastic OS=Arabidopsis thaliana OX=3702 GN=RPL5 PE=2 SV=1	-1.326

orange1.1t02483		RAP domain-containing protein, chloroplastic OS=Arabidopsis thaliana OX=3702 GN=RAP PE=1 SV=1	-1.235
orange1.1t03017	K14556|1.8e-187|cic:CICLE_v10010984mg|K14556 U3 small nucleolar RNA-associated protein 12 | (RefSeq) hypothetical protein	(Hypothetical protein CUMW_233620 [Citrus unshiu])	-1.222
orange1.1t04595	K11108|1.8e-86|cic:CICLE_v10028663mg|K11108 RNA 3'-terminal phosphate cyclase-like protein | (RefSeq) hypothetical protein	Probable RNA 3'-terminal phosphate cyclase-like protein OS=Arabidopsis thaliana OX=3702 GN=At5g22100 PE=2 SV=1	-1.427
Aminoacyl-tRNA biosynthesis (cit00970; corrected P = 0.6633)			
Cs8g01360	K01885|8.0e-87|cit:102612397|K01885 glutamyl-tRNA synthetase [EC:6.1.1.17] | (RefSeq) glutamate--tRNA ligase, chloroplastic/mitochondrial	Glutamate--tRNA ligase, chloroplastic/mitochondrial OS=Arabidopsis thaliana OX=3702 GN=OVA3 PE=1 SV=1	-1.006
Cs8g10730	K01880|1.3e-196|cit:102621048|K01880 glycyl-tRNA synthetase [EC:6.1.1.14] | (RefSeq) glycine--tRNA ligase, mitochondrial 1-like	Glycine--tRNA ligase, mitochondrial 1 OS=Arabidopsis thaliana OX=3702 GN=At1g29880 PE=1 SV=1	-1.088
Cs9g04410	K01870|8.8e-102|cit:102618088|K01870 isoleucyl-tRNA synthetase [EC:6.1.1.5] | (RefSeq) isoleucine--tRNA ligase, chloroplastic/mitochondrial	Isoleucine--tRNA ligase, chloroplastic/mitochondrial OS=Arabidopsis thaliana OX=3702 GN=OVA2 PE=2 SV=1	-1.177
Biosynthesis of amino acids (cit01230; corrected P = 0.0260)			
orange1.1t00226	K00615|1.8e-253|cic:CICLE_v10018991mg|K00615 transketolase [EC:2.2.1.1] | (RefSeq) hypothetical protein	Transketolase, chloroplastic OS=Zea mays OX=4577 PE=1 SV=1	-1.337
Cs3g10480	K01807|1.9e-150|cic:CICLE_v10002135mg|K01807 ribose 5-phosphate isomerase A [EC:5.3.1.6] | (RefSeq) hypothetical protein	Probable ribose-5-phosphate isomerase 3, chloroplastic OS=Arabidopsis thaliana OX=3702 GN=RPI3 PE=1 SV=1	-1.730
Cs5g10920	K01783|3.3e-19|adu:107472214|K01783 ribulose-phosphate 3-epimerase [EC:5.1.3.1] | (RefSeq) ribulose-phosphate 3-epimerase, chloroplastic	Ribulose-5-phosphate-3-epimerase, chloroplastic OS=Arabidopsis thaliana OX=3702 GN=RPE PE=2 SV=1	-1.001
Cs3g17200	K01755|8.0e-182|cit:102618891|K01755 argininosuccinate lyase [EC:4.3.2.1] | (RefSeq) argininosuccinate lyase, chloroplastic	Argininosuccinate lyase, chloroplastic OS=Arabidopsis thaliana OX=3702 GN=At5g10920 PE=2 SV=1	-1.097
Cs3g11490	K01738|3.2e-72|cic:CICLE_v10001793mg|K01738 cysteine synthase [EC:2.5.1.47] | (RefSeq) hypothetical protein	Cysteine synthase, chloroplastic/chromoplastic OS=Solanum tuberosum OX=4113 PE=2 SV=1	-1.300
Cs2g17560	K00133|8.1e-100|cic:CICLE_v10015633mg|K00133 aspartate-semialdehyde dehydrogenase [EC:1.2.1.11] | (RefSeq) hypothetical protein	(Hypothetical protein CISIN_1g017153mg [Citrus sinensis])	-1.064

orange1.1t03280	K00927|9.7e-74|cic:CICLE_v10004891mg|K00927 phosphoglycerate kinase [EC:2.7.2.3] | (RefSeq) hypothetical protein	Phosphoglycerate kinase, chloroplastic OS=Nicotiana tabacum OX=4097 PE=2 SV=1	-1.999
Cs2g13700	K01807|3.2e-144|cit:102627714|K01807 ribose 5-phosphate isomerase A [EC:5.3.1.6] | (RefSeq) probable ribose-5-phosphate isomerase 2	Probable ribose-5-phosphate isomerase 2 OS=Arabidopsis thaliana OX=3702 GN=RPI2 PE=1 SV=1	-1.305
Cs7g30350	K15227|2.6e-32|cic:CICLE_v10032371mg|K15227 arogenate dehydrogenase (NADP+), plant [EC:1.3.1.78] | (RefSeq) hypothetical protein	Arogenate dehydrogenase 1, chloroplastic OS=Arabidopsis thaliana OX=3702 GN=TYRAAT1 PE=1 SV=1	1.887
orange1.1t02000	K01626|2.2e-109|cit:102625514|K01626 3-deoxy-7-phosphoheptulonate synthase [EC:2.5.1.54] | (RefSeq) phospho-2-dehydro-3-deoxyheptonate aldolase 2, chloroplastic-like isoform X1	Phospho-2-dehydro-3-deoxyheptonate aldolase 2, chloroplastic OS=Arabidopsis thaliana OX=3702 GN=DHS2 PE=2 SV=2	-1.215
Cs4g15090	K01733|2.2e-298|cit:102628627|K01733 threonine synthase [EC:4.2.3.1] | (RefSeq) threonine synthase 1, chloroplastic	Threonine synthase 1, chloroplastic OS=Arabidopsis thaliana OX=3702 GN=TS1 PE=1 SV=1	-1.164
Cs5g07120	K01940|1.1e-109|cit:102630538|K01940 argininosuccinate synthase [EC:6.3.4.5] | (RefSeq) argininosuccinate synthase, chloroplastic	Argininosuccinate synthase, chloroplastic OS=Arabidopsis thaliana OX=3702 GN=At4g24830 PE=1 SV=3	-1.032
Cs9g05680	K01915|7.0e-71|cic:CICLE_v10005007mg|K01915 glutamine synthetase [EC:6.3.1.2] | (RefSeq) hypothetical protein	Glutamine synthetase, chloroplastic OS=Daucus carota OX=4039 GN=GLN2 PE=2 SV=1	-1.445
Cs7g16390	K22846|7.4e-82|cic:CICLE_v10031768mg|K22846 S-sulfo-L-cysteine synthase (O-acetyl-L-serine-dependent) [EC:2.5.1.144] | (RefSeq) hypothetical protein	S-sulfo-L-cysteine synthase (O-acetyl-L-serine-dependent), chloroplastic OS=Arabidopsis thaliana OX=3702 GN=CS26 PE=1 SV=1	-2.495
Cellular amino acid catabolic process (GO:0009063; adjusted P = 0.8430)			
Cs2g02890	K00605|5.7e-118|cic:CICLE_v10015433mg|K00605 aminomethyltransferase [EC:2.1.2.10] | (RefSeq) hypothetical protein	Aminomethyltransferase, mitochondrial OS=Solanum tuberosum OX=4113 GN=GDCST PE=2 SV=1	-1.910
Cs4g18520	K00281|5.5e-312|cit:102627653|K00281 glycine dehydrogenase [EC:1.4.4.2] | (RefSeq) glycine dehydrogenase (decarboxylating), mitochondrial	Glycine dehydrogenase (decarboxylating) A, mitochondrial OS=Flaveria pringlei OX=4226 GN=GDCSPA PE=2 SV=1	-1.292
Glycine, serine and threonine metabolism (cit00260; corrected P = 0.0214)			
Cs9g06720	K00276|1.0e-185|cic:CICLE_v10004484mg|K00276 primary-amine oxidase [EC:1.4.3.21] | (RefSeq) hypothetical protein	Primary amine oxidase (Fragment) OS=Lens culinaris OX=3864 PE=1 SV=3	-3.393

Cs9g01670	K00382|3.8e-234|cit:102627107|K00382 dihydrolipoamide dehydrogenase [EC:1.8.1.4] | (RefSeq) dihydrolipoyl dehydrogenase 1, mitochondrial isoform X1	Dihydrolipoyl dehydrogenase 1, mitochondrial OS=Arabidopsis thaliana OX=3702 GN=LPD1 PE=1 SV=2	-2.754
Cs2g17560	K00133|8.1e-100|cic:CICLE_v10015633mg|K00133 aspartate-semialdehyde dehydrogenase [EC:1.2.1.11] | (RefSeq) hypothetical protein	(Hypothetical protein CISIN_1g017153mg [Citrus sinensis])	-1.064
Cs9g06700	K00276|4.5e-99|cic:CICLE_v10006614mg|K00276 primary-amine oxidase [EC:1.4.3.21] | (RefSeq) hypothetical protein	Primary amine oxidase 2 OS=Arabidopsis thaliana OX=3702 GN=At1g31672 PE=3 SV=1	1.927
Cs2g02890	K00605|5.7e-118|cic:CICLE_v10015433mg|K00605 aminomethyltransferase [EC:2.1.2.10] | (RefSeq) hypothetical protein	Aminomethyltransferase, mitochondrial OS=Solanum tuberosum OX=4113 GN=GDCST PE=2 SV=1	-1.910
Cs4g15090	K01733|2.2e-298|cit:102628627|K01733 threonine synthase [EC:4.2.3.1] | (RefSeq) threonine synthase 1, chloroplastic	Threonine synthase 1, chloroplastic OS=Arabidopsis thaliana OX=3702 GN=TS1 PE=1 SV=1	-1.164
Cs4g18520	K00281|5.5e-312|cit:102627653|K00281 glycine dehydrogenase [EC:1.4.4.2] | (RefSeq) glycine dehydrogenase (decarboxylating), mitochondrial	Glycine dehydrogenase (decarboxylating) A, mitochondrial OS=Flaveria pringlei OX=4226 GN=GDCSPA PE=2 SV=1	-1.292
Cs1g15150	K18606|2.3e-85|egr:104449883|K18606 hydroxyphenylpyruvate reductase [EC:1.1.1.237] | (RefSeq) hydroxyphenylpyruvate reductase	Glyoxylate/hydroxypyruvate/pyruvate reductase 2KGR OS=Vitis vinifera OX=29760 GN=2KGR PE=1 SV=1	-1.048
Cysteine and methionine metabolism (cit00270; corrected P = 0.0681)			
Cs3g11490	K01738|3.2e-72|cic:CICLE_v10001793mg|K01738 cysteine synthase [EC:2.5.1.47] | (RefSeq) hypothetical protein	Cysteine synthase, chloroplastic/chromoplastic OS=Solanum tuberosum OX=4113 PE=2 SV=1	-1.300
Cs5g10730	K00026|2.1e-223|cit:102628532|K00026 malate dehydrogenase [EC:1.1.1.37] | (RefSeq) malate dehydrogenase, chloroplastic	Malate dehydrogenase, chloroplastic OS=Arabidopsis thaliana OX=3702 GN=At3g47520 PE=1 SV=1	-1.256
Cs2g17560	K00133|8.1e-100|cic:CICLE_v10015633mg|K00133 aspartate-semialdehyde dehydrogenase [EC:1.2.1.11] | (RefSeq) hypothetical protein	(Hypothetical protein CISIN_1g017153mg [Citrus sinensis])	-1.064
orange1.1t01892	K01251|1.7e-141|cit:102617645|K01251 adenosylhomocysteinase [EC:3.3.1.1] | (RefSeq) adenosylhomocysteinase	Adenosylhomocysteinase OS=Catharanthus roseus OX=4058 GN=SAHH PE=2 SV=1	-1.124
Cs9g04530	K17398|8.7e-08|gmx:100792700|K17398 DNA (cytosine-5)-methyltransferase 3A [EC:2.1.1.37] | (RefSeq) uncharacterized protein LOC100792700	(Hypothetical protein CICLE_v10006926mg [Citrus clementina])	-1.360

Cs7g12410	K01611|9.7e-208|cit:102578065|K01611 S-adenosylmethionine decarboxylase [EC:4.1.1.50] | (RefSeq) SAMDC; S-adenosylmethionine decarboxylase	S-adenosylmethionine decarboxylase proenzyme OS=Nicotiana tabacum OX=4097 GN=SAMDC PE=2 SV=1	-1.653
Cs7g25390	K00026|1.5e-53|cic:CICLE_v10008562mg|K00026 malate dehydrogenase [EC:1.1.1.37] | (RefSeq) hypothetical protein	Malate dehydrogenase, mitochondrial OS=Citrullus lanatus OX=3654 GN=MMDH PE=1 SV=1	-1.555
Cs7g16390	K22846|7.4e-82|cic:CICLE_v10031768mg|K22846 S-sulfo-L-cysteine synthase (O-acetyl-L-serine-dependent) [EC:2.5.1.144] | (RefSeq) hypothetical protein	S-sulfo-L-cysteine synthase (O-acetyl-L-serine-dependent), chloroplastic OS=Arabidopsis thaliana OX=3702 GN=CS26 PE=1 SV=1	-2.495
Alanine, aspartate and glutamate metabolism (cit00250; corrected P = 0.0681)			
Cs8g07660	K00764|7.6e-168|cit:102622004|K00764 amidophosphoribosyltransferase [EC:2.4.2.14] | (RefSeq) LOW QUALITY PROTEIN: amidophosphoribosyltransferase, chloroplastic-like	Amidophosphoribosyltransferase 2, chloroplastic OS=Arabidopsis thaliana OX=3702 GN=ASE2 PE=1 SV=1	-1.418
Cs8g17170	K01955|0.0e+00|cic:CICLE_v10027703mg|K01955 carbamoyl-phosphate synthase large subunit [EC:6.3.5.5] | (RefSeq) hypothetical protein	Carbamoyl-phosphate synthase large chain, chloroplastic OS=Arabidopsis thaliana OX=3702 GN=CARB PE=1 SV=1	-1.345
Cs5g07120	K01940|1.1e-109|cit:102630538|K01940 argininosuccinate synthase [EC:6.3.4.5] | (RefSeq) argininosuccinate synthase, chloroplastic	Argininosuccinate synthase, chloroplastic OS=Arabidopsis thaliana OX=3702 GN=At4g24830 PE=1 SV=3	-1.032
Cs3g17200	K01755|8.0e-182|cit:102618891|K01755 argininosuccinate lyase [EC:4.3.2.1] | (RefSeq) argininosuccinate lyase, chloroplastic	Argininosuccinate lyase, chloroplastic OS=Arabidopsis thaliana OX=3702 GN=At5g10920 PE=2 SV=1	-1.097
Cs9g05680	K01915|7.0e-71|cic:CICLE_v10005007mg|K01915 glutamine synthetase [EC:6.3.1.2] | (RefSeq) hypothetical protein	Glutamine synthetase, chloroplastic OS=Daucus carota OX=4039 GN=GLN2 PE=2 SV=1	-1.445
Arginine biosynthesis (cit00220; corrected P = 0.2476)			
Cs9g05680	K01915|7.0e-71|cic:CICLE_v10005007mg|K01915 glutamine synthetase [EC:6.3.1.2] | (RefSeq) hypothetical protein	Glutamine synthetase, chloroplastic OS=Daucus carota OX=4039 GN=GLN2 PE=2 SV=1	-1.445
Cs5g07120	K01940|1.1e-109|cit:102630538|K01940 argininosuccinate synthase [EC:6.3.4.5] | (RefSeq) argininosuccinate synthase, chloroplastic	Argininosuccinate synthase, chloroplastic OS=Arabidopsis thaliana OX=3702 GN=At4g24830 PE=1 SV=3	-1.032
Cs3g17200	K01755|8.0e-182|cit:102618891|K01755 argininosuccinate lyase [EC:4.3.2.1] | (RefSeq) argininosuccinate lyase, chloroplastic	Argininosuccinate lyase, chloroplastic OS=Arabidopsis thaliana OX=3702 GN=At5g10920 PE=2 SV=1	-1.097

Tyrosine metabolism(cit00350; corrected P = 0.3519)			
Cs9g06720	K00276|1.0e-185|cic:CICLE_v10004484mg|K00276 primary-amine oxidase [EC:1.4.3.21] | (RefSeq) hypothetical protein	Primary amine oxidase (Fragment) OS=Lens culinaris OX=3864 PE=1 SV=3	-3.393
Cs9g06700	K00276|4.5e-99|cic:CICLE_v10006614mg|K00276 primary-amine oxidase [EC:1.4.3.21] | (RefSeq) hypothetical protein	Primary amine oxidase 2 OS=Arabidopsis thaliana OX=3702 GN=At1g31672 PE=3 SV=1	1.927
Cs3g01240	K01800|1.4e-22|cic:CICLE_v10002464mg|K01800 maleylacetoacetate isomerase [EC:5.2.1.2] | (RefSeq) hypothetical protein	Glutathione S-transferase zeta class OS=Euphorbia esula OX=3993 PE=2 SV=1	1.712
Cs7g14430	K00121|1.7e-106|cit:102622247|K00121 S-(hydroxymethyl)glutathione dehydrogenase / alcohol dehydrogenase [EC:1.1.1.284 1.1.1.1] | (RefSeq) alcohol dehydrogenase-like 2 isoform X1	CYP enzymes assisting alcohol dehydrogenase OS=Catharanthus roseus OX=4058 GN=CYPADH PE=1 SV=1	1.605
Lysine biosynthesis(cit00300; corrected P = 0.5905)			
Cs2g17560	K00133|0|cit:102608194|aspartate-semialdehyde dehydrogenase-like; K00133 aspartate-semialdehyde dehydrogenase [EC:1.2.1.11] (A)	(Hypothetical protein CISIN_1g017153mg [Citrus sinensis])	-1.064
Phenylalanine, tyrosine and tryptophan biosynthesis (cit00400; corrected P = 0.6634)			
orange1.1t02000	K01626|2.2e-109|cit:102625514|K01626 3-deoxy-7-phosphoheptulonate synthase [EC:2.5.1.54] | (RefSeq) phospho-2-dehydro-3-deoxyheptonate aldolase 2, chloroplastic-like isoform X1	Phospho-2-dehydro-3-deoxyheptonate aldolase 2, chloroplastic OS=Arabidopsis thaliana OX=3702 GN=DHS2 PE=2 SV=2	-1.215
Cs7g30350	K15227|2.6e-32|cic:CICLE_v10032371mg|K15227 arogenate dehydrogenase (NADP+), plant [EC:1.3.1.78] | (RefSeq) hypothetical protein	Arogenate dehydrogenase 1, chloroplastic OS=Arabidopsis thaliana OX=3702 GN=TYRAAT1 PE=1 SV=1	1.887
Phenylalanine metabolism (cit00360; corrected P = 0.6634)			
Cs9g06720	K00276|1.0e-185|cic:CICLE_v10004484mg|K00276 primary-amine oxidase [EC:1.4.3.21] | (RefSeq) hypothetical protein	Primary amine oxidase (Fragment) OS=Lens culinaris OX=3864 PE=1 SV=3	-3.393
Cs9g06700	K00276|4.5e-99|cic:CICLE_v10006614mg|K00276 primary-amine oxidase [EC:1.4.3.21] | (RefSeq) hypothetical protein	Primary amine oxidase 2 OS=Arabidopsis thaliana OX=3702 GN=At1g31672 PE=3 SV=1	1.927
Histidine metabolism (cit00340; corrected P =0.6634)			

Cs3g19820	K00128|2.9e-44|cit:102611460|K00128 aldehyde dehydrogenase (NAD+) [EC:1.2.1.3] | (RefSeq) aldehyde dehydrogenase family 3 member H1	Aldehyde dehydrogenase family 3 member H1 OS=Arabidopsis thaliana OX=3702 GN=ALDH3H1 PE=1 SV=2	-1.055
Tryptophan metabolism (cit00380; corrected P = 0.6634)			
Cs3g19820	K00128|2.9e-44|cit:102611460|K00128 aldehyde dehydrogenase (NAD+) [EC:1.2.1.3] | (RefSeq) aldehyde dehydrogenase family 3 member H1	Aldehyde dehydrogenase family 3 member H1 OS=Arabidopsis thaliana OX=3702 GN=ALDH3H1 PE=1 SV=2	-1.055
Cs9g01670	K00382|3.8e-234|cit:102627107|K00382 dihydrolipoamide dehydrogenase [EC:1.8.1.4] | (RefSeq) dihydrolipoyl dehydrogenase 1, mitochondrial isoform X1	Dihydrolipoyl dehydrogenase 1, mitochondrial OS=Arabidopsis thaliana OX=3702 GN=LPD1 PE=1 SV=2	-2.754
Arginine and proline metabolism (cit00330; corrected P = 0.6784)			
Cs7g12410	K01611|9.7e-208|cit:102578065|K01611 S-adenosylmethionine decarboxylase [EC:4.1.1.50] | (RefSeq) SAMDC; S-adenosylmethionine decarboxylase	S-adenosylmethionine decarboxylase proenzyme OS=Nicotiana tabacum OX=4097 GN=SAMDC PE=2 SV=1	-1.653
Cs3g19820	K00128|2.9e-44|cit:102611460|K00128 aldehyde dehydrogenase (NAD+) [EC:1.2.1.3] | (RefSeq) aldehyde dehydrogenase family 3 member H1	Aldehyde dehydrogenase family 3 member H1 OS=Arabidopsis thaliana OX=3702 GN=ALDH3H1 PE=1 SV=2	-1.055

Table S10. Low phosphate-responsive genes in low pH-treated *Citrus sinensis* leaves

Accession No.	KEGG_annotation	Swissprot_annotation (NR_annotation)	Log_2(fold change)
Organophosphate biosynthetic process (GO:0090407; adjusted P = 0.3825)			
Cs1g12410		Phosphatidylcholine:diacylglycerol cholinephosphotransferase 1 OS=Arabidopsis thaliana OX=3702 GN=ROD1 PE=1 SV=1	1.449
Cs1g14970	K06215|3.8e-168|cit:102610004|K06215 pyridoxal 5'-phosphate synthase pdxS subunit [EC:4.3.3.6] | (RefSeq) pyridoxal 5'-phosphate synthase-like subunit PDX1.2	Pyridoxal 5'-phosphate synthase-like subunit PDX1.2 OS=Arabidopsis thaliana OX=3702 GN=PDX12 PE=1 SV=1	-1.362
Cs2g03080	K02115|4.3e-195|cit:102629991|K02115 F-type H+-transporting ATPase subunit gamma | (RefSeq) ATP synthase gamma chain, chloroplastic	ATP synthase gamma chain, chloroplastic OS=Nicotiana tabacum OX=4097 GN=ATPC PE=1 SV=1	-1.309
Cs2g11760	K17807|2.9e-41|cit:102607687|K17807 mitochondrial translocator assembly and maintenance protein 41 | (RefSeq) phosphatidate cytidylyltransferase, mitochondrial isoform X1	(Hypothetical protein CISIN_1g037930mg, partial [Citrus sinensis])	1.122
Cs5g03510	K01951|1.9e-120|cic:CICLE_v10019676mg|K01951 GMP synthase (glutamine-hydrolysing) [EC:6.3.5.2] | (RefSeq) hypothetical protein	(Hypothetical protein CUMW_041950 [Citrus unshiu])	-1.355
Cs5g05515	K09680|2.6e-54|cit:102629945|K09680 type II pantothenate kinase [EC:2.7.1.33] | (RefSeq) pantothenate kinase 2	Pantothenate kinase 2 OS=Arabidopsis thaliana OX=3702 GN=PANK2 PE=1 SV=2	-1.247
Cs5g10740	K10960|3.6e-141|cic:CICLE_v10020061mg|K10960 geranylgeranyl diphosphate/geranylgeranyl-bacteriochlorophyllide a reductase [EC:1.3.1.83 1.3.1.111] | (RefSeq) hypothetical protein	Geranylgeranyl diphosphate reductase, chloroplastic OS=Arabidopsis thaliana OX=3702 GN=CHLP PE=1 SV=1	-1.716
Cs5g19690	K11808|1.6e-06|qsu:111985940|K11808 phosphoribosylaminoimidazole carboxylase [EC:4.1.1.21] | (RefSeq) phosphoribosylaminoimidazole carboxylase, chloroplastic-like isoform X1	(Hypothetical protein CUMW_218330 [Citrus unshiu])	-2.035
Cs5g28200	K03527|2.1e-60|cit:102610997|K03527 4-hydroxy-3-methylbut-2-en-1-yl diphosphate reductase [EC:1.17.7.4] | (RefSeq) 4-hydroxy-3-methylbut-2-enyl diphosphate reductase, chloroplastic-like isoform X1	4-hydroxy-3-methylbut-2-enyl diphosphate reductase, chloroplastic OS=Arabidopsis thaliana OX=3702 GN=ISPH PE=1 SV=1	-1.121
Cs8g07660	K00764|7.6e-168|cit:102622004|K00764 amidophosphoribosyltransferase [EC:2.4.2.14] | (RefSeq) LOW QUALITY PROTEIN: amidophosphoribosyltransferase, chloroplastic-like	Amidophosphoribosyltransferase 2, chloroplastic OS=Arabidopsis thaliana OX=3702 GN=ASE2 PE=1 SV=1	-1.418
Cs8g10730	K01880|1.3e-196|cit:102621048|K01880 glycyl-tRNA synthetase [EC:6.1.1.14] | (RefSeq) glycine--tRNA ligase, mitochondrial 1-like	Glycine--tRNA ligase, mitochondrial 1 OS=Arabidopsis thaliana OX=3702 GN=At1g29880 PE=1 SV=1	-1.088

Cs8g13200	K11808|5.1e-73|cit:102626933|K11808 phosphoribosylaminoimidazole carboxylase [EC:4.1.1.21] | (RefSeq) phosphoribosylaminoimidazole carboxylase, chloroplastic isoform X1	Phosphoribosylaminoimidazole carboxylase, chloroplastic (Fragment) OS=Vigna aconitifolia OX=3918 GN=PURKE PE=2 SV=1	-1.164
Cs8g20230	K06215|4.4e-175|cic:CICLE_v10028683mg|K06215 pyridoxal 5'-phosphate synthase pdxS subunit [EC:4.3.3.6] | (RefSeq) hypothetical protein	Probable pyridoxal 5'-phosphate synthase subunit PDX1 OS=Hevea brasiliensis OX=3981 GN=PDX1 PE=2 SV=1	-1.356
Organophosphate catabolic process (GO:0046434; adjusted P = 0.9655)			
Cs7g02950	K18696|2.5e-115|cic:CICLE_v10031648mg|K18696 glycerophosphodiester phosphodiesterase [EC:3.1.4.46] | (RefSeq) hypothetical protein	Glycerophosphodiester phosphodiesterase GDPD3 OS=Arabidopsis thaliana OX=3702 GN=GDPD3 PE=2 SV=1	1.948
Phospholipid catabolic process (GO:0009395; adjusted P = 0.6382)			
Cs7g02950	K18696|2.5e-115|cic:CICLE_v10031648mg|K18696 glycerophosphodiester phosphodiesterase [EC:3.1.4.46] | (RefSeq) hypothetical protein	Glycerophosphodiester phosphodiesterase GDPD3 OS=Arabidopsis thaliana OX=3702 GN=GDPD3 PE=2 SV=1	1.948
Phospholipid biosynthetic process (GO:0008654; adjusted P = 0.7591)			
Cs1g12410		Phosphatidylcholine:diacylglycerol cholinephosphotransferase 1 OS=Arabidopsis thaliana OX=3702 GN=ROD1 PE=1 SV=1	1.449
Cs2g11760	K17807|2.9e-41|cit:102607687|K17807 mitochondrial translocator assembly and maintenance protein 41 | (RefSeq) phosphatidate cytidylyltransferase, mitochondrial isoform X1	(Hypothetical protein CISIN_1g037930mg, partial [Citrus sinensis])	1.122
Cs5g10740	K10960|3.6e-141|cic:CICLE_v10020061mg|K10960 geranylgeranyl diphosphate/geranylgeranyl-bacteriochlorophyllide a reductase [EC:1.3.1.83 1.3.1.111] | (RefSeq) hypothetical protein	Geranylgeranyl diphosphate reductase, chloroplastic OS=Arabidopsis thaliana OX=3702 GN=CHLP PE=1 SV=1	-1.716
Cs5g28200	K03527|2.1e-60|cit:102610997|K03527 4-hydroxy-3-methylbut-2-en-1-yl diphosphate reductase [EC:1.17.7.4] | (RefSeq) 4-hydroxy-3-methylbut-2-enyl diphosphate reductase, chloroplastic-like isoform X1	4-hydroxy-3-methylbut-2-enyl diphosphate reductase, chloroplastic OS=Arabidopsis thaliana OX=3702 GN=ISPH PE=1 SV=1	-1.121
Glycerolipid catabolic process (GO:0046503; adjusted P = 0.3091)			
Cs7g02950	K18696|2.5e-115|cic:CICLE_v10031648mg|K18696 glycerophosphodiester phosphodiesterase [EC:3.1.4.46] | (RefSeq) hypothetical protein	Glycerophosphodiester phosphodiesterase GDPD3 OS=Arabidopsis thaliana OX=3702 GN=GDPD3 PE=2 SV=1	1.948
Glycerolipid biosynthetic process (GO:0045017, adjusted P = 0.8568)			
Cs1g12410		Phosphatidylcholine:diacylglycerol cholinephosphotransferase 1 OS=Arabidopsis thaliana OX=3702 GN=ROD1 PE=1 SV=1	1.449

Cs2g11760	K17807|2.9e-41|cit:102607687|K17807 mitochondrial translocator assembly and maintenance protein 41 | (RefSeq) phosphatidate cytidylyltransferase, mitochondrial isoform X1	(Hypothetical protein CISIN_1g037930mg, partial [Citrus sinensis])	1.122
Cs2g23350	K20098|4.7e-51|mtr:MTR_4g078460|K20098 DNA excision repair protein ERCC-6-like 2 [EC:3.6.4.-] | (RefSeq) DNA repair and recombination RAD26-like protein, putative	O-acyltransferase WSD1 OS=Arabidopsis thaliana OX=3702 GN=WSD1 PE=2 SV=1	1.830
Cellular response to phosphate starvation (GO:0016036; adjusted P = 0.3682)			
Cs1g18540	K13248|3.8e-85|cic:CICLE_v10026096mg|K13248 pyridoxal phosphate phosphatase PHOSPHO2 [EC:3.1.3.74] | (RefSeq) hypothetical protein	Inorganic pyrophosphatase 1 OS=Arabidopsis thaliana OX=3702 GN=PS2 PE=1 SV=1	3.003
Cs1g20920	K01610|1.9e-101|cit:102629129|K01610 phosphoenolpyruvate carboxykinase (ATP) [EC:4.1.1.49] | (RefSeq) phosphoenolpyruvate carboxykinase (ATP)-like	Phosphoenolpyruvate carboxykinase (ATP) 1 OS=Arabidopsis thaliana OX=3702 GN=PCK1 PE=1 SV=1	1.058
Cs4g17870		SPX domain-containing protein 2 OS=Arabidopsis thaliana OX=3702 GN=SPX2 PE=2 SV=1	1.638
Phosphate ion homeostasis (GO:0055062; adjusted P = 0.3530)			
Cs6g08320	K07300|1.9e-124|cic:CICLE_v10011720mg|K07300 Ca2+:H+ antiporter | (RefSeq) hypothetical protein	Vacuolar cation/proton exchanger 3 OS=Arabidopsis thaliana OX=3702 GN=CAX3 PE=1 SV=1	-2.683
Cs7g02950	K18696|2.5e-115|cic:CICLE_v10031648mg|K18696 glycerophosphodiester phosphodiesterase [EC:3.1.4.46] | (RefSeq) hypothetical protein	Glycerophosphodiester phosphodiesterase GDPD3 OS=Arabidopsis thaliana OX=3702 GN=GDPD3 PE=2 SV=1	1.948
Inorganic phosphate transmembrane transporter activity (GO:0005315; adjusted = 0.2413)			
Cs4g04420	K02045|1.2e-13|cvr:CHLNCDRAFT_143202|K02045 sulfate/thiosulfate transport system ATP-binding protein [EC:7.3.2.3] | (RefSeq) hypothetical protein	ABC transporter I family member 17 OS=Arabidopsis thaliana OX=3702 GN=ABCI17 PE=2 SV=1	1.536
Cs8g19980	K08193|1.8e-133|cic:CICLE_v10028231mg|K08193 MFS transporter, ACS family, solute carrier family 17 (sodium-dependent inorganic phosphate cotransporter), other | (RefSeq) hypothetical protein	Sodium-dependent phosphate transport protein 1, chloroplastic OS=Arabidopsis thaliana OX=3702 GN=ANTR1 PE=1 SV=1	-1.225
Others			
Cs2g17940	K14379|4.2e-71|cit:102617538|K14379 tartrate-resistant acid phosphatase type 5 [EC:3.1.3.2] | (RefSeq) purple acid phosphatase 4	Purple acid phosphatase 8 OS=Arabidopsis thaliana OX=3702 GN=PAP8 PE=2 SV=1	2.494
Cs2g18380	K01166|7.9e-45|cit:102616155|K01166 ribonuclease T2 [EC:4.6.1.19] | (RefSeq) ribonuclease 2	Ribonuclease 2 OS=Arabidopsis thaliana OX=3702 GN=RNS2 PE=2 SV=1	1.289

Table S11. Differentially expressed genes (DEGs) related to secondary metabolism in low pH-treated *Citrus sinensis* leaves

Accession No.	KEGG_annotation	Swissprot_annotation (NR_annotation)	Log_2(fold change)
Biosynthesis of secondary metabolites (cit01110; corrected P = 0.0008)			
orange1.1t00226	K00615|1.8e-253|cic:CICLE_v10018991mg|K00615 transketolase [EC:2.2.1.1] | (RefSeq) hypothetical protein	Transketolase, chloroplastic OS=Zea mays OX=4577 PE=1 SV=1	-1.337
Cs7g11110	K00036|2.2e-114|cit:102624333|K00036 glucose-6-phosphate 1-dehydrogenase [EC:1.1.1.49 1.1.1.363] | (RefSeq) glucose-6-phosphate 1-dehydrogenase, chloroplastic isoform X1	Glucose-6-phosphate 1-dehydrogenase, chloroplastic OS=Nicotiana tabacum OX=4097 PE=2 SV=1	-1.151
Cs7g14430	K00121|1.7e-106|cit:102622247|K00121 S-(hydroxymethyl)glutathione dehydrogenase / alcohol dehydrogenase [EC:1.1.1.284 1.1.1.1] | (RefSeq) alcohol dehydrogenase-like 2 isoform X1	CYP enzymes assisting alcohol dehydrogenase OS=Catharanthus roseus OX=4058 GN=CYPADH PE=1 SV=1	1.605
Cs8g13200	K11808|5.1e-73|cit:102626933|K11808 phosphoribosylaminoimidazole carboxylase [EC:4.1.1.21] | (RefSeq) phosphoribosylaminoimidazole carboxylase, chloroplastic isoform X1	Phosphoribosylaminoimidazole carboxylase, chloroplastic (Fragment) OS=Vigna aconitifolia OX=3918 GN=PURKE PE=2 SV=1	-1.164
Cs7g30350	K15227|2.6e-32|cic:CICLE_v10032371mg|K15227 arogenate dehydrogenase (NADP+), plant [EC:1.3.1.78] | (RefSeq) hypothetical protein	Arogenate dehydrogenase 1, chloroplastic OS=Arabidopsis thaliana OX=3702 GN=TYRAAT1 PE=1 SV=1	1.887
Cs9g04920	K19073|8.5e-235|cit:102611623|K19073 divinyl chlorophyllide a 8-vinyl-reductase [EC:1.3.1.75] | (RefSeq) divinyl chlorophyllide a 8-vinyl-reductase, chloroplastic	Divinyl chlorophyllide a 8-vinyl-reductase, chloroplastic OS=Cucumis sativus OX=3659 GN=DVR PE=1 SV=1	-1.624
Cs4g02360	K13065|8.6e-109|cit:102628720|K13065 shikimate O-hydroxycinnamoyltransferase [EC:2.3.1.133] | (RefSeq) shikimate O-hydroxycinnamoyltransferase-like	Shikimate O-hydroxycinnamoyltransferase OS=Nicotiana tabacum OX=4097 GN=HST PE=1 SV=1	-1.482
Cs4g18520	K00281|5.5e-312|cit:102627653|K00281 glycine dehydrogenase [EC:1.4.4.2] | (RefSeq) glycine dehydrogenase (decarboxylating), mitochondrial	Glycine dehydrogenase (decarboxylating) A, mitochondrial OS=Flaveria pringlei OX=4226 GN=GDCSPA PE=2 SV=1	-1.292
Cs3g11180	K00514|7.0e-92|cit:102577969|K00514 zeta-carotene desaturase [EC:1.3.5.6] | (RefSeq) ZDS	Zeta-carotene desaturase, chloroplastic/chromoplastic OS=Capsicum annuum OX=4072 GN=ZDS PE=1 SV=1	-1.675
orange1.1t02947	K13066|1.8e-102|cit:102620533|K13066 caffeic acid 3-O-methyltransferase [EC:2.1.1.68] | (RefSeq) anthranilate N-methyltransferase-like	Flavone 3'-O-methyltransferase 1 OS=Arabidopsis thaliana OX=3702 GN=OMT1 PE=1 SV=1	-3.099

Cs1g06850	K00218|5.4e-124|cit:102614959|K00218 protochlorophyllide reductase [EC:1.3.1.33] | (RefSeq) protochlorophyllide reductase, chloroplastic	Protochlorophyllide reductase, chloroplastic OS=Cucumis sativus OX=3659 GN=PORA PE=2 SV=1	-1.307
Cs2g02890	K00605|5.7e-118|cic:CICLE_v10015433mg|K00605 aminomethyltransferase [EC:2.1.2.10] | (RefSeq) hypothetical protein	Aminomethyltransferase, mitochondrial OS=Solanum tuberosum OX=4113 GN=GDCST PE=2 SV=1	-1.910
Cs5g11730	K05280|1.8e-162|cit:102620909|K05280 flavonoid 3'-monooxygenase [EC:1.14.14.82] | (RefSeq) flavonoid 3'-monooxygenase	Flavonoid 3'-monooxygenase OS=Petunia hybrida OX=4102 GN=CYP75B2 PE=2 SV=1	-3.552
orange1.1t03280	K00927|9.7e-74|cic:CICLE_v10004891mg|K00927 phosphoglycerate kinase [EC:2.7.2.3] | (RefSeq) hypothetical protein	Phosphoglycerate kinase, chloroplastic OS=Nicotiana tabacum OX=4097 PE=2 SV=1	-1.999
Cs7g25390	K00026|1.5e-53|cic:CICLE_v10008562mg|K00026 malate dehydrogenase [EC:1.1.1.37] | (RefSeq) hypothetical protein	Malate dehydrogenase, mitochondrial OS=Citrullus lanatus OX=3654 GN=MMDH PE=1 SV=1	-1.555
Cs3g04170	K15096|2.7e-244|cit:102607029|K15096 (R)-limonene synthase [EC:4.2.3.20] | (RefSeq) (R)-limonene synthase 1, chloroplastic	(R)-limonene synthase 1, chloroplastic OS=Citrus sinensis OX=2711 PE=1 SV=2	2.811
Cs8g07660	K00764|7.6e-168|cit:102622004|K00764 amidophosphoribosyltransferase [EC:2.4.2.14] | (RefSeq) LOW QUALITY PROTEIN: amidophosphoribosyltransferase, chloroplastic-like	Amidophosphoribosyltransferase 2, chloroplastic OS=Arabidopsis thaliana OX=3702 GN=ASE2 PE=1 SV=1	-1.418
Cs4g15090	K01733|2.2e-298|cit:102628627|K01733 threonine synthase [EC:4.2.3.1] | (RefSeq) threonine synthase 1, chloroplastic	Threonine synthase 1, chloroplastic OS=Arabidopsis thaliana OX=3702 GN=TS1 PE=1 SV=1	-1.164
Cs1g24440	K21483|5.6e-135|cit:102613997|K21483 salicylate 1-O-methyltransferase [EC:2.1.1.274] | (RefSeq) salicylate carboxymethyltransferase	Salicylate carboxymethyltransferase OS=Clarkia breweri OX=36903 GN=SAMT PE=1 SV=1	-3.138
Cs6g21370	K00703|1.0e-258|cit:102614846|K00703 starch synthase [EC:2.4.1.21] | (RefSeq) granule-bound starch synthase 2, chloroplastic/amyloplastic	Granule-bound starch synthase 2, chloroplastic/amyloplastic OS=Solanum tuberosum OX=4113 GN=SS2 PE=1 SV=3	-1.754
Cs1g15150	K18606|2.3e-85|egr:104449883|K18606 hydroxyphenylpyruvate reductase [EC:1.1.1.237] | (RefSeq) hydroxyphenylpyruvate reductase	Glyoxylate/hydroxypyruvate/pyruvate reductase 2KGR OS=Vitis vinifera OX=29760 GN=2KGR PE=1 SV=1	-1.048
Cs7g13850	K01749|3.5e-93|cic:CICLE_v10031858mg|K01749 hydroxymethylbilane synthase [EC:2.5.1.61] | (RefSeq) hypothetical protein	Porphobilinogen deaminase, chloroplastic OS=Pisum sativum OX=3888 GN=HEMC PE=1 SV=1	-1.414
Cs3g19770	K21604|3.0e-71|cit:102608592|K21604 jasmonoyl-L-amino acid hydrolase [EC:3.5.1.127] | (RefSeq) IAA-amino acid hydrolase ILR1-like 6	IAA-amino acid hydrolase ILR1-like 6 OS=Oryza sativa subsp. japonica OX=39947 GN=ILL6 PE=2 SV=1	-1.120

Cs9g06360	K00975|2.1e-69|cic:CICLE_v10004731mg|K00975 glucose-1-phosphate adenylyltransferase [EC:2.7.7.27] | (RefSeq) hypothetical protein	Glucose-1-phosphate adenylyltransferase large subunit 1, chloroplastic OS=Arabidopsis thaliana OX=3702 GN=ADG2 PE=1 SV=3	-1.206
Cs2g17050	K01835|1.1e-16|cic:CICLE_v10014721mg|K01835 phosphoglucomutase [EC:5.4.2.2] | (RefSeq) hypothetical protein	Probable phosphoglucomutase, cytoplasmic 1 OS=Arabidopsis thaliana OX=3702 GN=At1g23190 PE=2 SV=2	-1.761
Cs7g16390	K22846|7.4e-82|cic:CICLE_v10031768mg|K22846 S-sulfo-L-cysteine synthase (O-acetyl-L-serine-dependent) [EC:2.5.1.144] | (RefSeq) hypothetical protein	S-sulfo-L-cysteine synthase (O-acetyl-L-serine-dependent), chloroplastic OS=Arabidopsis thaliana OX=3702 GN=CS26 PE=1 SV=1	-2.495
Cs3g10480	K01807|1.9e-150|cic:CICLE_v10002135mg|K01807 ribose 5-phosphate isomerase A [EC:5.3.1.6] | (RefSeq) hypothetical protein	Probable ribose-5-phosphate isomerase 3, chloroplastic OS=Arabidopsis thaliana OX=3702 GN=RPI3 PE=1 SV=1	-1.730
Cs9g06720	K00276|1.0e-185|cic:CICLE_v10004484mg|K00276 primary-amine oxidase [EC:1.4.3.21] | (RefSeq) hypothetical protein	Primary amine oxidase (Fragment) OS=Lens culinaris OX=3864 PE=1 SV=3	-3.393
Cs3g17200	K01755|8.0e-182|cit:102618891|K01755 argininosuccinate lyase [EC:4.3.2.1] | (RefSeq) argininosuccinate lyase, chloroplastic	Argininosuccinate lyase, chloroplastic OS=Arabidopsis thaliana OX=3702 GN=At5g10920 PE=2 SV=1	-1.097
Cs1g19540	K00430|8.3e-83|cit:102611617|K00430 peroxidase [EC:1.11.1.7] | (RefSeq) peroxidase 47	Peroxidase 47 OS=Arabidopsis thaliana OX=3702 GN=PER47 PE=2 SV=2	1.667
Cs5g10730	K00026|2.1e-223|cit:102628532|K00026 malate dehydrogenase [EC:1.1.1.37] | (RefSeq) malate dehydrogenase, chloroplastic	Malate dehydrogenase, chloroplastic OS=Arabidopsis thaliana OX=3702 GN=At3g47520 PE=1 SV=1	-1.256
Cs5g26080	K09839|2.5e-150|cic:CICLE_v10019925mg|K09839 violaxanthin de-epoxidase [EC:1.23.5.1] | (RefSeq) hypothetical protein	Violaxanthin de-epoxidase, chloroplastic OS=Spinacia oleracea OX=3562 GN=VDE1 PE=1 SV=2	-1.246
Cs7g22830	K04123|6.5e-134|cic:CICLE_v10031341mg|K04123 ent-kaurenoic acid monooxygenase [EC:1.14.14.107] | (RefSeq) hypothetical protein	Ent-kaurenoic acid oxidase 2 OS=Arabidopsis thaliana OX=3702 GN=KAO2 PE=1 SV=2	1.318
Cs3g16730	K02492|4.2e-206|cit:102624392|K02492 glutamyl-tRNA reductase [EC:1.2.1.70] | (RefSeq) glutamyl-tRNA reductase 1, chloroplastic	Glutamyl-tRNA reductase 1, chloroplastic OS=Cucumis sativus OX=3659 GN=HEMA1 PE=2 SV=1	-1.277
Cs5g10740	K10960|3.6e-141|cic:CICLE_v10020061mg|K10960 geranylgeranyl diphosphate/geranylgeranyl-bacteriochlorophyllide a reductase [EC:1.3.1.83 1.3.1.111] | (RefSeq) hypothetical protein	Geranylgeranyl diphosphate reductase, chloroplastic OS=Arabidopsis thaliana OX=3702 GN=CHLP PE=1 SV=1	-1.716
orange1.1t02000	K01626|2.2e-109|cit:102625514|K01626 3-deoxy-7-phosphoheptulonate synthase [EC:2.5.1.54] | (RefSeq) phospho-2-dehydro-3-deoxyheptonate aldolase 2, chloroplastic-like isoform X1	Phospho-2-dehydro-3-deoxyheptonate aldolase 2, chloroplastic OS=Arabidopsis thaliana OX=3702 GN=DHS2 PE=2 SV=2	-1.215
Cs7g01350	K01188|1.0e-74|cic:CICLE_v10031209mg|K01188 beta-glucosidase [EC:3.2.1.21] | (RefSeq) hypothetical protein	Beta-glucosidase 13 OS=Oryza sativa subsp. japonica OX=39947 GN=BGLU13 PE=2 SV=2	-1.095

Cs7g13170	K00627|2.3e-167|cit:102609118|K00627 pyruvate dehydrogenase E2 component (dihydrolipoamide acetyltransferase) [EC:2.3.1.12] | (RefSeq) dihydrolipoyllysine-residue acetyltransferase component 4 of pyruvate dehydrogenase complex, chloroplastic	Dihydrolipoyllysine-residue acetyltransferase component 4 of pyruvate dehydrogenase complex, chloroplastic OS=Arabidopsis thaliana OX=3702 GN=LTA2 PE=2 SV=1	-1.118
Cs4g14850	K06444|8.6e-73|cic:CICLE_v10008410mg|K06444 lycopene epsilon-cyclase [EC:5.5.1.18] | (RefSeq) hypothetical protein	Lycopene epsilon cyclase, chloroplastic OS=Arabidopsis thaliana OX=3702 GN=LUT2 PE=1 SV=2	-1.121
Cs5g28200	K03527|2.1e-60|cit:102610997|K03527 4-hydroxy-3-methylbut-2-en-1-yl diphosphate reductase [EC:1.17.7.4] | (RefSeq) 4-hydroxy-3-methylbut-2-enyl diphosphate reductase, chloroplastic-like isoform X1	4-hydroxy-3-methylbut-2-enyl diphosphate reductase, chloroplastic OS=Arabidopsis thaliana OX=3702 GN=ISPH PE=1 SV=1	-1.121
Cs4g17260	K15397|0.0e+00|cic:CICLE_v10007916mg|K15397 3-ketoacyl-CoA synthase [EC:2.3.1.199] | (RefSeq) hypothetical protein	3-ketoacyl-CoA synthase 11 OS=Arabidopsis thaliana OX=3702 GN=KCS11 PE=1 SV=1	1.194
Cs7g11300	K20619|2.2e-179|cic:CICLE_v10031237mg|K20619 cytochrome P450 family 78 subfamily A | (RefSeq) hypothetical protein	Cytochrome P450 78A5 OS=Arabidopsis thaliana OX=3702 GN=CYP78A5 PE=2 SV=1	1.670
Cs3g11490	K01738|3.2e-72|cic:CICLE_v10001793mg|K01738 cysteine synthase [EC:2.5.1.47] | (RefSeq) hypothetical protein	Cysteine synthase, chloroplastic/chromoplastic OS=Solanum tuberosum OX=4113 PE=2 SV=1	-1.300
Cs1g20920	K01610|1.9e-101|cit:102629129|K01610 phosphoenolpyruvate carboxykinase (ATP) [EC:4.1.1.49] | (RefSeq) phosphoenolpyruvate carboxykinase (ATP)-like	Phosphoenolpyruvate carboxykinase (ATP) 1 OS=Arabidopsis thaliana OX=3702 GN=PCK1 PE=1 SV=1	1.058
Cs5g10920	K01783|3.3e-19|adu:107472214|K01783 ribulose-phosphate 3-epimerase [EC:5.1.3.1] | (RefSeq) ribulose-phosphate 3-epimerase, chloroplastic	Ribulose-5-phosphate-3-epimerase, chloroplastic OS=Arabidopsis thaliana OX=3702 GN=RPE PE=2 SV=1	-1.001
Cs8g01360	K01885|8.0e-87|cit:102612397|K01885 glutamyl-tRNA synthetase [EC:6.1.1.17] | (RefSeq) glutamate--tRNA ligase, chloroplastic/mitochondrial	Glutamate--tRNA ligase, chloroplastic/mitochondrial OS=Arabidopsis thaliana OX=3702 GN=OVA3 PE=1 SV=1	-1.006
Cs9g01670	K00382|3.8e-234|cit:102627107|K00382 dihydrolipoamide dehydrogenase [EC:1.8.1.4] | (RefSeq) dihydrolipoyl dehydrogenase 1, mitochondrial isoform X1	Dihydrolipoyl dehydrogenase 1, mitochondrial OS=Arabidopsis thaliana OX=3702 GN=LPD1 PE=1 SV=2	-2.754
Cs2g17560	K00133|8.1e-100|cic:CICLE_v10015633mg|K00133 aspartate-semialdehyde dehydrogenase [EC:1.2.1.11] | (RefSeq) hypothetical protein	(Hypothetical protein CISIN_1g017153mg [Citrus sinensis])	-1.064
Cs4g07830	K02259|2.5e-61|cit:102630339|K02259 cytochrome c oxidase assembly protein subunit 15 | (RefSeq) cytochrome c oxidase assembly protein COX15	Cytochrome c oxidase assembly protein COX15 OS=Arabidopsis thaliana OX=3702 GN=COX15 PE=2 SV=1	-1.542

Cs1g22590	K13066|8.2e-73|cit:102629858|K13066 caffeic acid 3-O-methyltransferase [EC:2.1.1.68] | (RefSeq) caffeic acid 3-O-methyltransferase	Caffeic acid 3-O-methyltransferase OS=Eucalyptus gunnii OX=3933 GN=OMT PE=2 SV=1	-1.942
Cs2g13700	K01807|3.2e-144|cit:102627714|K01807 ribose 5-phosphate isomerase A [EC:5.3.1.6] | (RefSeq) probable ribose-5-phosphate isomerase 2	Probable ribose-5-phosphate isomerase 2 OS=Arabidopsis thaliana OX=3702 GN=RPI2 PE=1 SV=1	-1.305
Cs9g06700	K00276|4.5e-99|cic:CICLE_v10006614mg|K00276 primary-amine oxidase [EC:1.4.3.21] | (RefSeq) hypothetical protein	Primary amine oxidase 2 OS=Arabidopsis thaliana OX=3702 GN=At1g31672 PE=3 SV=1	1.927
Cs3g19820	K00128|2.9e-44|cit:102611460|K00128 aldehyde dehydrogenase (NAD+) [EC:1.2.1.3] | (RefSeq) aldehyde dehydrogenase family 3 member H1	Aldehyde dehydrogenase family 3 member H1 OS=Arabidopsis thaliana OX=3702 GN=ALDH3H1 PE=1 SV=2	-1.055
Cs6g07430	K03841|1.5e-138|cic:CICLE_v10011855mg|K03841 fructose-1,6-bisphosphatase I [EC:3.1.3.11] | (RefSeq) hypothetical protein	Fructose-1,6-bisphosphatase 1, chloroplastic OS=Arabidopsis thaliana OX=3702 GN=CFBP1 PE=1 SV=2	-1.619
Cs9g16520	K05282|4.5e-167|cit:102608767|K05282 gibberellin-44 dioxygenase [EC:1.14.11.12] | (RefSeq) gibberellin 20 oxidase 1	Gibberellin 20 oxidase 2 OS=Arabidopsis thaliana OX=3702 GN=GA20OX2 PE=2 SV=1	-2.607
orange1.1t05474	K01835|3.9e-26|cic:CICLE_v10014721mg|K01835 phosphoglucomutase [EC:5.4.2.2] | (RefSeq) hypothetical protein	Phosphoglucomutase, cytoplasmic OS=Mesembryanthemum crystallinum OX=3544 GN=PGM1 PE=2 SV=1	-1.607
Cs5g07120	K01940|1.1e-109|cit:102630538|K01940 argininosuccinate synthase [EC:6.3.4.5] | (RefSeq) argininosuccinate synthase, chloroplastic	Argininosuccinate synthase, chloroplastic OS=Arabidopsis thaliana OX=3702 GN=At4g24830 PE=1 SV=3	-1.032
orange1.1t06069	K00514|1.4e-32|cit:102577969|K00514 zeta-carotene desaturase [EC:1.3.5.6] | (RefSeq) ZDS	Zeta-carotene desaturase, chloroplastic/chromoplastic OS=Tagetes erecta OX=13708 PE=2 SV=1	1.528
Cs2g05100	K03405|6.0e-194|cit:102620862|K03405 magnesium chelatase subunit I [EC:6.6.1.1] | (RefSeq) magnesium-chelatase subunit ChlI, chloroplastic	Magnesium-chelatase subunit ChlI, chloroplastic OS=Glycine max OX=3847 GN=CHLI PE=2 SV=1	-1.158
Phenylpropanoid biosynthesis (cit00940; corrected P = 0.7323)			
Cs4g02360	K13065|8.6e-109|cit:102628720|K13065 shikimate O-hydroxycinnamoyltransferase [EC:2.3.1.133] | (RefSeq) shikimate O-hydroxycinnamoyltransferase-like	Shikimate O-hydroxycinnamoyltransferase OS=Nicotiana tabacum OX=4097 GN=HST PE=1 SV=1	-1.482
Cs7g01350	K01188|1.0e-74|cic:CICLE_v10031209mg|K01188 beta-glucosidase [EC:3.2.1.21] | (RefSeq) hypothetical protein	Beta-glucosidase 13 OS=Oryza sativa subsp. japonica OX=39947 GN=BGLU13 PE=2 SV=2	-1.095

orange1.1t02947	K13066|1.8e-102|cit:102620533|K13066 caffeic acid 3-O-methyltransferase [EC:2.1.1.68] | (RefSeq) anthranilate N-methyltransferase-like	Flavone 3'-O-methyltransferase 1 OS=Arabidopsis thaliana OX=3702 GN=OMT1 PE=1 SV=1	-3.099
Cs1g19540	K00430|8.3e-83|cit:102611617|K00430 peroxidase [EC:1.11.1.7] | (RefSeq) peroxidase 47	Peroxidase 47 OS=Arabidopsis thaliana OX=3702 GN=PER47 PE=2 SV=2	1.667
Cs2g10070	K22395|1.2e-311|cit:102618663|K22395 cinnamyl-alcohol dehydrogenase [EC:1.1.1.195] | (RefSeq) berberine bridge enzyme-like 13	Berberine bridge enzyme-like 13 OS=Arabidopsis thaliana OX=3702 GN=At1g30760 PE=1 SV=1	1.700
Cs1g22590	K13066|8.2e-73|cit:102629858|K13066 caffeic acid 3-O-methyltransferase [EC:2.1.1.68] | (RefSeq) caffeic acid 3-O-methyltransferase	Caffeic acid 3-O-methyltransferase OS=Eucalyptus gunnii OX=3933 GN=OMT PE=2 SV=1	-1.942
Vitamin B_6 metabolism (cit00750; corrected P = 0.0181)			
Cs1g14970	K06215|3.8e-168|cit:102610004|K06215 pyridoxal 5'-phosphate synthase pdxS subunit [EC:4.3.3.6] | (RefSeq) pyridoxal 5'-phosphate synthase-like subunit PDX1.2	Pyridoxal 5'-phosphate synthase-like subunit PDX1.2 OS=Arabidopsis thaliana OX=3702 GN=PDX12 PE=1 SV=1	-1.362
Cs1g18540	K13248|3.8e-85|cic:CICLE_v10026096mg|K13248 pyridoxal phosphate phosphatase PHOSPHO2 [EC:3.1.3.74] | (RefSeq) hypothetical protein	Inorganic pyrophosphatase 1 OS=Arabidopsis thaliana OX=3702 GN=PS2 PE=1 SV=1	3.003
Cs8g20230	K06215|4.4e-175|cic:CICLE_v10028683mg|K06215 pyridoxal 5'-phosphate synthase pdxS subunit [EC:4.3.3.6] | (RefSeq) hypothetical protein	Probable pyridoxal 5'-phosphate synthase subunit PDX1 OS=Hevea brasiliensis OX=3981 GN=PDX1 PE=2 SV=1	-1.356
Cs4g15090	K01733|2.2e-298|cit:102628627|K01733 threonine synthase [EC:4.2.3.1] | (RefSeq) threonine synthase 1, chloroplastic	Threonine synthase 1, chloroplastic OS=Arabidopsis thaliana OX=3702 GN=TS1 PE=1 SV=1	-1.164

Table S12. Differentially expressed genes (DEGs) related to ROS and aldehyde detoxification in low pH-treated *Citrus sinensis* leaves

Accession No.	KEGG_annotation	Swissprot_annotation (NR_annotation)	Log2(fold change)
Antioxidant activity (GO:0016209; adjusted P = 0.6065)			
Cs1g19540	K00430|8.3e-83|cit:102611617|K00430 peroxidase [EC:1.11.1.7] | (RefSeq) peroxidase 47	Peroxidase 47 OS=Arabidopsis thaliana OX=3702 GN=PER47 PE=2 SV=2	1.667
Cs3g12000	K04565|4.7e-25|cic:CICLE_v10002756mg|K04565 superoxide dismutase, Cu-Zn family [EC:1.15.1.1] | (RefSeq) hypothetical protein	Superoxide dismutase [Cu-Zn] 1 OS=Mesembryanthemum crystallinum OX=3544 GN=SODCC.1 PE=2 SV=1	1.988
Cs3g19810	K00434|8.8e-61|cit:102610460|K00434 L-ascorbate peroxidase [EC:1.11.1.11] | (RefSeq) probable L-ascorbate peroxidase 6, chloroplastic/mitochondrial isoform X1	Probable L-ascorbate peroxidase 6, chloroplastic/mitochondrial OS=Oryza sativa subsp. japonica OX=39947 GN=APX6 PE=2 SV=1	-1.375
Cs3g26690	K03671|1.0e-10|lsv:111897912|K03671 thioredoxin 1 | (RefSeq) thioredoxin H1-like	Thioredoxin-like protein CDSP32, chloroplastic OS=Arabidopsis thaliana OX=3702 GN=CDSP32 PE=1 SV=1	-1.219
Cs7g19240	K04564|1.7e-45|cic:CICLE_v10032233mg|K04564 superoxide dismutase, Fe-Mn family [EC:1.15.1.1] | (RefSeq) hypothetical protein	Superoxide dismutase [Fe] 2, chloroplastic OS=Arabidopsis thaliana OX=3702 GN=FSD2 PE=1 SV=1	-1.606
Cs8g15520	K04565|1.9e-44|cit:102626642|K04565 superoxide dismutase, Cu-Zn family [EC:1.15.1.1] | (RefSeq) superoxide dismutase [Cu-Zn], chloroplastic	Superoxide dismutase [Cu-Zn], chloroplastic OS=Petunia hybrida OX=4102 GN=SODCP PE=2 SV=1	2,676
Sulfur compound metabolic process (GO:0006790, adjusted P =0.9726)			
Cs2g17560	K00133|8.1e-100|cic:CICLE_v10015633mg|K00133 aspartate-semialdehyde dehydrogenase [EC:1.2.1.11] | (RefSeq) hypothetical protein	(Hypothetical protein CISIN_1g017153mg [Citrus sinensis])	-1.064
Cs2g30390	K00392|1.5e-205|cic:CICLE_v10014382mg|K00392 sulfite reductase (ferredoxin) [EC:1.8.7.1] | (RefSeq) hypothetical protein	Sulfite reductase [ferredoxin], chloroplastic (Fragment) OS=Glycine max OX=3847 GN=SIR PE=1 SV=1	-1.069
Cs3g11490	K01738|3.2e-72|cic:CICLE_v10001793mg|K01738 cysteine synthase [EC:2.5.1.47] | (RefSeq) hypothetical protein	Cysteine synthase, chloroplastic/chromoplastic OS=Solanum tuberosum OX=4113 PE=2 SV=1	-1.300
Cs7g16390	K22846|7.4e-82|cic:CICLE_v10031768mg|K22846 S-sulfo-L-cysteine synthase (O-acetyl-L-serine-dependent) [EC:2.5.1.144] | (RefSeq) hypothetical protein	S-sulfo-L-cysteine synthase (O-acetyl-L-serine-dependent), chloroplastic OS=Arabidopsis thaliana OX=3702 GN=CS26 PE=1 SV=1	-2.495

orange1.1t01892	K01251|1.7e-141|cit:102617645|K01251 adenosylhomocysteinase [EC:3.3.1.1] | (RefSeq) adenosylhomocysteinase	Adenosylhomocysteinase OS=Catharanthus roseus OX=4058 GN=SAHH PE=2 SV=1	-1.124
Sulfur amino acid biosynthetic process (GO:0000097, adjusted P = 0.3530)			
Cs2g17560	K00133|8.1e-100|cic:CICLE_v10015633mg|K00133 aspartate-semialdehyde dehydrogenase [EC:1.2.1.11] | (RefSeq) hypothetical protein	(Hypothetical protein CISIN_1g017153mg [Citrus sinensis])	-1.064
Cs2g30390	K00392|1.5e-205|cic:CICLE_v10014382mg|K00392 sulfite reductase (ferredoxin) [EC:1.8.7.1] | (RefSeq) hypothetical protein	Sulfite reductase [ferredoxin], chloroplastic (Fragment) OS=Glycine max OX=3847 GN=SIR PE=1 SV=1	-1.069
Cs3g11490	K01738|3.2e-72|cic:CICLE_v10001793mg|K01738 cysteine synthase [EC:2.5.1.47] | (RefSeq) hypothetical protein	Cysteine synthase, chloroplastic/chromoplastic OS=Solanum tuberosum OX=4113 PE=2 SV=1	-1.300
Cs7g16390	K22846|7.4e-82|cic:CICLE_v10031768mg|K22846 S-sulfo-L-cysteine synthase (O-acetyl-L-serine-dependent) [EC:2.5.1.144] | (RefSeq) hypothetical protein	S-sulfo-L-cysteine synthase (O-acetyl-L-serine-dependent), chloroplastic OS=Arabidopsis thaliana OX=3702 GN=CS26 PE=1 SV=1	-2.495
Glutathione metabolism (cit00480, corrected P = 0.8566)			
Cs3g19810	K00434|8.8e-61|cit:102610460|K00434 L-ascorbate peroxidase [EC:1.11.1.11] | (RefSeq) probable L-ascorbate peroxidase 6, chloroplastic/mitochondrial isoform X1	Probable L-ascorbate peroxidase 6, chloroplastic/mitochondrial OS=Oryza sativa subsp. japonica OX=39947 GN=APX6 PE=2 SV=1	-1.375
Cs7g11110	K00036|2.2e-114|cit:102624333|K00036 glucose-6-phosphate 1-dehydrogenase [EC:1.1.1.49 1.1.1.363] | (RefSeq) glucose-6-phosphate 1-dehydrogenase, chloroplastic isoform X1	Glucose-6-phosphate 1-dehydrogenase, chloroplastic OS=Nicotiana tabacum OX=4097 PE=2 SV=1	-1.151
Phytochelatin biosynthetic process (GO:0046938, adjusted P = 0.5264)			
Cs5g29460	K05941|7.2e-112|cic:CICLE_v10024202mg|K05941 glutathione gamma-glutamylcysteinyltransferase [EC:2.3.2.15] | (RefSeq) hypothetical protein	Glutathione gamma-glutamylcysteinyltransferase 2 OS=Lotus japonicus OX=34305 GN=PCS2 PE=2 SV=2	4.419
Sulfur compound transport (GO:0072348, adjusted P = 0.7303)			
Cs1g01730	K17469|1.0e-119|cic:CICLE_v10025101mg|K17469 sulfate transporter 2, low-affinity | (RefSeq) hypothetical protein	Sulfate transporter 2.1 OS=Arabidopsis thaliana OX=3702 GN=SULTR2;1 PE=2 SV=1	1.442

Ascorbate and aldarate metabolism (cit00053; corrected P = 0.0756)			
Cs3g19820	K00128|2.9e-44|cit:102611460|K00128 aldehyde dehydrogenase (NAD+) [EC:1.2.1.3] | (RefSeq) aldehyde dehydrogenase family 3 member H1	Aldehyde dehydrogenase family 3 member H1 OS=Arabidopsis thaliana OX=3702 GN=ALDH3H1 PE=1 SV=2	-1.055
Cs1g16030	K00469|1.6e-45|cit:102618274|K00469 inositol oxygenase [EC:1.13.99.1] | (RefSeq) inositol oxygenase 1 isoform X1	Inositol oxygenase 1 OS=Arabidopsis thaliana OX=3702 GN=MIOX1 PE=2 SV=1	-2.998
Cs2g27950	K08232|1.7e-134|cit:102611741|K08232 monodehydroascorbate reductase (NADH) [EC:1.6.5.4] | (RefSeq) monodehydroascorbate reductase 4, peroxisomal	Monodehydroascorbate reductase 4, peroxisomal OS=Arabidopsis thaliana OX=3702 GN=MDAR4 PE=1 SV=1	-1.025
Cs3g19810	K00434|8.8e-61|cit:102610460|K00434 L-ascorbate peroxidase [EC:1.11.1.11] | (RefSeq) probable L-ascorbate peroxidase 6, chloroplastic/mitochondrial isoform X1	Probable L-ascorbate peroxidase 6, chloroplastic/mitochondrial OS=Oryza sativa subsp. japonica OX=39947 GN=APX6 PE=2 SV=1	-1.375
Cs2g27180	K16190|4.9e-76|cic:CICLE_v10015593mg|K16190 glucuronokinase [EC:2.7.1.43] | (RefSeq) hypothetical protein	Glucuronokinase 1 OS=Arabidopsis thaliana OX=3702 GN=GLCAK1 PE=1 SV=1	2.430
Vitamin B_6 metabolism (cit00750; corrected P = 0.0181)			
Cs1g14970	K06215|3.8e-168|cit:102610004|K06215 pyridoxal 5'-phosphate synthase pdxS subunit [EC:4.3.3.6] | (RefSeq) pyridoxal 5'-phosphate synthase-like subunit PDX1.2	Pyridoxal 5'-phosphate synthase-like subunit PDX1.2 OS=Arabidopsis thaliana OX=3702 GN=PDX12 PE=1 SV=1	-1.362
Cs1g18540	K13248|3.8e-85|cic:CICLE_v10026096mg|K13248 pyridoxal phosphate phosphatase PHOSPHO2 [EC:3.1.3.74] | (RefSeq) hypothetical protein	Inorganic pyrophosphatase 1 OS=Arabidopsis thaliana OX=3702 GN=PS2 PE=1 SV=1	3.003
Cs8g20230	K06215|4.4e-175|cic:CICLE_v10028683mg|K06215 pyridoxal 5'-phosphate synthase pdxS subunit [EC:4.3.3.6] | (RefSeq) hypothetical protein	Probable pyridoxal 5'-phosphate synthase subunit PDX1 OS=Hevea brasiliensis OX=3981 GN=PDX1 PE=2 SV=1	-1.356
Cs4g15090	K01733|2.2e-298|cit:102628627|K01733 threonine synthase [EC:4.2.3.1] | (RefSeq) threonine synthase 1, chloroplastic	Threonine synthase 1, chloroplastic OS=Arabidopsis thaliana OX=3702 GN=TS1 PE=1 SV=1	-1.164
Vitamin E biosynthetic process (GO:0010189, adjusted P = 0.4324)			
Cs5g10740	K10960|3.6e-141|cic:CICLE_v10020061mg|K10960 geranylgeranyl diphosphate/geranylgeranyl-bacteriochlorophyllide a reductase [EC:1.3.1.83 1.3.1.111] | (RefSeq) hypothetical protein	Geranylgeranyl diphosphate reductase, chloroplastic OS=Arabidopsis thaliana OX=3702 GN=CHLP PE=1 SV=1	-1.716

Pentose phosphate pathway (cit00030; corrected P = 0.0029)			
orange1.1t00226	K00615|1.8e-253|cic:CICLE_v10018991mg|K00615 transketolase [EC:2.2.1.1] | (RefSeq) hypothetical protein	Transketolase, chloroplastic OS=Zea mays OX=4577 PE=1 SV=1	-1.337
Cs3g10480	K01807|1.9e-150|cic:CICLE_v10002135mg|K01807 ribose 5-phosphate isomerase A [EC:5.3.1.6] | (RefSeq) hypothetical protein	Probable ribose-5-phosphate isomerase 3, chloroplastic OS=Arabidopsis thaliana OX=3702 GN=RPI3 PE=1 SV=1	-1.730
Cs7g11110	K00036|2.2e-114|cit:102624333|K00036 glucose-6-phosphate 1-dehydrogenase [EC:1.1.1.49 1.1.1.363] | (RefSeq) glucose-6-phosphate 1-dehydrogenase, chloroplastic isoform X1	Glucose-6-phosphate 1-dehydrogenase, chloroplastic OS=Nicotiana tabacum OX=4097 PE=2 SV=1	-1.151
Cs5g10920	K01783|3.3e-19|adu:107472214|K01783 ribulose-phosphate 3-epimerase [EC:5.1.3.1] | (RefSeq) ribulose-phosphate 3-epimerase, chloroplastic	Ribulose-5-phosphate-3-epimerase, chloroplastic OS=Arabidopsis thaliana OX=3702 GN=RPE PE=2 SV=1	-1.001
Cs2g13700	K01807|3.2e-144|cit:102627714|K01807 ribose 5-phosphate isomerase A [EC:5.3.1.6] | (RefSeq) probable ribose-5-phosphate isomerase 2	Probable ribose-5-phosphate isomerase 2 OS=Arabidopsis thaliana OX=3702 GN=RPI2 PE=1 SV=1	-1.305
Cs6g07430	K03841|1.5e-138|cic:CICLE_v10011855mg|K03841 fructose-1,6-bisphosphatase I [EC:3.1.3.11] | (RefSeq) hypothetical protein	Fructose-1,6-bisphosphatase 1, chloroplastic OS=Arabidopsis thaliana OX=3702 GN=CFBP1 PE=1 SV=2	-1.619
orange1.1t05474	K01835|3.9e-26|cic:CICLE_v10014721mg|K01835 phosphoglucomutase [EC:5.4.2.2] | (RefSeq) hypothetical protein	Phosphoglucomutase, cytoplasmic OS=Mesembryanthemum crystallinum OX=3544 GN=PGM1 PE=2 SV=1	-1.607
Cs2g17050	K01835|1.1e-16|cic:CICLE_v10014721mg|K01835 phosphoglucomutase [EC:5.4.2.2] | (RefSeq) hypothetical protein	Probable phosphoglucomutase, cytoplasmic 1 OS=Arabidopsis thaliana OX=3702 GN=At1g23190 PE=2 SV=2	-1.761
Others			
Cs3g01240	K01800|1.4e-22|cic:CICLE_v10002464mg|K01800 maleylacetoacetate isomerase [EC:5.2.1.2] | (RefSeq) hypothetical protein	Glutathione S-transferase zeta class OS=Euphorbia esula OX=3993 PE=2 SV=1	1.712
orange1.1t03055	K00002|1.2e-61|cic:CICLE_v10012243mg|K00002 alcohol dehydrogenase (NADP+) [EC:1.1.1.2] | (RefSeq) hypothetical protein	NADPH-dependent aldo-keto reductase, chloroplastic OS=Arabidopsis thaliana OX=3702 GN=AKR4C9 PE=1 SV=1	-1.133
orange1.1t04575	K19825|1.5e-53|cann:107868137|K19825 2-alkenal reductase (NADP+) [EC:1.3.1.102] | (RefSeq) 2-alkenal reductase (NADP(+)-dependent)	2-alkenal reductase (NADP(+)-dependent) OS=Nicotiana tabacum OX=4097 GN=DBR PE=1 SV=1	-1.431

Table S13. Specific primer pairs used for qRT-PCR analysis

ID	Description	Forward primers (5′→3′)	Reverse primers (5′→3′)
Cs1g05000	*Actin*	AGAACTATGAACTGCCTGATGGC	GCTTGGAGCAAGTGCTGTGATT
Cs6g07480	*B-tublin*	CCCTTTACGACATCTGTTTCCG	TGGCATCCCACATTTGCTG
Cs7g08440	*U4/U6 small nuclear ribonucleoprotein PRP31 (PRPF31)*	ACTCATGGGAACGGCTGGTGGTC	TCGGCAGGCACGCATCCTTAGAG
Cs1g01730	*sulfate transporter 2.1*	ATACCGCCTTGATGGAAACA	GCACTGCGTGAGAATGAACC
Cs1g24500	*Probable mitochondrial chaperone BCS1-B*	ATTCGGTCAAACTTGAGCATC	ATCGGAGTCACGGGTAACAT
Cs2g04080	*ABC transporter B family member 9*	TCAACTCTTTCGGCTCCTCTG	GCCTTTCCCCTGTCACCAT
Cs2g05100	*Magnesium-chelatase subunit ChlI*	GTGATAGAGGAACGGGGAAAT	TGTAGCACCCAATGGCAAA
Cs2g20580	*Probable tyrosine-protein phosphatase*	AAGTCGCATCCGTCGTTG	GAAAGGAAAAGTTGGCAGAGTC
Cs3g01240	*Glutathione S-transferase zeta class*	CGGCTTTGGTTGATGGAGA	AGATCAGAAGGCAACAATGGA
Cs3g12000	*Superoxide dismutase [Cu-Zn]*	AAGGGCTGTTGTAGTCCACG	ATGCCGCAAGCTACTCTGC
Cs4g06150	*Cytokinin dehydrogenase 7*	GGATGCTGAGTTGCTGGTGA	CGGCGGTTTGAGGTAAGTG
Cs4g15360	*Protein phosphatase 2C 56*	AGGACCGATGAGGATGAGTGT	GCGTCGCCTTCTTAGCAA
Cs4g18140		GCGGAGACTTACACTGTTGGA	ATTTGCAGGGCTAGTTTCATG
Cs4g18450	*Fe (2+) transport protein 3,*	GCGGCTCACCATAGACATAAT	CCAAAACCTGGGAAACGAC
Cs4g18620		GCATCTTGGCGTGCTGTT	TAGGGGTATCGCTATGGGAAT
Cs5g10740	*Geranylgeranyl diphosphate reductase, chloroplastic*	GATGGATTTGCCGAGGAAC	CATCAACCTCCAGCGTCCT
Cs5g29460	*Glutathione gamma-glutamylcysteinyltransferase 3*	ATGGCAATGGCAGGACTTTA	GTCCACAATACGCAGGCTCT
Cs6g06020		CGGCAACCAGCAGCATT	ATCCACCCCACTTCCTCAT
Cs6g06030		TTGGCAGGGAGAAGAAGGTT	GACGCAAGGGTTTGTAGATGG
Cs6g09160	*Mitoferrin*	GTGGGGTCCCTAATAACTCAATG	TGGGCGTAATCACAGCATC
Cs8g19450	*Copper transporter 1*	CCCGCCACAAAATGATGA	CCGTGCCCGTTTTGATC
Cs8g03750		GCATTTCCCGATGTTACTCC	CTATGTGAGCGGCAAGTGTTC
Cs8g15520	*Superoxide dismutase [Cu-Zn]*	TGCTGCCGCCAAGAAAG	CAGTCGTAGGACCGCCATCT
Cs8g16920	*MLO-like protein 3*	GCCTTGCCATCAGAGTCCA	TTCCCGTAAAGAATCAGTCCAT
Cs8g19460	*Copper transporter 1*	TGGGGCAAGAACTCTGAAAT	AAGCAAGCCCAACCCTGA
Cs9g18020	*Probable protein phosphatase 2C 51*	AAGAAGGCACGCACTAATACTG	CCCCATACACCCTAACTCAACT

orange1.1t01343	*Calcium-transporting ATPase 9*	TGTCTTCACGGGAGTTACAAAA	GAGTCTTCGGAACTGGAATCAT
orange1.1t01850	*Two-component response regulator ARR9*	AATCGCAACGGCAACAA	CCTCTTCCAGGGCTTTTCTC
orange1.1t02212		CTGGAATTAAACTTGCCCTGTG	GCCGCAATCAAACCAACC
